# Targeting ubiquitin-independent proteasome with small molecule increases susceptibility in pan-KRAS–mutant cancer models

**DOI:** 10.1172/JCI185278

**Published:** 2025-03-17

**Authors:** Shihui Shen, Qiansen Zhang, Yuhan Wang, Hui Chen, Shuangming Gong, Yun Liu, Conghao Gai, Hansen Chen, Enhao Zhu, Bo Yang, Lin Liu, Siyuan Cao, Mengting Zhao, Wenjie Ren, Mengjuan Li, Zhuoya Peng, Lu Zhang, Shaoying Zhang, Juwen Shen, Bianhong Zhang, Patrick K.H. Lee, Kun Li, Lei Li, Huaiyu Yang

**Affiliations:** 1 Shanghai Key Laboratory of Regulatory Biology, Institute of Biomedical Sciences, School of Life Sciences, East China Normal University, Shanghai, China.; 2 Joint Center for Translational Medicine, Shanghai Fifth People’s Hospital, Fudan University and School of Life Science, East China Normal University, Shanghai, China.; 3 Organic Chemistry Group, College of Pharmacy, Naval Medical University, Shanghai, China.; 4 School of Energy and Environment, City University of Hong Kong, Hong Kong SAR, China.; 5 Health Science Center, East China Normal University, Shanghai, China.

**Keywords:** Oncology, Therapeutics, Cancer, Drug therapy, Tumor suppressors

## Abstract

Despite advances in the development of direct KRAS inhibitors, KRAS-mutant cancers continue to exhibit resistance to the currently available therapies. Here, we identified REGγ as a mutant KRAS–associated factor that enhanced REGγ transcription through the KRAS intermediate NRF2, suggesting that the REGγ-proteasome is a potential target for pan-KRAS inhibitor development. We elucidated a mechanism involving the KRAS/NRF2/REGγ regulatory axis, which links activated KRAS to the ATP- and ubiquitin-independent proteasome. We subsequently developed RLY01, a REGγ-proteasome inhibitor that effectively suppressed tumor growth in KRAS-mutant cancer models and lung cancer organoids. Notably, the combination of RLY01 and the *KRAS^G12C^* inhibitor AMG510 exhibited enhanced antitumor efficacy in *KRAS^G12C^* cancer cells. Collectively, our data support the hypothesis that KRAS mutations enhance the capacity of the REGγ-proteasome by increasing REGγ expression, highlighting the potential of ubiquitin-independent proteasome inhibition as a therapeutic approach for pan-KRAS–mutant cancers.

## Abstract 2

<a>Small molecule inhibition of the ubiquitin-independent proteasome is a potential therapeutic strategy for increasing susceptibility in pan-KRAS mutant cancers</a>

## Introduction

KRAS is one of the most commonly mutated oncogenes in human cancers, accounting for 92% of pancreatic ductal adenocarcinomas, 49% of colorectal carcinomas, and 35% of lung adenocarcinomas (1–3). KRAS mutations have been shown to exert transformative capacity in aggressive cancers by hyperactivating various downstream signaling pathways, such as the MAPK and PI3K/AKT pathways (4). Although KRAS was previously viewed as a challenging drug target owing to its picomolar binding affinity for GTP/GDP and its lack of well-defined drug-binding pockets, two targeted therapeutic agents for *KRAS^G12C^* have transformed therapeutic strategies for tumors harboring this mutation (5). One of these *KRAS^G12C^* inhibitors, sotorasib (AMG510) (6, 7), has been approved for clinical use and has provided clinical benefits in patients with *KRAS^G12C^*-mutant non–small cell lung cancer, but its efficacy in patients with colorectal and pancreatic cancers harboring the *KRAS^G12C^* mutation is still in the early stages of evaluation (8, 9). Additionally, inhibitors of other KRAS mutants, such as MRTX1133 (targeting *KRAS^G12D^*) and pan-KRAS inhibitors, are being developed (10, 11). However, most currently available drugs bind to the same pocket on mutant KRAS, resulting in the stabilization of GDP-bound inactive state. Furthermore, *KRAS^G12C^* inhibitors cover only a small fraction of all KRAS mutants, whereas several *KRAS^G12D^* inhibitors for KRAS mutation–related cancers are currently undergoing clinical trials (12, 13). Owing to the occurrence of secondary KRAS mutations and adaptive feedback mechanisms in response to KRAS inhibition, both primary and acquired resistance is common, limiting clinical efficacy (14, 15). Therefore, it is imperative to develop alternative options for treating KRAS-mutant cancers.

KRAS mutations are associated with a heightened predilection for proteasome activity. The elevated rates of protein synthesis and rapid protein turnover required by KRAS-activating mutations in cancer cells lead to a greater dependence on the proteasome system, which is crucial for maintaining protein homeostasis through degradation (16, 17). Recent studies have shown that proteasome inhibition synergistically enhances sensitivity to both the *KRAS^G12C^* inhibitor AMG510 and the *KRAS^G12D^* inhibitor HRS-4642 in lung cancer and pancreatic ductal adenocarcinoma cell lines (13, 18). This evidence indicates that the proteasome is a potential drug target in KRAS-mutant cancer cells. However, FDA-approved broad-spectrum proteasome inhibitors, such as bortezomib (also known as Velcade) (19) and carfilzomib (20), can induce high cytotoxicity and off-target effects, ultimately resulting in unsatisfactory therapeutic efficacy (21). Although proteasome inhibitors have been used clinically for decades, the complex mechanisms underlying their suboptimal efficacy in solid tumors are not fully understood. Thus, given the high proteasome capacity that is characteristic of KRAS-mutant tumors, there is an urgent need to develop selective and low-toxicity proteasome inhibitors for optimal therapy in KRAS-mutant cancers.

In our study, we investigated the differences between wild-type and mutant KRAS isogenic cells through proteomic analysis, identifying REGγ as a key protein whose expression is markedly altered in KRAS-mutant cancer cells. REGγ is an 11S proteasome regulator that functions by binding to the 20S proteasome, allosterically activating the trypsin-like proteolytic site (β_2_ subunit) (22), and facilitating protein degradation in an ATP- and ubiquitin-independent manner. Our previous research revealed that REGγ is widely recognized for its role in tumorigenesis and is overexpressed in several solid tumors, including colon, lung, and pancreatic cancers (23–31). Another study demonstrated that REGγ accelerates lung cancer progression and metastasis in *Kras^G12D^* *Trp53^fl/fl^* mice, a model of non–small cell lung cancer (32). These findings suggest that REGγ plays a vital role in KRAS-mutant cancers. Here, we found that NRF2 and REGγ were upregulated upon KRAS activation. We further observed that NRF2 directly interacted with a particular motif of the *REGγ* promoter to enhance the function of the REGγ-proteasome by increasing REGγ expression. We demonstrated that the KRAS/NRF2/REGγ signaling axis directly upregulated REGγ expression in the context of KRAS mutations. Through chemical screening and validation assays, we identified RLY01 as a potent small-molecule inhibitor of REGγ-20S proteasome that blocked its degradation function, ultimately leading to cancer cell death. We revealed that RLY01, with low toxicity, markedly impeded the growth of KRAS-mutant lung cancer organoids in vitro and tumor development in several preclinical mouse models in vivo, including colorectal cancer lung cancer xenografts. More importantly, the combination of RLY01 and AMG510 resulted in synergistic antitumor effects in *KRAS^G12C^* lung cancers, demonstrating the clinical potential of RLY01. These findings highlight the potential of targeting the REGγ-20S proteasome for the treatment of pan-KRAS–mutant cancers.

## Results

### REGγ is overexpressed in KRAS-mutant cancers and its expression is correlated with specific KRAS-mutant subtypes.

**Figure 1:**
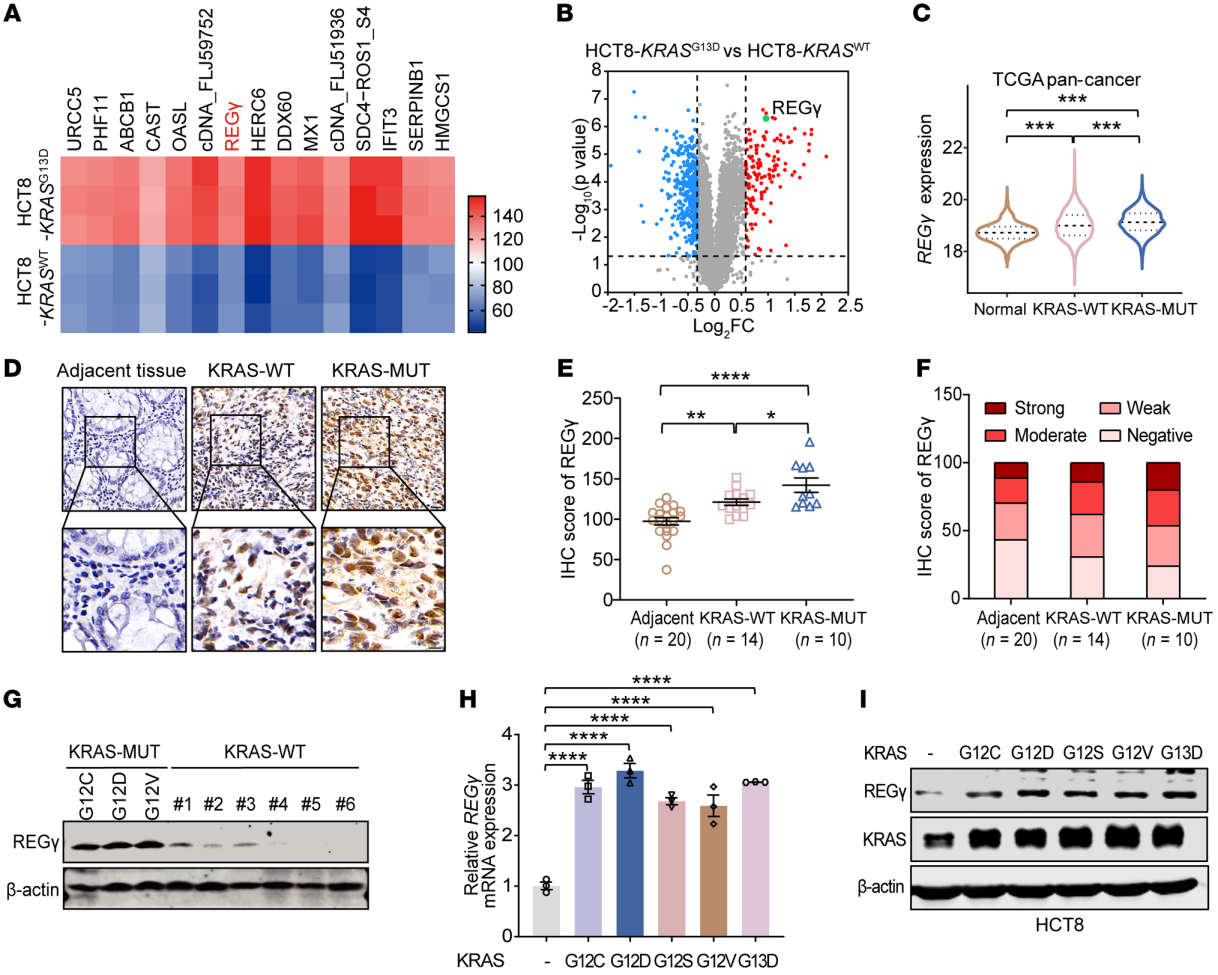
REGγ is overexpressed in KRAS-mutant cancers and correlated with specific KRAS-mutant subtypes. (**A**) Heatmap showing markedly expressed proteins (*P* < 0.05) between HCT8-*KRAS^WT^* and HCT8-*KRAS^G13D^* groups (*n* = 3). (**B**) Volcano plot showing markedly expressed proteins (*P* < 0.05, fold change <0.8 or fold change >1.5) between HCT8-*KRAS^WT^* and HCT8-*KRAS^G13D^* groups (*n* = 3). (**C**) Violin plots depicting distribution of REGγ expression level in normal tissues (*n* = 741), KRAS-WT (wild-type) cancer tissues (*n* = 8,661), and KRAS-MUT (mutant) cancer tissues (*n* = 769). Datasets of pan-cancer were derived from TCGA. ***P < 0.001. (**D**) Representative IHC images of REGγ expression in KRAS-WT and KRAS-MUT colon cancer tissues. Bottom: A higher magnification of sections. Scale bars: 20 μm (top), 10 μm (bottom). (**E**) REGγ staining scores are shown. Each value represents mean ± SEM (*n* = 3). *P < 0.05, **P < 0.01, ****P < 0.0001. (**F**) Quantitative analysis of REGγ IHC staining for adjacent normal tissue (*n* = 20), KRAS-WT (*n* = 14), and KRAS-MUT (*n* = 10). IHC signals were classified as negative, weak, moderate, or strong. (**G**) Western blot images showing high REGγ expression in KRAS-MUT lung cancer tissues. Each lane represents a tissue sample from an individual patient. (**H** and **I**) REGγ mRNA (**H**) and protein (**I**) levels were upregulated with the overexpression of KRAS mutants. A panel of KRAS mutant plasmids (*KRAS^G12C^*, *KRAS^G12D^*, *KRAS^G12S^*, *KRAS^G12V^*, and *KRAS^G13D^*) in was transfected into HCT8 cells for 48 hours. Each value represents mean ± SEM (*n* = 3). ****P < 0.0001; *P* values were measured by 1-way ANOVA with Tukey’s multiple-comparison test.

First, we performed proteomic analysis using the tandem mass tag (TMT) labeling quantification method to compare a KRAS-mutant colon cancer cell line (HCT8-*KRAS^G13D^*) with a KRAS–wild type colon cancer cell line (HCT8-*KRAS^WT^*) (Supplemental Figure 1A; supplemental material available online with this article; https://doi.org/10.1172/JCI185278DS1). Bioinformatics analysis revealed that 140 proteins had upregulated expression and 434 proteins had downregulated expression in HCT8-*KRAS^G13D^* cells compared with HCT8-*KRAS^WT^* cells (fold change > 1.5; adjusted *P* value < 0.05; Figure 1A shows the top 15 proteins with differential expression, Figure 1B shows the striking differentially expressed proteins). Notably, we observed dramatic upregulation of REGγ expression in pan-KRAS–mutant tumors, which exceeded that of the other top 10 identified proteins (Supplemental Figure 1B). Moreover, we observed a strong expression of REGγ in KRAS-mutant (KRAS-MUT) cancers, which was supported by data from The Cancer Genome Atlas (TCGA) datasets (Figure 1C).

REGγ has been implicated as an oncogene that promotes malignant transformation in KRAS-mutant lung cancer (23, 24, 31). To investigate the oncogenic relevance of REGγ in KRAS-mutant cancers, we obtained isogenic human colon and lung cancer cells (HCT8, H661, HPNE, and H522) through the introduction of the constitutively active *KRAS^G13D^* mutation. The mRNA levels of REGγ were elevated in HCT8-*KRAS^G13D^* cells, and the protein expression levels of REGγ were markedly increased in H661-*KRAS^G13D^*, HPNE-*KRAS^G13D^*, and H522-*KRAS^G13D^* cells (Supplemental Figure 1, C–E). Similar results were obtained in isolated mouse embryonic fibroblasts (Supplemental Figure 1F). To further support these findings, we examined REGγ protein expression in different KRAS-mutant cancer cell lines. Our Western blot analysis (Supplemental Figure 1, G and H) revealed an increase in REGγ expression in KRAS-MUT cells compared with KRAS-WT and normal cells. Next, we analyzed REGγ and KRAS-GTP protein expression across various human lung and colorectal cancer cell lines. The protein expression of REGγ was robustly elevated in the presence of activating KRAS mutations (Supplemental Figure 1, I–K), reinforcing the general role of REGγ in response to different KRAS mutant variants. The upregulation of REGγ further augmented the degradation activity of the REGγ-proteasome (Supplemental Figure 1K).

To determine the connection between REGγ expression and clinical outcomes in cancers with KRAS mutations, we examined REGγ expression in human colon cancer tissues, including the KRAS-MUT subgroup (*n* = 10) and the KRAS-WT subgroup (*n* = 14). Immunohistochemical analysis revealed notably greater REGγ protein expression in the KRAS-MUT subgroup than in both the KRAS-WT subgroup and adjacent normal tissue (*n* = 20; Figure 1, D and E). Notably, 76% of the KRAS-MUT lesions presented positive REGγ staining, with 56% presenting moderate or strong staining. In contrast, the majority of the lesions in the KRAS-WT subgroup and adjacent normal colon tissues exhibited weak or no REGγ staining (Figure 1F), indicating a pattern of specific REGγ expression in KRAS-mutant colon cancers.

Correspondingly, Western blot analysis revealed high levels of REGγ expression in human lung cancer cells harboring KRAS mutations (Figure 1G). Additionally, we observed that, compared with cells without KRAS mutations, HCT8 cells expressing various KRAS mutations exhibited substantial increases in both the mRNA and protein levels of REGγ (Figure 1, H and I). Moreover, analysis of human colorectal carcinoma datasets obtained from TCGA revealed a positive correlation between REGγ expression and KRAS expression (Supplemental Figure 1L). Together, these findings suggest that REGγ is highly expressed in pan-KRAS–mutant tumors and indicate that REGγ may be an independent risk factor or a vulnerability factor in patients with such cancers, potentially impacting malignancy and patient prognosis.

### Pan-KRAS–mutant cells exhibit selective sensitivity to REGγ inhibition.

**Figure 2:**
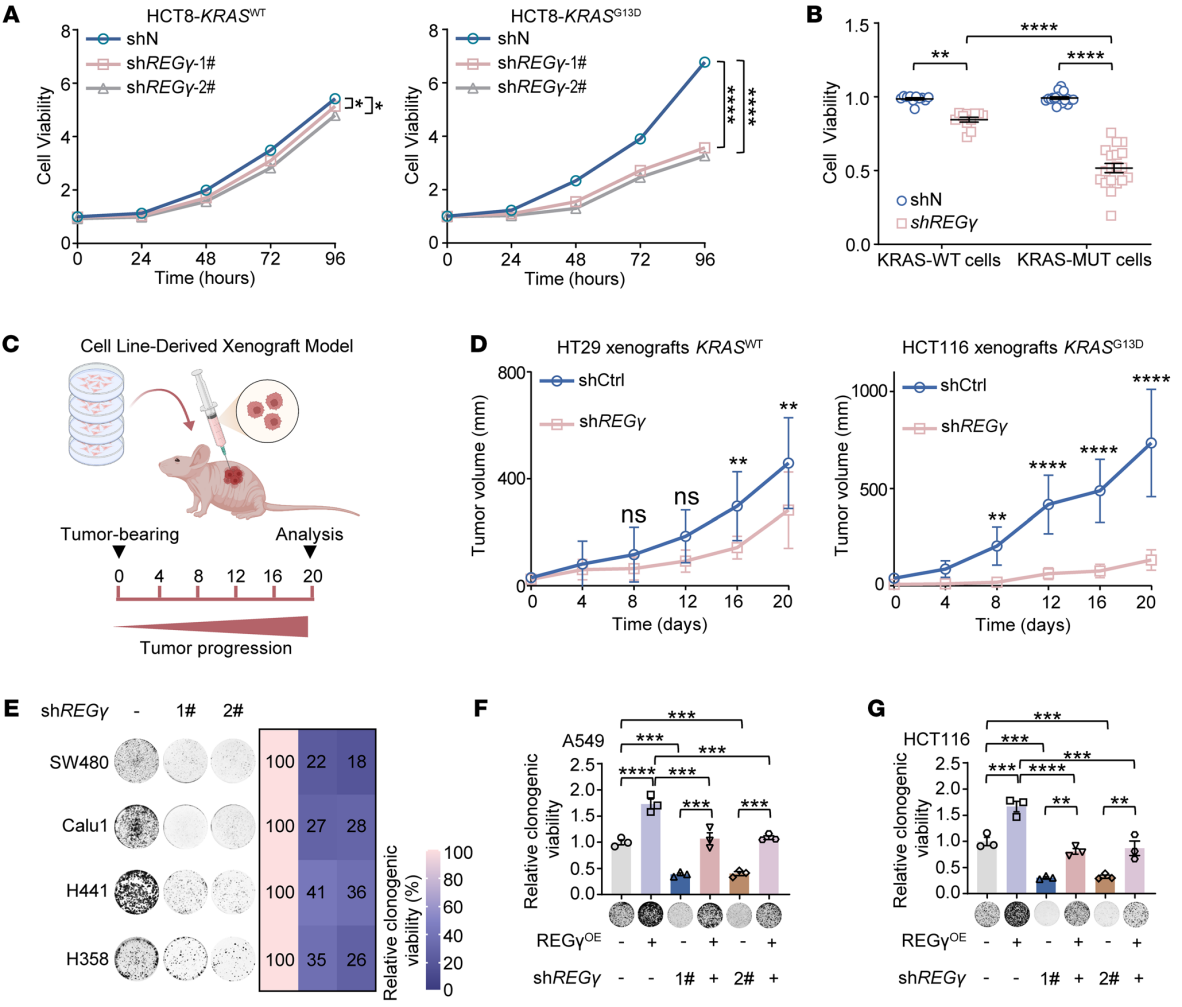
Pan-KRAS–mutant cells exhibit selective sensitivity to REGγ inhibition. (**A**) Relative cell viability of HCT8-*KRAS^WT^* (left) or HCT8-*KRAS^G13D^* (right) cells with or without REGγ knockdown. Relative cell viability was calculated by setting the values of the shN group (a negative control which was transfected a scramble shRNA) as 100%. Each value represents mean ± SEM (*n* = 3). *P < 0.05, ****P < 0.0001; *P* values were measured by 2-way ANOVA with Šidák’s multiple-comparison test. (**B**) REGγ depletion led to selective toxicity toward KRAS-mutant cancer cell lines. Nineteen KRAS-MUT and eleven KRAS-WT cancer cell lines were transfected with sh*REGγ* or a scrambled shRNA. The percentage cell viability is relative to the untreated controls. Each value represents mean ± SEM (*n* = 3). **P < 0.01, ****P < 0.0001; *P* values were measured by 2-way ANOVA with Šidák’s multiple-comparison test. (**C**) Schematic illustration of the mouse protocol using xenografts derived from colorectal cell lines (HT29, HCT116). (**D**) Growth curves for HT29 and HCT116 xenografts with or without silencing of *REGγ* (*n* = 8). Values represent mean ± SEM. **P < 0.01, ****P < 0.0001; *P* values were measured by 2-way ANOVA with Tukey’s multiple-comparison test. (**E**) Representative colony formation images (left) and relative colony numbers (right) of KRAS-MUT cells with or without *REGγ* knockdown. (**F** and **G**) Ectopically expressed REGγ restored the clonogenic growth of REGγ-depleted cells. REGγ^OE^, REGγ overexpression. The relative viability of cultured colonies in A549 (*KRAS^G12S^*) and HCT116 (*KRAS^G13D^*) cells is shown. The percentage cell viability is relative to the untreated controls. Each value represents mean ± SEM (*n* = 3). **P < 0.01, ***P < 0.001, ****P < 0.0001; *P* values were measured by 2-way ANOVA with Tukey’s multiple-comparison test.

To investigate whether REGγ promotes the progression and malignancy of KRAS-mutant cancers by cooperating with oncogenic KRAS, we conducted cell viability assays to examine the effects of *REGγ* knockdown on tumor cell growth in KRAS isogenic cell lines. *REGγ* silencing efficiency was confirmed by Western blotting (Supplemental Figure 2A). Our findings revealed that silencing *REGγ* had a more pronounced inhibitory effect on HCT8-*KRAS^G13D^* cells than on HCT8-*KRAS^WT^* cells (Figure 2A). Additionally, we evaluated the response to REGγ depletion across 30 diverse cancer cell lines (Supplemental Table 1). REGγ depletion led to growth arrest in all tested cell lines; however, compared with their wild-type counterparts, cancer cells harboring pan-KRAS mutations exhibited a higher sensitivity to REGγ inhibition (Figure 2B).

To determine whether our in vitro findings could be replicated in vivo, we generated xenograft tumors in an orthotopic cell-derived mouse model by injecting HT29 and HCT116 cells with or without *REGγ* silencing (Figure 2C). Silencing *REGγ* markedly inhibited tumor growth, particularly in the model established with HCT116 cells, which showed unique and higher sensitivity to REGγ depletion (Figure 2D); this was consistent with the results presented in Figure 2, A and B. As expected, we also observed that silencing *REGγ* markedly attenuated the colony-forming capacity of various KRAS-mutant cancer cell lines (Figure 2E). In addition, we explored whether direct compensation of REGγ can, in turn, reverse the inhibition of cancer cell growth observed following *REGγ* knockdown. We concluded that overexpression of REGγ substantially rescued the inhibitory effects of *REGγ* knockdown on cell growth in the A549 and HCT116 cell lines (Figure 2, F and G, and Supplemental Figure 2B). These results indicate the effectiveness and specificity of REGγ inhibition in pan-KRAS–mutant cancer cells.

### NRF2 binds to the REGγ promoter and upregulates REGγ expression in KRAS-mutant cells.

**Figure 3:**
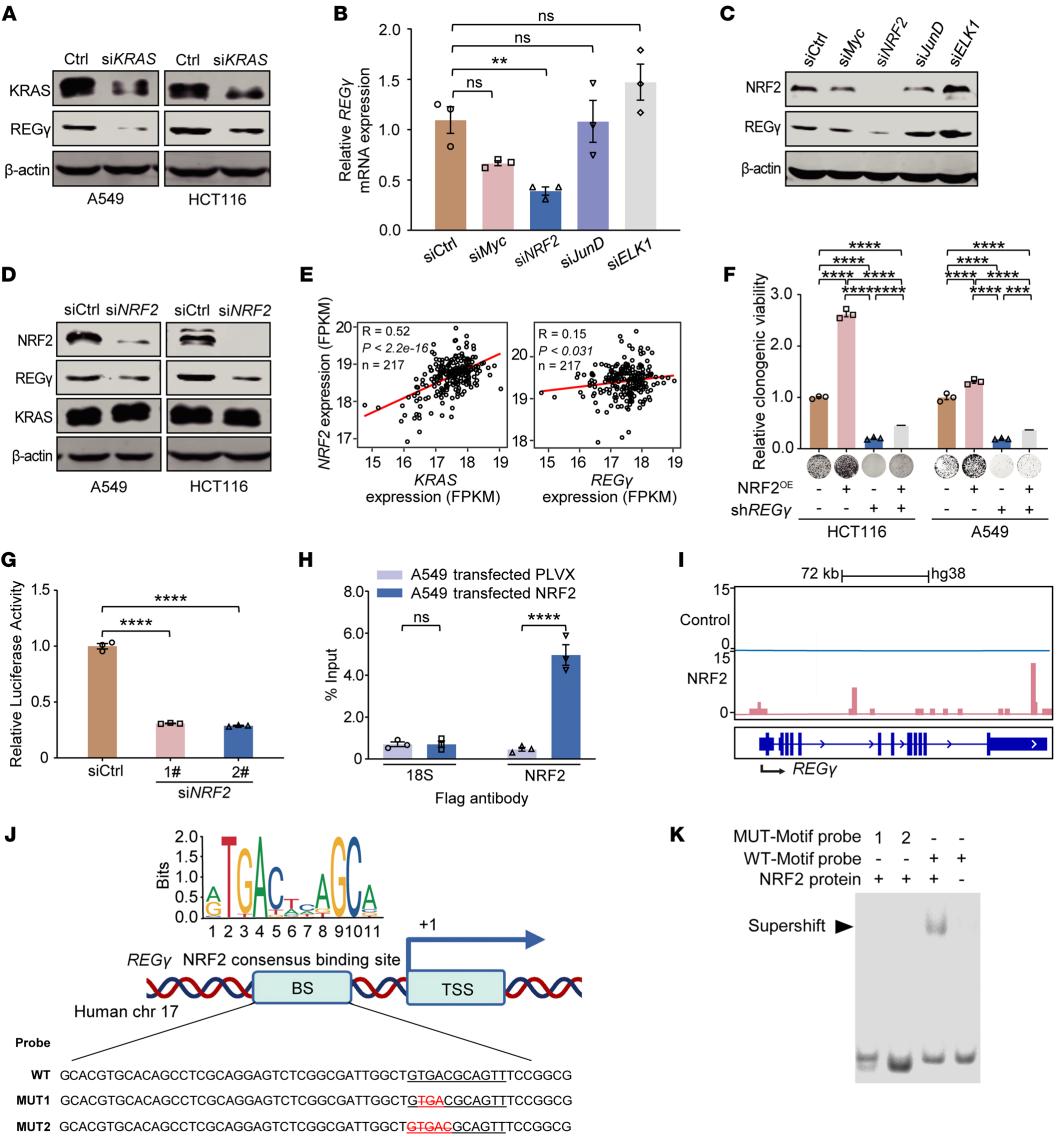
NRF2 binds to the *REGγ* promoter and upregulates its expression in KRAS-mutant cells. (**A**) REGγ expression upon KRAS silencing as determined by Western blot in A549 (*KRAS^G12S^*) and HCT116 (*KRAS^G13D^*) cells. (**B** and **C**) The mRNA (**B**) and protein (**C**) expression of REGγ with knockdown of KRAS downstream transcription factors (Myc, NRF2, JunD, and ELK1) in HCT116 cells. (**D**) Western blot images showing the protein expression of REGγ upon *NRF2* silencing. (**E**) Gene expression information from 217 cases of KRAS-MUT colorectal carcinoma patients in TCGA, reflecting the positive correlation between REGγ or KRAS and NRF2 expression. (**F**) Ectopically expressed NRF2 restored the clonogenic growth of REGγ-depleted cells. The relative viability of cultured colonies in A549 and HCT116 cells is shown. The percentage cell viability is relative to the untreated controls. (**G**) Luciferase reporter assay showing that *NRF2* knockdown decreased the reporter gene expression in A549 cells. Luciferase reporter vectors with promoters contain the indicated *REGγ* promoter regions. (**H**) The interaction between NRF2 and REGγ in A549 cells was verified by ChIP-qPCR assay. NRF2 binding to the *REGγ* promoter. (**I**) UCSC’s Genome Browser tracks showing NRF2 ChIP-Seq signals in the *REGγ* gene locus in HCT116 cells. Blue shading marks the peaks located in the promoter region. (**J**) Top: Schematic showing NRF2 binding sites (JASPAR prediction) on the *REGγ* promoter region. Bottom: The sequences of NRF2 WT probe and mutant probes (MUT1 and MUT2) are shown. (**K**) Human recombinant NRF2 protein was incubated with WT oligonucleotides or MUT oligonucleotides at 37°C for 1 hour, and electrophoretic mobility shift assay was performed. Each value represents mean ± SEM (*n* = 3). **P < 0.01, ***P < 0.001, ****P < 0.0001; *P* values were measured by 1-way ANOVA with Tukey’s multiple-comparison test.

To investigate the regulatory mechanism linking KRAS and REGγ, we separately silenced *KRAS* and *REGγ* in A549 and HCT116 cells. Genetic disruption of *KRAS* expression downregulated REGγ protein expression (Figure 3A). In addition, the silencing of *REGγ* decreased the degradation of its substrate proteins, including Lats1, IκBε, and p21, without affecting KRAS protein expression (Supplemental Figure 3A). These findings suggest that REGγ is a downstream target of KRAS. Since upregulation of REGγ was found to be caused by an increase in transcription (Figure 1H), we next sought to investigate the mechanism underlying the increased transcription of *REGγ*. Myc, NRF2, JunD, and ELK1 have been reported to be critical transcription factors involved in the KRAS signaling pathway (33). Thus, we investigated the effects of these transcription factors on REGγ expression after their silencing. Knockdown of *NRF2* strikingly decreased REGγ mRNA and protein expression in HCT116 cells (Figure 3, B and C). Silencing efficiency was confirmed by real-time PCR (Supplemental Figure 3B). Consistent with these findings, genetic disruption of *NRF2* led to *REGγ* suppression in A549 and HCT116 cells (Figure 3D). Both knockdown of *KRAS* and knockdown of *NRF2* led to an inhibition of REGγ-mediated degradation activity, resulting in the accumulation of REGγ substrates (Supplemental Figure 3C).

To identify the KRAS effectors responsible for NRF2 and REGγ expression, we further used several pharmacological inhibitors to specifically block the main pathways downstream of KRAS, including the MAPK pathway and the PI3K/AKT pathway. Our results illuminated that blockade of key components downstream of KRAS decreased REGγ and NRF2 expression at the transcriptional and translational levels (Supplemental Figure 4, A and B). Analysis of TCGA datasets also revealed that the NRF2 expression level was positively correlated with the KRAS and REGγ expression levels (Figure 3E).

Given that the KEAP1/NRF2 pathway is the conventional pathway for NRF2 degradation (34), we conducted further investigations into KEAP1 expression in *KRAS*-overexpressing isogenic cell lines and *KRAS*-knockdown cell lines, and the results revealed negligible variations in KEAP1 levels, despite notable changes in NRF2 expression (Supplemental Figure 4, C and D). Additionally, analysis of datasets from the Cancer Dependency Map (DepMap) portal did not reveal a substantial correlation between KRAS expression and KEAP1 expression (Supplemental Figure 4E), indicating that NRF2 activation, in the context of KRAS mutations, does not occur via the conventional KEAP1-dependent pathway. Positive correlations were identified between the expression levels of KRAS and REGγ, between the expression levels of KRAS and NRF2, and between the expression levels of REGγ and NRF2 in data from the DepMap database (Supplemental Figure 4F). Furthermore, overexpression of NRF2 in *REGγ*-depleted A549 and HCT116 cells restored the colony-forming capacity of these cells (Figure 3F). Our results revealed that altered NRF2 and REGγ expression at both the mRNA and protein levels occurs via a KEAP1-independent mechanism. Overall, these results suggest that direct genetic disruption of KRAS decreases NRF2 expression and that the signaling pathways downstream of KRAS have the capacity to modulate the NRF2/REGγ axis, indicating a collaborative regulatory mechanism that is crucial for REGγ function.

Next, we sought to determine the mechanism by which NRF2 upregulates REGγ transcription. We performed luciferase reporter assays to investigate the impact of NRF2 on the *REGγ* promoter. Gene silencing of NRF2 specifically repressed *REGγ* promoter activity in A549 cells (Figure 3G). Chromatin immunoprecipitation followed by quantitative PCR (ChIP-qPCR) analysis confirmed the occupancy of NRF2 on the *REGγ* promoter in A549 cells (Figure 3H). Our ChIP-Seq data revealed binding of NRF2 to the promoter of *REGγ* (Figure 3I), which was also confirmed by NRF2 ChIP-Seq data for HeLa-S3 cells from the Gene Expression Omnibus (GEO) DataSets database (GSE91997) (Supplemental Figure 4G). To further investigate the binding between NRF2 and REGγ, we identified an NRF2 binding motif in the *REGγ* promoter using the JASPAR database (Figure 3J). We then performed electrophoretic mobility shift assays, in which a 56 bp probe for the *REGγ* promoter containing the NRF2 binding site was incubated with the purified NRF2 protein. Notably, a clear shift in the DNA-protein complex band was observed in the presence of the wild-type REGγ probe (Figure 3K). In contrast, the introduction of different mutations in the NRF2 binding motif of REGγ substantially reduced the DNA binding activity between the NRF2 protein and *REGγ* DNA (Figure 3K), suggesting the binding specificity of NRF2 for this particular motif within the *REGγ* promoter. Taken together, these findings demonstrate that NRF2 directly binds to the *REGγ* promoter and that this interaction is crucial for increasing *REGγ* transcription.

### Identification of RLY01 as a potent REGγ-20S proteasome inhibitor.

**Figure 4:**
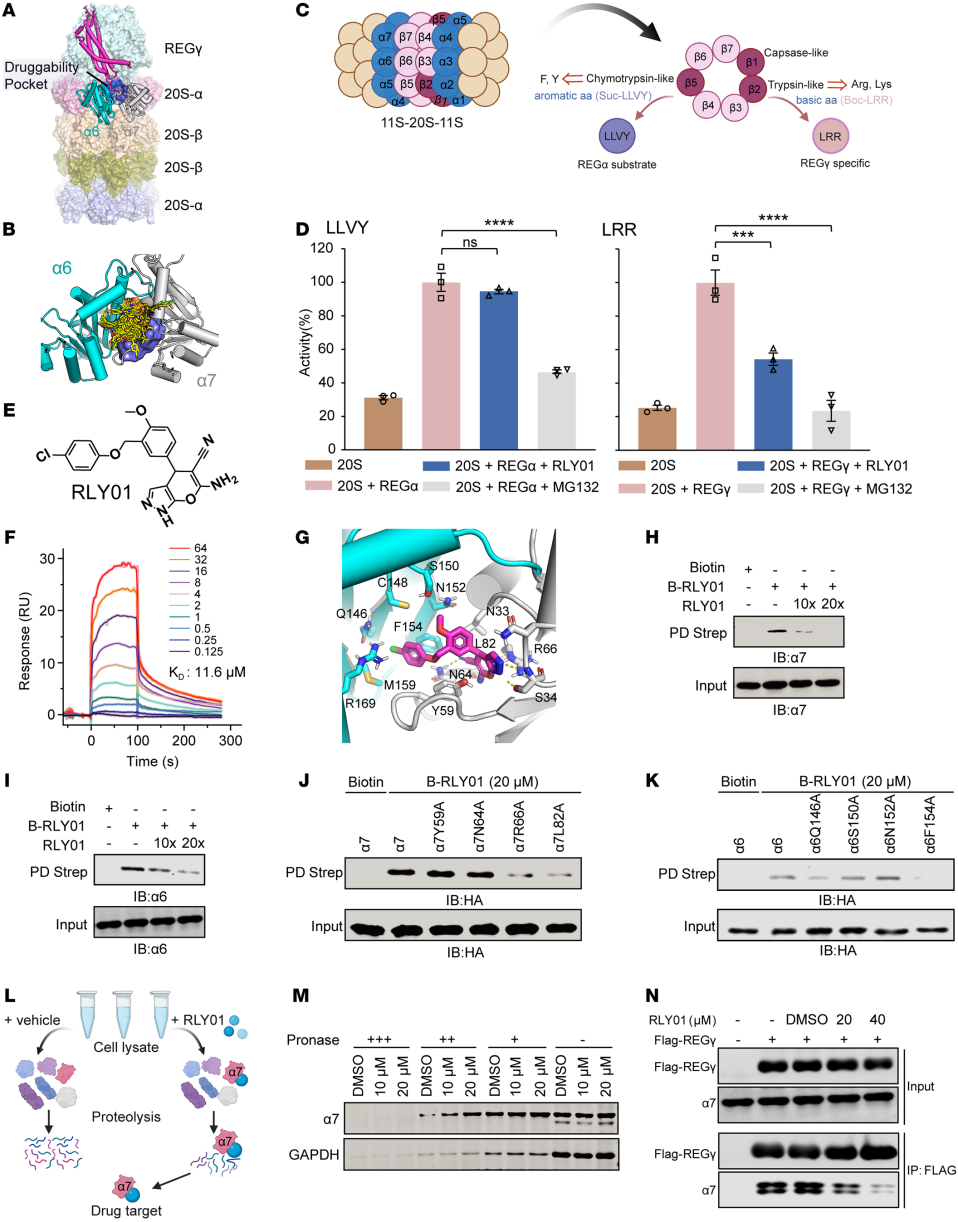
Identification of RLY01 as a potent REGγ-20S proteasome inhibitor. (**A**) A druggability pocket identified on the REGγ-20S complex. (**B**) Docking pose of the top 100 hits within the druggability pocket of 20S α_6_–α_7_ interface. (**C**) Schematic illustration of specific peptidase activity of the 11S proteasome in vitro. (**D**) The inhibition of 11S-activated (REGγ/α) 20S proteasome by RLY01 is peptide substrate-specific. Each value represents mean ± SEM (*n* = 3). ***P < 0.001, ****P < 0.0001; *P* values were measured by 1-way ANOVA with Tukey’s multiple-comparison test. (**E**) Chemical structure of RLY01. (**F**) Surface plasmon resonance sensor-grams and fits for the interaction between RLY01 and 20S proteasome. (**G**) The molecular docking model showing the interaction residues within the α_6_–α_7_ interface pocket of the 20S proteasome to RLY01. (**H** and **I**) Biotin pull-down assay. SW620 cell protein lysates were incubated with biotin, biotin-RLY01 (B-RLY01; 10 μM), B-RLY01 (10 μM) + 100 μM RLY01 (10× molar excess), B-RLY01 (10 μM) + 200 μM RLY01 (20× molar excess). (**J** and **K**) The interaction of 4 mutant variants (Y59A, N64A, R66A, L82A) of α_7_ and 4 mutant variants (Q146A, S150A, N152A, F154A) of α_6_ with RLY01. HA-α_7_, HA-α_6_, and 8 mutant variants were transiently expressed in HCT116 cells for 48 hours. Cell lysates were then incubated in vitro for 12 hours with biotin, 20 μM biotin-RLY01, or DMSO. Protein complexes were subsequently precipitated using streptavidin beads and analyzed by Western blot. (**L**) Scheme of DARTS. (**M**) The DARTS method was used for drug target identification. Immunoblotting shows that RLY01 protected the α_7_ subunit from pronase proteolysis. (**N**) Co-IP showing that RLY01 inhibits the interaction between REGγ and α_7_. FLAG-REGγ was ectopically expressed in HCT116-KO cells for 48 hours, and cell protein lysates were incubated with the indicated treatments for 12 hours in vitro, followed by detection by co-IP and Western blot analysis.

To investigate the therapeutic potential of blocking the activity of REGγ-20S as a targeting strategy in pan-KRAS–mutant cancers, we sought to develop a REGγ-20S proteasome inhibitor by docking-based virtual screening. We determined that the proteasome activator REGγ but not PA26 or REGα strongly interacted with only the α_7_ subunit of the 20S proteasome and not with the other subunits (α_1_–α_6_) via a coimmunoprecipitation (co-IP) assay in 293T cells (Supplemental Figure 5, A and B) and a yeast 2-hybrid assay (Supplemental Figure 5C). The recently reported structure of the REGγ-20S complex also shows that the C-terminal region of REGγ enters and binds to residues in the α_6_–α_7_ interface pocket of the 20S proteasome (22). Through site-directed mutagenesis studies focused on the binding pocket, we identified 2 residues (α7N33 and α7S34) that, when mutated to alanine, notably reduced the interaction between 20S and REGγ (Supplemental Figure 5D), indicating that this pocket is important for REGγ-dependent 20S proteasome activity. A small molecule that binds to this pocket may also prevent the REGγ-20S interaction and inhibit proteasome activity (Figure 4A).

Thus, on the basis of this pocket (Figure 4B), a structure-based virtual screen of the 220,000 compounds in the SPECS library (https://www.specs.net) was carried out. We selected 100 candidate compounds with diverse chemical scaffolds from among the top 2,000 candidate compounds with the highest docking scores (Supplemental Figure 6A) and then devised a screening pipeline to assess their binding efficacy. Initially, the ability of the 100 selected compounds to increase the protein stability of p21, which is degraded in a REGγ-dependent manner, in HeLa cells at an initial concentration of 30 μM was evaluated. The top 2 compounds (compounds 85 and 98) were considered hits (Supplemental Figure 6, B and C) and subsequently subjected to a rescreening process using a cell-free proteolysis assay to determine whether they could selectively interfere with peptidase activity stimulated directly by REGγ (Figure 4C). Compound 85, renamed RLY01, was found to partially inhibit the activation of Boc-LRR-AMC (LRR) degradation, whereas the degradation of Suc-LLVY-AMC (LLVY) was essentially insensitive to the presence of RLY01 (Figure 4D). On the other hand, compound 98 had suppressive effects on the degradation of both LRR and LLVY, similarly to the broad-spectrum proteasome inhibitor MG132 (Figure 4D and Supplemental Figure 6D). Ultimately, RLY01 (Figure 4E) emerged as the top candidate owing to its specific effects and ability to inhibit REGγ-dependent proteasome substrate hydrolysis in vitro. Consistent with the docking model, the results of surface plasmon resonance analysis revealed an affinity (*K_D_*) of 11.6 μM, which supported the potential interaction between RLY01 and the 20S proteasome (Figure 4F).

Computational modeling suggested that RLY01 binds tightly to a cleavage pocket in the α_6_–α_7_ interface of the 20S proteasome, thereby blocking REGγ-20S proteasome complex formation. The small molecule RLY01 interacts with the Q146, C148, S150, N152, F154, M159, and R169 residues in the 20S α_6_ subunit and the N33, S34, Y59, N64, R66, and L82 residues in the α_7_ subunit (Figure 4G). Moreover, RLY01 interacts with α_7_ residues that are known to be involved in the binding of REGγ, such as N33 and S34. To further study the binding of RLY01 to the pocket of the 20S proteasome, we synthesized a biotinylated derivative of RLY01 (biotin-RLY01). Excess RLY01 competed with α_6_ and α_7_ of the 20S proteasome for binding to biotin-RLY01, indicating the specific binding of RLY01 and the potential for using RLY01 as a competitor (Figure 4, H and I). Interestingly, the amino acid mutations α7R66A, α7L82A, α6Q146A, and α6F154A directly abolished the interaction between RLY01 and 20S-α_7_ or 20S-α_6_ in the biotin pulldown assay, further highlighting the potent ability of RLY01 binding to suppress REGγ-20S proteasome activity (Figure 4, J and K). Additionally, a drug affinity responsive target stability (DARTS) assay was performed to identify the protein(s) that directly bind the drug (Figure 4L). Our results revealed that RLY01 protected and enriched α_7_ during proteolysis (Figure 4M). RLY01 dramatically pulled down endogenous α_7_ and decreased the interaction between REGγ and α_7_ in a concentration-dependent manner, as verified by co-IP (Figure 4N and Supplemental Figure 6E). Furthermore, we conducted biotin-RLY01 pulldown mass spectrometry analysis to examine protein binding to RLY01. As shown in Supplemental Figure 6F, the silver staining results revealed additional bands in the lysates of cells incubated with biotin-RLY01 in comparison with those in the controls. Subsequent mass spectrometry analysis demonstrated a strong association, as RLY01 effectively precipitated α_7_ (Supplemental Figure 6G and Supplemental Table 2). These data suggest that RLY01 binds to and occupies the REGγ-bound pocket, suppressing REGγ-dependent 20S proteasome activity.

To determine the ability of RLY01 to inhibit REGγ-20S proteasome activity in vivo, we also assessed its pharmacokinetic properties in mice. RLY01 was detected in the plasma of BALB/c mice 24 hours after the administration of 20 mg/kg RLY01, with a maximum concentration of 6,440 ng/mL and a half-life of 4.14 hours (Supplemental Figure 6H). These pharmacokinetic properties indicated the suitability of RLY01 for exploratory in vivo evaluation.

### RLY01 blocks the degradation function of the REGγ-20S proteasome in a REGγ-dependent manner.

**Figure 5:**
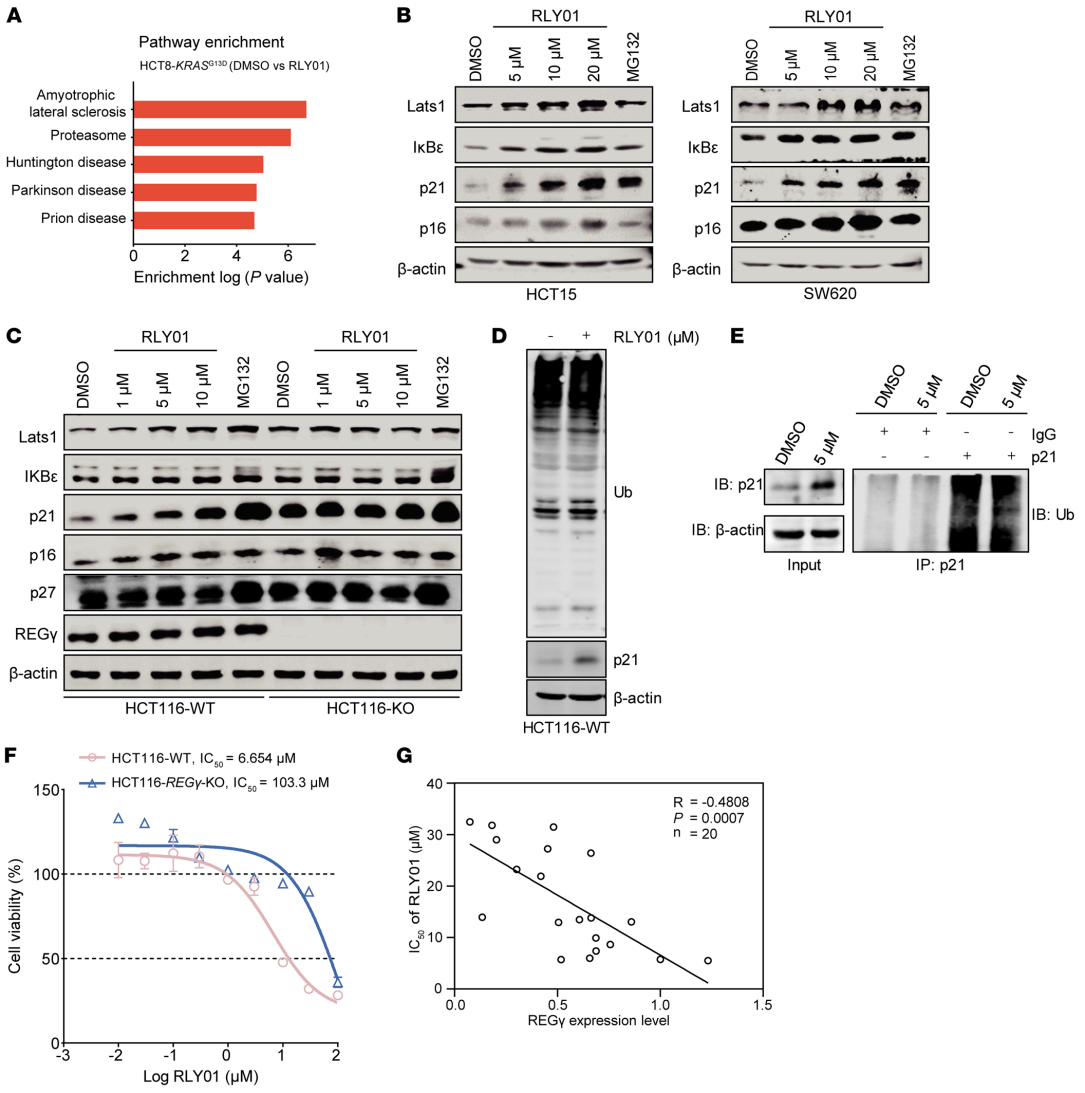
RLY01 blocks REGγ-20S proteasome degradation functions in a REGγ-dependent manner. (**A**) Pathway enrichment in HCT8-*KRAS^G13D^* cells after DMSO or RLY01 (10 μM) treatment in proteomic profiling. (**B**) RLY01 treatment for 12 hours promotes accumulation of the REGγ-proteasome substrates Lats1, IκBε, p21, and p16 in HCT15 and SW620 cells. MG132 (5 μM) is a positive control. β-Actin is a control for protein loading. Representative blots are shown from 3 independent experiments. (**C**) RLY01 promotes accumulation of the REGγ targets Lats1, IκBε, p21, and p16 in a REGγ-dependent manner in HCT116 cells rather than HCT116-KO cells. MG132 (5 μM) is a positive control. β-Actin is a control for protein loading. Representative blots are shown from 3 independent experiments. (**D**) HCT116 cells were treated with RLY01 (5 μM) for 8 hours, and then ubiquitination levels were detected. (**E**) HCT116 cells were treated with RLY01 (5 μM) for 8 hours, then lysed with IP lysis/wash buffer with protease inhibitor and phosphatase inhibitor. p21 was immunoprecipitated with an anti-p21 antibody, and the immune precipitates were probed with anti-ubiquitin, anti-p21, and anti–β-actin antibodies. (**F**) Inhibitory effects of RLY01 on HCT116 isogenic cell lines. (**G**) IC_50_ of RLY01 in various cancer cells is negatively correlated with REGγ expression.

To further confirm and validate the on-target specificity of RLY01, proteomic profiling was conducted to characterize the pathway alterations induced by RLY01 treatment or *REGγ* knockdown in HCT116 cell lines. Both *REGγ* knockdown and RLY01 treatment predominantly affected the proteasome pathway, which was among the top results in the KEGG pathway enrichment analysis (Figure 5A, Supplemental Figure 7A, and Supplemental Table 3). To eliminate the possibility that an increase in p21 protein expression occurred due to cell growth arrest and senescence (35, 36), we examined the accumulation of other REGγ targets, including Lats1, IκBε, and p16, in various cancer cell lines. Interestingly, RLY01 increased the levels of REGγ-proteasome substrates in a dose-dependent manner in HCT15, SW620, HCT116-WT, and SW480 cells (Figure 5, B and C, and Supplemental Figure 7B). Additionally, RLY01 did not lead to increases in the levels of REGγ-proteasome substrates in HCT116-*REGγ*-KO (HCT116-KO) cells, but it did cause the accumulation of these proteins in HCT116-WT cells (Figure 5C). In addition, RLY01 did not cause changes in the protein level of p27, which is regulated by the ubiquitin-dependent proteasome degradation pathway (Figure 5C). RLY01 increased the levels of the REGγ substrate p21, but it did not affect ubiquitination levels in HCT116-WT cells (Figure 5D). To this end, we treated the indicated cells with RLY01 (5 μM) for 8 hours and performed immunoprecipitation analysis with an anti-p21 antibody. We found that RLY01 did not alter the polyubiquitination level of p21 (Figure 5E). These results indicate that RLY01 inhibits the function of the REGγ-20S proteasome without affecting its level of ubiquitination. Consistent with the effects observed following genetic deletion of REGγ, RLY01 had obviously higher IC_50_ values in HCT116-KO cells than in HCT116-WT cells, indicating a REGγ-dependent antiproliferative activity (Figure 5F). The IC_50_ values for RLY01 across various cell lines demonstrated an inverse relationship with the level of REGγ expression (Figure 5G and Supplemental Table 4). These results suggest that RLY01 is a specific inhibitor of the REGγ-20S proteasome and interferes with the biological ability of REGγ to activate the 20S proteasome in a REGγ- and dose-dependent manner.

### Pharmacological inhibition of REGγ blocks the growth of pan-KRAS–mutant tumors in vitro.

**Figure 6:**
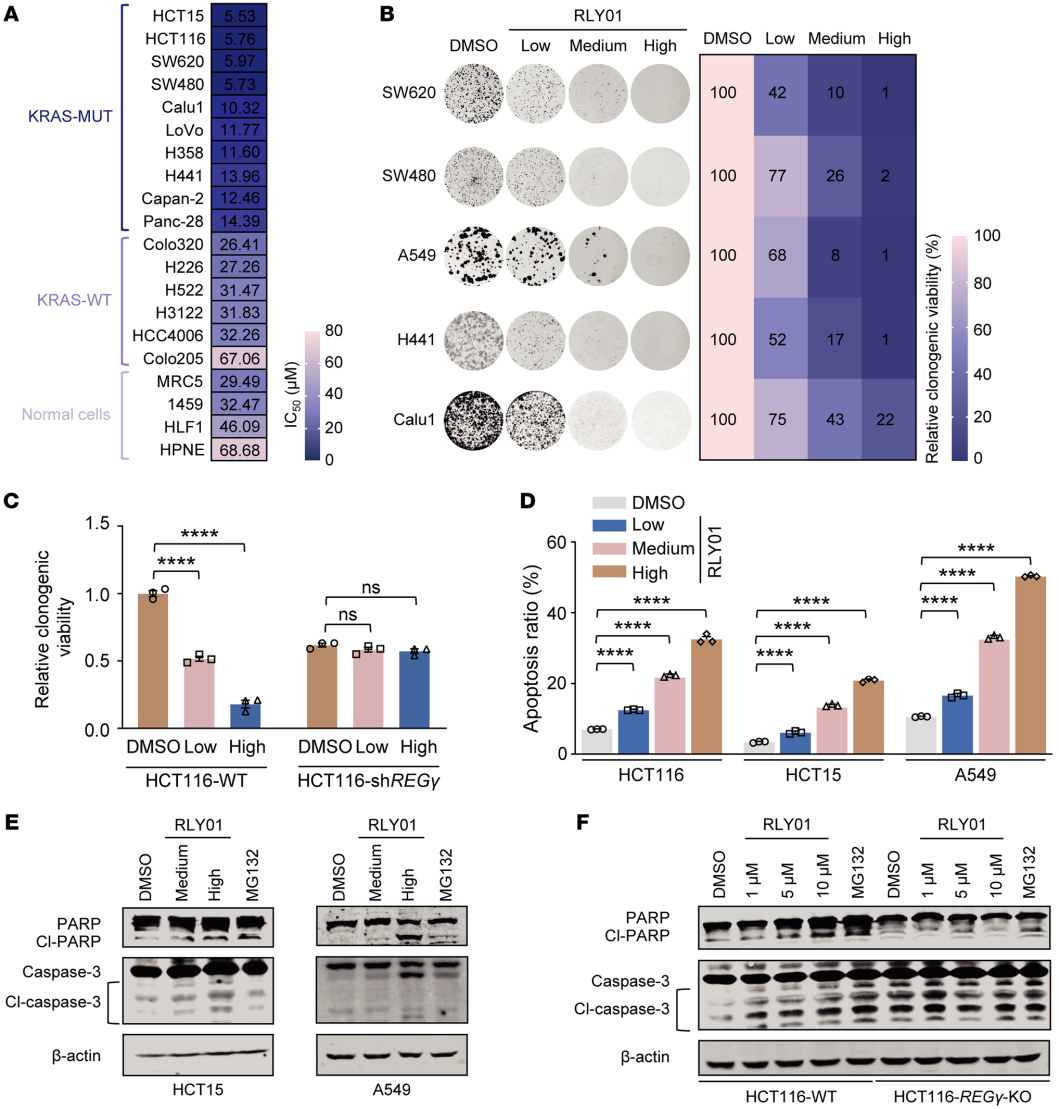
Pharmacologic inhibition of REGγ blocks the growth of pan-KRAS–mutant tumors in vitro. (**A**) Ten KRAS-MUT and 6 KRAS-WT cancer cell lines and 4 normal cell lines were treated with the indicated concentrations of RLY01 for 72 hours. (**B**) The colony-forming ability of indicated cell lines after treatment with RLY01 at their respective one-quarter IC_50_ (Low), one-half IC_50_ (Medium), or IC_50_ (High). The relative viability of cultured colonies was calculated by normalization of the untreated group as 100%. (**C**) Relative clonogenic viability after treatment with RLY01 at their respective one-quarter IC_50_ (Low) or IC_50_ (High) in HCT116-WT and HCT116-sh*REGγ* cells. Each value represents mean ± SEM (*n* = 3). ****P < 0.0001; *P* values were measured by 2-way ANOVA with Tukey’s multiple-comparison test. (**D**) HCT116, HCT15, and A549 cells were treated with RLY01 for 48 hours, and the percentage of apoptotic cells was determined by annexin V and propidium iodide staining. Each value represents mean ± SEM (*n* = 3). ****P < 0.0001; *P* values were measured by 2-way ANOVA with Tukey’s multiple-comparison test. (**E** and **F**) Immunoblotting showing that the apoptosis-related proteins cleaved PARP (Cl-PARP) and cleaved caspase-3 (Cl-caspase-3) were upregulated after RLY01 treatment in HCT15, A549 (**E**), and HCT116 (**F**) cells, whereas there was no obvious change in HCT116-KO cells (**F**).

To test the association between pharmacological inhibition of REGγ and the KRAS mutation status, we treated KRAS-MUT, KRAS-WT, and normal cell lines (Supplemental Table 5) with RLY01. Intriguingly, RLY01 selectively killed KRAS-mutant cells, with much lower IC_50_ values than observed in KRAS-WT cells, normal human fibroblasts, or epithelial cells (Figure 6A).

Next, we analyzed the clonogenic growth of SW620, SW480, A549, H441, and Calu1 cells after exposure to different concentrations of RLY01. Our findings demonstrated that RLY01 effectively suppressed the clonogenic growth of KRAS-mutant cancer cells (Figure 6B). Furthermore, the clonogenic growth suppression mediated by RLY01 was reversed by *REGγ* silencing in HCT116-sh*REGγ* cells (Figure 6C and Supplemental Figure 8A). Additionally, RLY01 markedly induced apoptosis in KRAS-mutant cells, with a concentration-dependent increase in the number of apoptotic cells compared with that in the untreated control group (Figure 6D and Supplemental Figure 8B).

Moreover, RLY01 increased the levels of the apoptosis-related proteins cleaved PARP (Cl-PARP) and cleaved caspase-3 (Cl-caspase-3) in KRAS-mutant cells in a concentration-dependent and REGγ-dependent manner (Figure 6, E and F). In contrast, RLY01 had no substantial effect on apoptosis in HCT116-*REGγ*-KO cells (Figure 6F). Collectively, these results suggest that RLY01 effectively induces apoptosis and inhibits the growth of pan-KRAS–mutant cancer cells from various tissues by blocking the function of the REGγ-20S proteasome.

### The in vivo therapeutic effect of RLY01 on KRAS-mutant tumors.

**Figure 7:**
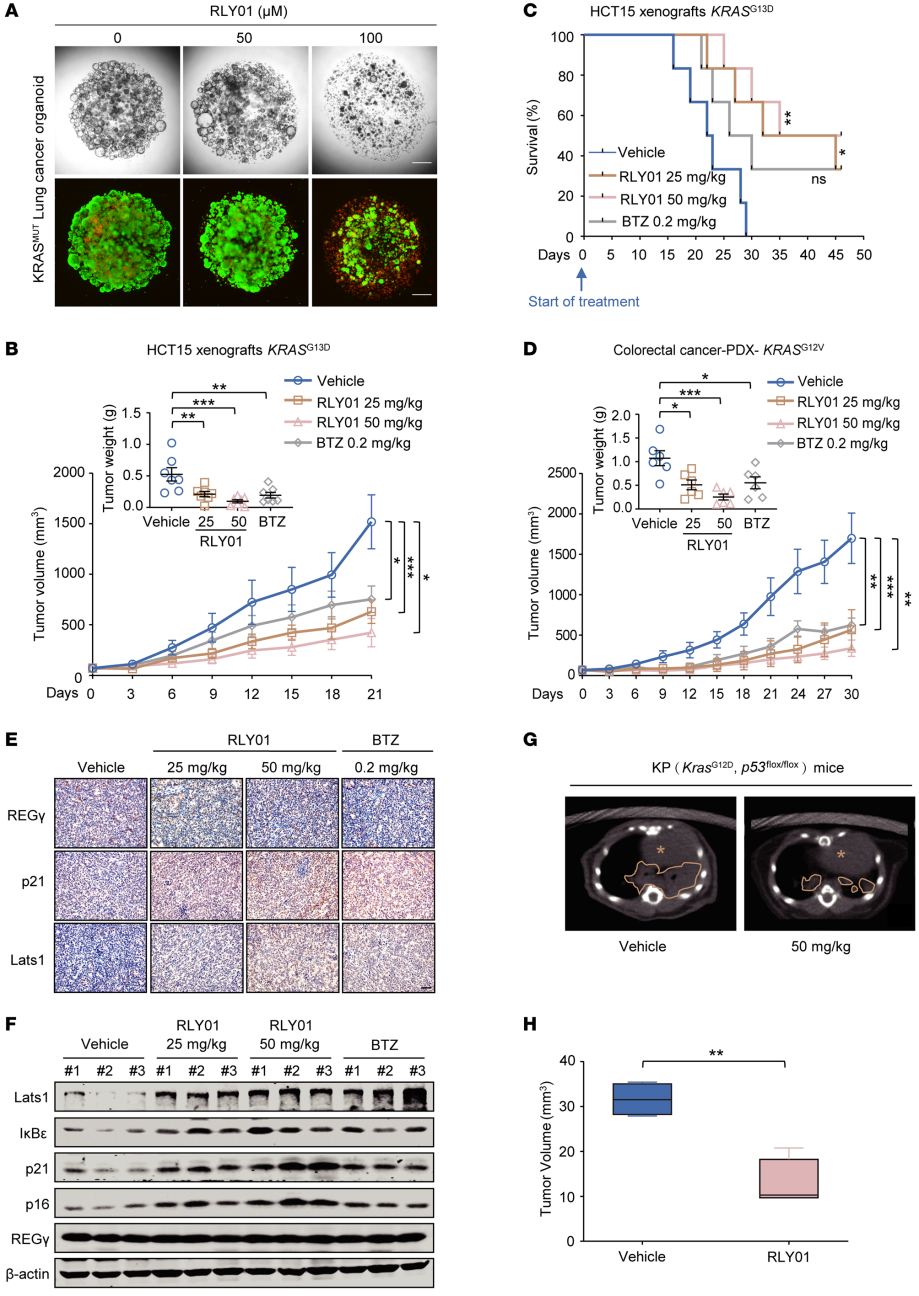
The in vivo therapeutic effect of RLY01 in KRAS-mutant tumor suppression. (**A**) Top: Response of organoids derived from *KRAS^G12F^* lung cancer to RLY01. Bottom: Green fluorescence represents calcein AM staining for live cells, while red fluorescence represents propidium iodide staining for dead cells. Representative images from 3 technical replicates with similar results. Scale bars: 500 μm. (**B**) HCT15 xenograft growth curve (*n* = 6). Mean weights of tumors on day 21 are shown in the inset. (**C**) Kaplan-Meier survival curves of HCT15 xenograft model mice (*n* = 6–7). (**D**) Colorectal patient xenograft growth curve (*n* = 7). Mean tumor weight on day 30 is shown in the inset. (**E**) Representative IHC images of expression of the REGγ targets Lats1 and p21\ Scale bar: 50 μm. (**F**) Immunoblotting for protein levels of REGγ and REGγ-proteasome substrates with or without RLY01 therapy by peritoneal injection in PDX. Representative blots are shown from 3 independent experiments. (**G**) Representative images of tumors from LSL-*Kras^G12D^* *Trp53^fl/fl^* mice. Animals were scanned by micro-CT. Yellow lines indicate areas with lung tumors, and yellow asterisks indicate heart. (**H**) Box plots showing the tumor volumes at the endpoint of the indicated treatments based on micro-CT (*n* = 4). The horizontal lines represent the median; the bottom and top of the boxes represent the 25th and 75th percentiles, respectively. The vertical bars represent the range of the data. All data are shown as mean ± SEM. *P < 0.05, **P < 0.01, ***P < 0.001. *P* values were measured by 1-way ANOVA with Tukey’s multiple-comparison test.

To gain a better understanding of RLY01 and develop a treatment strategy based on its use, we generated human lung cancer organoids as a disease model and conducted drug testing to assess the therapeutic potential of RLY01. RLY01 strikingly suppressed organoid growth at the tested concentrations (Figure 7A). In parallel, we generated a series of preclinical mouse models to evaluate the therapeutic efficacy of RLY01 against KRAS-mutant tumors in vivo. First, we established a colorectal cancer xenograft mouse model via the subcutaneous injection of HCT15 cells (*KRAS^G13D^*) into the flanks of male athymic nude mice aged 4–5 weeks. When the tumor volume was approximately 70 mm^3^, the mice were treated with vehicle or different doses of RLY01 daily via intraperitoneal injection (Supplemental Figure 9A). As shown in Figure 7B and Supplemental Figure 9B, continuous treatment with RLY01 at all the tested doses substantially inhibited tumor growth. In the control group, the mice injected with HCT15 cells began to die on day 16, with a median survival time of 22.5 days. Conversely, treatment with RLY01 resulted in a notable long-term survival advantage, with median survival times of 38.5 days in the 25 mg/kg group and 40.5 days in the 50 mg/kg group (Figure 7C). Importantly, RLY01 treatment was well tolerated, as no systematic toxicity was observed in any group during these experiments (Supplemental Figure 9, C–F). To assess the translational efficacy of RLY01, we further employed a patient-derived xenograft (PDX) model of colorectal carcinoma harboring a G12V mutation in *KRAS* (Supplemental Figure 9G). After 4 weeks of treatment, RLY01 treatment markedly suppressed PDX tumor growth (Figure 7D and Supplemental Figure 9H). Moreover, immunohistochemical staining and Western blot analysis revealed that RLY01 treatment led to the accumulation of REGγ-proteasome substrates, such as Lats1, p21, IκBε, and p16 (Figure 7, E and F). Consistent with the results shown in Supplemental Figure 9C, RLY01 had no toxic effects on PDX tumor–bearing mice at the tested doses, as determined on the basis of body weight (Supplemental Figure 9I).

Next, we established a *Kras^G12D^* *Trp53^fl/fl^* (KP) mouse lung cancer model (Supplemental Figure 9J). In the vehicle group, lung adenocarcinoma tumors exhibited aggressive growth and rapid spreading throughout the lung tissue over a 1-month period. Encouragingly, compared with vehicle treatment, RLY01 treatment inhibited tumor growth (Figure 7, G and H).

To investigate whether RLY01 can overcome the drug resistance induced by AMG510 treatment, we established drug-resistant cells with secondary KRAS mutations that confer resistance to AMG510. We introduced *KRAS* G12C plus 1 of 2 secondary mutations (G13D or Y96D) into Calu1 and H358 cells. In the growth inhibition assay, the IC_50_ values of AMG510 in Calu1 cells harboring the G12C plus G13D or G12C plus Y96D mutations were approximately 5–6 times greater than those in the parental Calu1 cells (Supplemental Figure 10B). However, there were no obvious differences in the IC_50_ values after treatment with RLY01 (Supplemental Figure 10A). Remarkably, our results revealed that RLY01 dramatically enhanced the antigrowth effect on AMG510-resistant Calu1 cells harboring the G12C plus G13D or G12C plus Y96D mutations (Supplemental Figure 10, C and D), with all combination index (CI) values less than 0.65, indicating a synergistic effect of RLY01 and AMG510. Besides, we observed a similar synergistic effect in a long-term colony formation assay (Supplemental Figure 10, E–H). These data indicate that the combination of RLY01 and AMG510 leads to more effective inhibition of growth in AMG510-resistant cells, implying that blockade of the biological function of REGγ by RLY01 may enhance sensitivity to AMG510 treatment in cancers with acquired resistance to *KRAS^G12C^* inhibitors. On the basis of the results from the mouse models, we confirmed that RLY01 exhibited potent in vivo efficacy with satisfactory results when it was administered as either a monotherapy or combination therapy. Our findings identify a promising treatment option for pan-KRAS–mutant lung and colorectal carcinomas.

## Discussion

The KRAS oncogene has attracted notable attention because of its frequent mutation and its role in initiating and sustaining tumor growth (37, 38). However, the efficacy of KRAS-selective drugs varies because of the heterogeneity of KRAS mutations (5, 39). Consequently, there is a pressing need for universal treatment strategies targeting common KRAS mutations. Here, we highlighted the elevated proteasome activity in KRAS-mutant tumors and identified REGγ, a proteasome activator that serves as a susceptibility factor downstream of KRAS.

In this study, we used proteomic analysis to identify KRAS-associated vulnerability factors in cancer cells, revealing that REGγ is a key driver of growth and survival in KRAS-mutant cancers. Blockade of REGγ markedly impaired cell proliferation and survival in KRAS-mutant tumors both in vitro and in vivo, highlighting that REGγ is a promising and translatable target with functional implications. REGγ plays a critical role in tumorigenesis, as it promotes cell proliferation and metastasis while simultaneously inhibiting apoptosis (31). The tumor-specific regulatory network of the REGγ-20S proteasome involves complex interactions with multiple pathways (40–43). Intriguingly, we found that NRF2, a transcriptional activator, upregulates REGγ expression in KRAS-mutant cancers. NRF2, which is known as the master regulator of stress response genes, is exploited by cancer cells to create a prosurvival microenvironment that supports drug resistance (44–46). However, the details of the mechanism by which NRF2 mediates KRAS-driven drug resistance are still poorly understood. Here, we found that upregulation of REGγ and the abnormal activation of the REGγ-proteasome pathway are potential contributors to drug resistance. Our study unveiled a regulatory mechanism, the KRAS/NRF2/REGγ axis, shedding new light on KRAS-driven drug resistance.

Previous studies have shown that KRAS mutations enhance proteasome capacity by increasing the expression of proteasome subunits (47). Combined treatment with the proteasome inhibitor bortezomib (BTZ) and MAPK inhibitors was shown to exhibit enhanced antitumor activity in RAS-activated multiple myeloma models (47). However, BTZ and other proteasome inhibitors have not yet demonstrated consistent antitumor activity against solid tumors in the clinical setting (48). In addition, multiple clinical studies of BTZ have reported increased rates of certain toxicities, notably peripheral neuropathy, gastrointestinal issues, thrombocytopenia, and herpes zoster reactivation (49, 50). Here, we discovered a class of proteasome inhibitors exemplified by RLY01 through structure-based molecular design. Ubiquitin-independent proteasome inhibition shares some mechanistic similarities with 26S proteasome inhibition, but also presents profound differences. For example, RLY01 binds to the pocket at the α_6_–α_7_ interface of the 20S proteasome, specifically preventing REGγ from binding and thus blocking the degradation of REGγ-dependent protein substrates (Figures 4 and 5). RLY01 exerted potent antitumor effects on KRAS-driven cancers by modulating cell proliferation and apoptosis both in vitro and in vivo. Furthermore, RLY01 enhanced the sensitivity of KRAS-mutant tumor cells to AMG510, thereby overcoming resistance to *KRAS^G12C^* inhibitors (Supplemental Figure 10). These findings provide a scientific rationale for evaluating REGγ-20S proteasome inhibitors as potential therapeutic options for pan-KRAS–mutant cancers. In our in vivo experiments, RLY01 demonstrated good tolerability, with no gross toxicity in tumor-bearing mice (Supplemental Figure 9, E–J). Considering the high prevalence of KRAS point mutations in human tumors and the responsive role of REGγ in KRAS oncogenic signaling, inhibitors targeting the REGγ-proteasome complex could enhance antitumor activity and represent a broadly applicable approach in cancer therapy. More excitingly, the development of both direct and indirect pan-KRAS inhibitors could pave the way for treating tumors harboring all types of KRAS mutations.

This study of RLY01 has several limitations. Further studies should prioritize verifying the compound’s stability for in vivo applications, as well as investigating its pharmacokinetic profile, solubility, and chemical stability. Combination treatment–based anticancer therapeutic approaches using RLY01 also require further evaluation.

In summary, this study identifies REGγ as a KRAS-associated vulnerability that promotes pan-KRAS–mutant cancer progression. Through a screening experiment, we identified an innovative REGγ-20S proteasome inhibitor, RLY01, that contributes to the treatment of KRAS-mutant cancers. Our findings suggest that a developed REGγ-specific inhibitor could be a pivotal therapeutic option for patients with heterogeneous types of pan-KRAS–mutant tumors.

## Methods

### Sex as a biological variable.

For clinical samples and animal models, both sexes were involved. The findings were expected to be relevant to both sexes.

### Cells.

A549, HCT116, HT29, HCT8, Calu1, LoVo, LS174T, COLO205, COLO320, RKO, Panc-28, Capan-2, PC3, 5637, T24, HPNE, 1459, WI38, E6E7, IMR90, HLF1, HEK293T, and MEF cells were cultured in complete Dulbecco’s modified Eagle medium supplemented with 10% FBS (Sunrise, SR100180.03) and 1% penicillin/streptomycin (Aqlabtech, AQ512). HCT15, SW620, SW480, H358, H441, H460, H226, H3122, HCC4006, H522, H838, H661, H1299, A427, H23, H647, H727, H1944, H1975, and MRC5 cells were cultured in complete Roswell Park Memorial Institute medium supplemented with 10% FBS and 1% penicillin/streptomycin.

HCT8, HCT116, A549, and H838 stable-overexpression cells were generated by molecular cloning in pLVX-Puro vector. HCT116, HT29, and H1299 stable-knockdown cells were generated by shRNAs (Supplemental Table 6) in pLKO.1 vector.

### Mice.

NOD/shiltjnju strain NCG mice, BALB/c nude mice, and C57BL/6 mice were purchased from the specific pathogen free–level (SPF-level) scientific research animal center of East China Normal University (animal ethical number 2024-DWYY-103).

### Patient samples.

All tumor specimens were provided by Fudan University Shanghai Cancer Center.

PDX model (human *KRAS^G12V^*-mutant colon cancer xenograft tumor model) was purchased from BEIJING IDMO Co. Ltd. (medical science ethical number 2024-081).

### Tandem mass tag labeling liquid chromatography–tandem mass spectrometry statistical analyses.

The RAW data files were analyzed using Proteome Discoverer (Thermo Fisher Scientific, version 2.2). The tandem mass spectrometry (MS/MS) search criteria were as follows: mass tolerance of 10 ppm for MS and 0.02 Da for MS/MS, trypsin as the enzyme with 2 missed cleavages allowed, carbamidomethylation of cysteine and the tandem mass tag (TMT) of N-terminus and lysine side chains of peptides as fixed modifications, and methionine oxidation as dynamic modification. False discovery rate (FDR) of peptide identification was set as ≤0.01. A minimum of 1 unique peptide identification was used to support protein identification. The thresholds of fold change greater than 1.5 and *P* value less than 0.05 were used to identify differentially expressed proteins. Then we identified 140 upregulated proteins and 434 downregulated proteins in HCT8-*KRAS^G13D^* compared with HCT8-*KRAS^WT^* cells. The results are shown in Supplemental Tables 7 and 8.

### Knockdown via small interfering RNA.

Cells were seeded in a 6-well plate at a density of 80%. After cell adhesion, cells were transfected for 48 hours with small interfering RNA (siRNA) using the lipo8000 transfection reagent according to the manufacturer’s instructions (Beyotime, C0533). The cells were lysed for Western blotting to determine the knockdown efficiency. Targeting sequences for siRNAs are summarized in Supplemental Table 6.

### Plasmid construction and stable transfection.

Plasmids used in this study are summarized in Supplemental Table 9. Target full-length *KRAS*, FLAG-*REGγ*, HA-*α7*, or *NRF2* cDNA was cloned into a pcDNA, PSG5, or pLVX vector using ClonExpress II One Step Cloning Kit (Vazyme, C112-01) and corresponding primers (Supplemental Table 8). siRNA sequence was cloned into pLKO.1 plasmid vector as short hairpin RNA (shRNA).

HEK293T cells were cotransfected with lentiviral plasmid DNA, pMD2.G, and psPAX2 for 48 hours. Then culture medium containing lentivirus was collected and filtered. Target cells were infected by virus for 12 hours and further selected with puromycin or blasticidin. Selected cells were verified by Western blotting and then used for further experiments.

### Site-directed mutagenesis.

All KRAS (G12C, G12D, G12V, G12S, G13D, Y96D) mutations and α_7_ (N33A, S34A, Y59A, N64A, R66A, L82A) mutations were constructed from the original overexpression plasmid by the site-directed mutagenesis primers listed in Supplemental Table 8. PCR product obtained by 2× Phanta Max Master Mix (Vazyme, P515-01) was digested with FastDigest DpnI (Thermo Fisher Scientific, FD1704) for 30 minutes at 37°C.

### RNA preparation and real-time PCR.

Total RNA was extracted using the RNA isolator Total RNA Extraction Reagent (Takara, T9109) according to the manufacturer’s protocol. RNA was transcribed into cDNA using the 5× HiScript II qRT SuperMix (Vazyme, R222-01). Real-time PCR was performed using the SYBR qPCR Mix (TOYOBO, QPK-201). Gene expression levels were calculated based on the 2^–ΔΔCt^ relative quantification method. The primers used in this study are shown in Supplemental Table 8.

### Western blotting.

Protein extracts were loaded onto SDS polyacrylamide gels. Gels were transferred onto nitrocellulose membranes, and the membranes were blocked for 1 hour. After overnight incubation with primary antibody at 4°C, the membranes were washed and probed with fluorescent IRDye 800 CW goat anti-rabbit IgG (1:10,000), IRDye 800 CW goat anti-mouse (1:10,000), and IRDye 680 RD goat anti-mouse IgG (1:5,000) antibodies (Cell Signaling Technology). The membranes were then imaged with the Odyssey Infrared Imaging System (LI-COR Biosciences). The primary antibodies used were mouse anti–β-actin (MBL, M177-3), rabbit anti-REGγ (Abcam, ab157157), rabbit anti-KRAS (Abcam, ab275876), rabbit anti-NRF2 (Abcam, ab137550), mouse anti-GAPDH (Proteintech, 60004-1-Ig), anti-FLAG tag (Solarbio, K200001M), anti-HA tag (Abcam, ab9110), rabbit anti-α_7_ (Abcam, ab133502), rabbit anti-LATS1 (Cell Signaling Technology, 3477), rabbit anti-IκBε (Abcam, ab13413), rabbit anti-p16 (Proteintech, 10883-1-AP), mouse anti-p21 (BD Biosciences, 612234), rabbit anti-PARP (Cell Signaling Technology, 9542), and rabbit anti–caspase-3 (Proteintech, 9662).

### Cell viability assay.

Cells were seeded into 96-well plates at a density of 1,500 cells per well. Cells were then cultivated at 37°C for different periods (0 hours, 24 hours, 48 hours, 72 hours, 96 hours). Upon measurement, 10 μL CCK-8 (Yeasen, 40203ES80) mixed with 100 μL serum-free medium was added to each well and incubated for 1 hour at 37°C. Plates were shaken, and optical density values were determined at 450 nm using a microplate reader (BioTek, Synergy Neo2).

Combination index was calculated using Compusyn software version 1.0, and the synergistic effects were determined by the Chou-Talalay method (51).

### Colony formation assay.

Cells were equally seeded into 12-well plates at a density of 2,000 cells per well. Cultivated for 6–10 days, cells were fixed with 4% polyoxymethylene and stained with 0.2% crystal violet. Images of stained colonies were taken, and the numbers of the colonies were recorded and compared within different groups. Each experiment was representative of at least 3 independent experiments.

### Flow cytometry.

The Apoptosis Detection Kit (MultiSciences Biotech Co. Ltd.) was used to detect cell death according to the manufacturer’s protocol. After RLY01 treatment, both attached and floating cells were harvested, washed with PBS, suspended in binding buffer, stained in annexin V–allophycocyanin (APC) and propidium iodide (PI), and analyzed by flow cytometry. For each experiment, 1 × 10^6^ cells/mL were analyzed by the flow cytometer (BD Biosciences) and analyzed with FlowJo v10.8.1 (BD Biosciences).

### Immunoprecipitation.

Cells were washed with PBS and lysed with RIPA lysis buffer (50 mM Tris-HCl [pH 7.5], 150 mM NaCl, 1% NP-40, and 0.1% SDS). Cell lysates were then centrifuged for 20 minutes at 13,400*g* at 4°C. To immunoprecipitate protein, 10% of supernatant was reserved as the input, and the rest was incubated with beads for 6–12 hours at 4°C. The beads were washed 3 times with washing buffer (150 mM NaCl, 50 mM Tris-HCl [pH 7.5], 10% glycerol, 1 mM EDTA). After centrifugation, the beads were boiled in SDS loading buffer, followed by Western blotting.

### Yeast 2-hybrid assay.

The bait vector pGBKT7-REGγ and the prey vector pGADT7-αx were constructed. pGBKT7-REGγ and pGADT7-αx were cotransformed into yeast Y2H Gold–competent cells (Takara Biomedical Technology), plated on DDO/X/A (SD-Trp-Leu/X-α-gal/AbA) medium, and cultured at a constant temperature of 30°C for 3–5 days for preliminary screening. Blue colonies were selected and inoculated on QDO/X (SD-Trp-Leu-His-Ade/X-α-gal) medium for further culture. For more stringent screening of positive clones, pGBKT7-REGγ plus pGADT7-NIP30 was used as the positive control, and pGBKT7 plus pGADT7 was used as the negative control.

### Luciferase reporter assay.

Promoter sequence of REGγ was cloned into pGL3-basic luciferase reporter plasmid. REGγ-luciferase reporter together with either siCtrl or si*NRF2* was transfected into A549 cells. At 48–72 hours after transfection, the luciferase activities in cell lysates were measured with the luciferase assay system (BMG Labtech). Results are expressed as fold change and represent the mean ± SEM of 3 independent experiments.

### ChIP-qPCR.

A total of 10^7^ A549 cells were harvested in PBS after formaldehyde cross-linking. After centrifugation, protease inhibitor–containing SDS lysis buffer was added to the cell pellet. Nuclear lysates were sonicated to shear DNA to around 500 bp, followed by immunoprecipitation for 16 hours at 4°C using anti-NRF2 antibody (Abcam, ab137550). After purification, qPCR was performed to detect the protein binding sites of the DNA samples.

### CUT&Tag assays.

HCT116 live cells (cell viability over 90%) were collected and DNA libraries were prepared using the Vazyme Hyperactive Universal CUT&Tag kit (TD904, Vazyme). The DNA libraries were sequenced through a sequencing service using Illumina Pro to obtain NRF2 transcription factor binding peak data.

### Electrophoretic mobility shift assay.

NRF2 protein (MedChemExpress, HY-P72308), IRDye 700–labeled (Shanghai Rui Mian Biological Technology Co.) double-stranded DNA, and competitor oligonucleotides were incubated for 30 minutes at room temperature with poly(dI-dC) in the binding buffer (20 mM HEPES, 4 mM MgCl_2_, 100 μg/mL bovine serum albumin, 4% glycerol, 20 mM KCl, 5 mM DTT, and 1 mM EDTA). Samples were electrophoresed through a native acrylamide gel in 1× TBE buffer (89 mM Tris [pH 7.6], 89 mM boric acid, 2 mM EDTA). Gels were imaged using the Odyssey infrared imaging system (LI-COR Biosciences).

### Structure-based virtual screening.

Docking was carried out using Schrödinger Glide software. The cryogenic electron microscopic (cryo-EM) structure of the REGγ-20S proteasome (Protein Data Bank code 7NAO) (52) was prepared using the protein preparation wizard module in Schrödinger’s Maestro interface, with a pH of 7.0 ± 1.0. Meanwhile, REGγ subunits were removed from the complex during the preparation process. The fpocket server was used to detect druggable pockets (53). The druggability score was calculated to assess the possibility of a pocket to accommodate drug-like molecules. A score higher than 0.5 (the threshold) means the pocket might be druggable. Based on the druggable pocket, we screened the SPECS database, which contains approximately 220,000 compounds that lacked pan-assay interference compounds (PAINS). The compounds were prepared and tautomerized at pH 7.0 using the LigPrep module of the Schrödinger software suite to generate energy-minimized 3D molecular structures. These 3D structures were then used for docking studies. The prepared compound databases were docked into the predicted druggable pocket of 20S proteasome using high-throughput virtual screening, followed by standard-precision (SP) and extra-precision (XP) docking modules in Glide. The OPLS3 force field was used to parameterize both ligands and protein (54). The 1,000 molecules with the highest docking and Glide scores from XP docking were visually inspected. To ensure diversity in molecular structures, binding modes, and drug-like properties, 100 compounds were selected for bioassay and purchased with a purity of over 95%.

### Pharmacokinetics study.

Pharmacokinetics of RLY01 was analyzed in male BALB/c mice (*n* = 3). Plasma concentration was determined using liquid chromatography–tandem mass spectrometry (LC-MS/MS) after a single intraperitoneal injection dose (20 mg/kg, i.p.) of a compound as a clear solution (2% DMSO plus 2% Tween-80 plus 96% saline) at a volume of 10 mL/kg. Blood samples were collected into a heparinized test tube at each time point (0.083, 0.25, 0.5, 1.0, 2.0, 4.0, 8.0, and 24 hours) and centrifuged at 8,000*g* for 10 minutes to generate plasma samples. The blood samples were temporarily put on ice. Plasma samples were put on dry ice for 2 hours and transferred to –20°C. After the completion of the last sampling, all samples were stored at –80°C. LC-MS/MS methods to quantify compound in plasma samples were developed. The samples were analyzed with an Agilent 1290 Infinity II HPLC system coupled to a 6470 LC/TQ mass spectrometer (Agilent), which was equipped with an Applied Biosystems electrospray ionization source and operated with Analysis of Mass Hunter Workstation Data Acquisition (Agilent). Chromatographic separation was fitted with an Agilent Poroshell 120 EC-C18, 2.1 × 50 mm, 1.9 μm column. The mobile phase consisted of 0.1% formic acid in water and 0.1% formic acid in acetonitrile (ACN). Standard curves for drug quantification were prepared by spiking known concentrations of the compound into control plasma. Area under the curve, T_max_, C_max_, T_1/2_, where T_max_ indicates time to maximum concentration, C_max_ indicates maximum concentration achieved by RLY01 in the blood or plasma after administration, and T_1/2_ indicates elimination half life, and mean retention time were calculated by non-compartmental analysis using WinNonlin (Phoenix version 6.1, Pharsight Corp.) with mean concentration at each time point.

### Surface plasmon resonance assay.

Surface plasmon resonance–based ligand binding assays were performed using a Biacore T200 system (GE Healthcare Life Sciences). 20S proteasome was diluted with acetate (pH 7.5) and conjugated to a Series S Sensor Chip CM5 (GE Healthcare Life Sciences, BR100530) by EDC/NHS cross-linking reaction according to the manufacturer’s protocols. The target immobilization level of protein was 25 RU. The small molecule RLY01 was diluted with a running buffer containing 5% DMSO from 0.125 to 64 μM, and was injected into the reference channel and 20S proteasome channel, respectively, at a flow rate of 30 μL/min. The coupling and dissociation times were both 120 seconds. Biacore T200 evaluation software was used to fit the affinity curves by the steady-state affinity model (1:1), and the equilibrium dissociation constant (*K_D_*) was calculated.

### Drug affinity responsive target stability.

HEK293T cells at a density of 90% in a 10 cm dish were scraped and lysed with M-PER reagent (Thermo Fisher Scientific, 78501). After centrifugation for 10 minutes at 12,000*g*, the supernatant was obtained and was mixed with TNC buffer (50 μM Tris-HCl pH 8.0, 50 μM sodium chloride, 10 μM calcium chloride). Samples of each group were treated with RLY01 and DMSO for 60 minutes. Samples were then incubated with pronase (Roche, PRON-RO) in different proportions for 5 minutes at 37°C. All portions of each sample were used for Western blot analysis.

### Streptavidin-biotin affinity pull-down assay.

Cell lysates were incubated with free biotin (MedChemExpress) or biotin-RLY01 for 3 hours at room temperature with rotation. Subsequently, the prewashed streptavidin agarose beads (Yeasen Biotech) were added to the system as above and incubated 1 hour at room temperature with rotation. The beads were washed 3 times with elution buffer and then were boiled in SDS loading buffer, followed by Western blotting.

### In-gel digestion and MS analysis.

LC-MS/MS–based proteomics analysis was conducted in collaboration with Shanghai Applied Protein Technology Co. Ltd. According to the methods described for streptavidin-biotin affinity pull-down assay, biotin-RLY01 was used to pull down target proteins from the whole-cell lysates. SDS-PAGE gels were stained with Silver Staining kits (Beyotime, P0017S). The entire gel was cut into pieces at the target sites (Supplemental Figure 6F, black arrows), which were destained, reduced, and alkylated, followed by trypsin digestion. The digested peptides were extracted, resuspended in 0.1% formic acid, and analyzed by LC-MS/MS on a Q-Exactive mass spectrometer (Thermo Electron). The raw MS data for each sample were combined and searched using MaxQuant 1.6.14 software for identification and quantitation analysis. The minimal scores for peptide and protein were set as 20 and 50, respectively. The FDR was set to 0.01 for analysis.

Refer to Supplemental Table 2 for raw data of 4D label-free mass spectrum in Supplemental Figure 6F.

### Peptidyl substrate LLVY/LRR degradation assay.

The reaction mixture contained the indicated combinations of 20S proteasome (0.125 μg), 11S (0.0625 μg), and RLY01 (20 μM). This mixture was incubated on ice for 45 minutes in buffer (10 mM Tris-HCl, 10 mM KCl, 4% glycerol, 1 mM DTT). Then, peptidyl substrates (Suc-LLVY-AMC/Boc-LRR-AMC) were incubated for 15–20 minutes in the mixture in a 37°C thermostatic fluorescence spectrophotometer. The amount of AMC during the reaction was measured fluorometrically (excitation of 360 nm and emission of 460 nm for AMC) with a fluorescence spectrophotometer. Proteasome activity was assayed in the presence of the indicated peptides and proteins and normalized by setting to 100% the value obtained in the presence of 20S proteasome and 11S. The respective final concentrations of the 20S proteasome and REGγ or REGα were set up so that the peptidase activities were not fully activated (i.e., 11S was limiting in the assay). At the final concentration used for RLY01, the inhibitory effect was maximal. Peptides used were substrates of the following peptidase activities of the 20S proteasome: chymotrypsin-like activity, Suc-LLVY-AMC (LLVY); trypsin-like activity, Boc-LRR-AMC (LRR).

### LoxP-stop-loxP Kras^G12D^ Trp53^fl/fl^ mouse models.

*LoxP*-stop-*loxPKras^G12D^*/FVB/129 *Trp53^fl/fl^* mice (KP mice) were previously generated. Six- to eight-week-old KP mice were inoculated with 1 × 10^6^ PFU adenoviral Cre (adeno-Cre) by intranasal inhalation to activate oncogenic *p53* and *Kras^G12D^* in the lungs. Animal studies were conducted under specific pathogen–free conditions. Experimental mice were handled in compliance with the ethical and scientific standards of the East China Normal University’s Animal Center, following procedures approved by the East China Normal University’s Institutional Animal Care and Use Committee (IACUC). All surgeries were performed under tribromoethanol anesthesia to minimize suffering.

### Cell-derived xenograft and colorectal cancer patient-derived xenograft.

HCT116 and HT29 cells (8.0 × 10^6^ to 9.0 × 10^6^ cells) with or without silencing of REGγ suspended in 100 μL PBS solution were subcutaneously injected into the flanks of 4- to 5-week-old nude male mice. Tumor sizes were measured every 4 days with Vernier calipers. Tumor volumes were calculated using the following formula: *L* × *W*^2^ × 0.52, where *L* represents the large diameter of the tumor and *W* represents the small diameter.

Patient-derived xenograft tumor and HCT15 cells were subcutaneously injected into thirty 4- to 5-week-old nude male mice using the same method as above. After 2 weeks, 24 mice bearing tumors with an average volume of 70 mm³ were selected and randomly assigned into 4 groups, followed by 0, 25, or 50 mg/kg RLY01 or 0.2 mg/kg BTZ intraperitoneal injection per day. Tumor sizes were measured every 3 days, and tumor volumes were calculated. When tumor volume of mice in the vehicle group reached about 1,500 mm^3^, all mice were sacrificed and isolated tumors weighed.

Mouse whole blood was collected from inferior vena cava. Serum was prepared by centrifugation of whole blood to remove clots after incubation at 4°C overnight. Serum urea, creatinine, alanine aminotransferase, and aspartate aminotransferase levels were measured with a VetScan v2 Chemistry Analyzer (Abaxis).

### Immunohistochemistry staining and H&E staining.

Tumor tissues were fixed in 4% paraformaldehyde and paraffin-embedded. Tissue sections were subjected to dewaxing and antigen retrieval, followed by overnight incubation at 4°C with primary antibodies: rabbit anti-REGγ (1:100), rabbit anti-LATS1 (1:50), or mouse anti-p21 (1:50). After staining using secondary antibody at room temperature for 20 minutes, visualization was performed using DAB and microscope (Olympus BX53; Olympus). Blocking solution, secondary antibody, and DAB were from the Histostain-Plus IHC Kit (NeoBioscience, ENS003 and ENS004). Immunohistochemistry (IHC) score was calculated using staining intensity (0 to 3) × percentage of positive cells (0 to 4), yielding a staining index ranging from 0 (no staining) to 12 (extensive, strong staining).

Sections were deparaffinized, rehydrated, and stained with H&E. Stained slides were then evaluated using IX81 microscopy (Olympus).

### Patient-derived organoid viability assay.

*KRAS^G12F^* lung cancer patient-derived organoids were previously established and characterized by OneTar Biomedicine. Tumor organoids were digested into single cells in trypsin and were counted. Cells were seeded into 96-well plates at a density of 2,000 cells per well and were treated with RLY01 or vehicle for 96 hours after 3 days. Organoid was stained in the dark with propidium iodide (PI) and calcein AM at 37°C for 30 minutes and then assayed using a fluorescence microscope. Green fluorescence represents calcein AM staining for live cells, while red fluorescence represents PI staining for dead cells. The ethical approval number is bc20240034, where ‘2024’ indicates the year of approval, and ‘0034’ is the patient sample number assigned in that year.

### Statistics.

Numbers of biological samples included in analyses are listed throughout figure legends. Statistical analyses were performed using GraphPad Prism, version 8.0.2. Unpaired, 2-tailed Student’s *t* test was performed between 2 parametric groups. One-way ANOVA was used to compare multiple groups with a designated control. For multiple groups of more than 1 variable, 2-way ANOVA was used. A *P* value of less than 0.05 was considered significant. *P* values are denoted as follows: *P < 0.05, **P < 0.01, ***P < 0.001, ****P < 0.0001.

### Study approval.

All animal treatments were performed according to the *Guide for the Care and Use of Laboratory Animals* (National Academies Press, 2011). All animal procedures were approved by East China Normal University and were performed in accordance with East China Normal University’s IACUC guidelines.

### Data availability.

Original Western blots are available in the associated raw data file “WB Unedited blot and gel images.” Sources of all antibodies and cell lines used, full differential expression analysis, and pathway analysis can be found in the supplemental material. The reagents used are listed in Supplemental Table 9. The information of patient samples is listed in Supplemental Table 10. MS identification of differential proteins in HCT8-*KRAS^G13D^* cells compared with HCT8-*KRAS^WT^* cells, MS identification of pathway enrichment in HCT8-*KRAS^G13D^* cells, and MS identification of RLY01 binding proteins are presented in Supplemental Tables 2, 3, 7, and 8. Values for all data points in graphs are reported in the Supporting Data Values file. The MS proteomics data were deposited to the ProteomeXchange database (https://proteomecentral.proteomexchange.org) via the iProX partner repository (55, 56) with the dataset identifier PXD059364. The ChIP-Seq data reported here were deposited in the NCBI’s Gene Expression Omnibus database (GEO GSE284241).

## Author contributions

SS, QZ, KL, and L Li were responsible for methodology, writing: original draft, and writing: review and editing. QZ, KL, L Li, and HY were responsible for conceptualization and supervision. SS and QZ, ML, SZ, and JS were responsible for software. SS, QZ, and KL were responsible for investigation. SS, QZ, YL, ML, and KL were responsible for formal analysis. SS, YW, EZ, CG, SG, BY, L Liu, SC, MZ, WR, ZP, LZ, Hansen Chen, and Hui Chen were responsible for validation. SS, QZ, YW, and KL were responsible for resources. PKHL was responsible for writing: review and editing. BZ, QZ, L Li, and HY were responsible for funding acquisition.

## Supplementary Material

Supplemental data

Unedited blot and gel images

Supplemental tables 1-10

Supporting data values

## Version 1 03/17/2025

Electronic publication

## References

[1] Cook JH (2021). The origins and genetic interactions of KRAS mutations are allele- and tissue-specific. Nat Commun.

[2] Jiang J (2024). Translational and therapeutic evaluation of RAS-GTP inhibition by RMC-6236 in RAS-driven cancers. Cancer Discov.

[3] Weng C (2024). The energetic and allosteric landscape for KRAS inhibition. Nature.

[4] Simanshu DK (2017). RAS proteins and their regulators in human disease. Cell.

[5] Huang L (2021). KRAS mutation: from undruggable to druggable in cancer. Signal Transduct Target Ther.

[6] Hallin J (2020). The KRAS^G12C^ inhibitor MRTX849 provides insight toward therapeutic susceptibility of KRAS-mutant cancers in mouse models and patients. Cancer Discov.

[7] Blair HA (2021). Sotorasib: first approval. Drugs.

[8] Yaeger R (2023). Adagrasib with or without cetuximab in colorectal cancer with mutated *KRAS* G12C. N Engl J Med.

[9] Strickler JH (2023). Sotorasib in *KRAS* p.G12C-mutated advanced pancreatic cancer. N Engl J Med.

[10] Wang X (2022). Identification of MRTX1133, a noncovalent, potent, and selective KRAS^G12D^ inhibitor. J Med Chem.

[11] Kim D (2023). Pan-KRAS inhibitor disables oncogenic signalling and tumour growth. Nature.

[12] Kemp SB (2023). Efficacy of a small-molecule inhibitor of KrasG12D in immunocompetent models of pancreatic cancer. Cancer Discov.

[13] Zhou C (2024). Anti-tumor efficacy of HRS-4642 and its potential combination with proteasome inhibition in KRAS G12D-mutant cancer. Cancer Cell.

[14] Yaeger R (2022). Targeting alterations in the RAF-MEK pathway. Cancer Discov.

[15] Ryan MB (2020). Vertical pathway inhibition overcomes adaptive feedback resistance to KRAS^G12C^ inhibition. Clin Cancer Res.

[16] Anderson DJ (2015). Targeting the AAA ATPase p97 as an approach to treat cancer through disruption of protein homeostasis. Cancer Cell.

[17] Van Drie JH (2011). Protein folding, protein homeostasis, and cancer. Chin J Cancer.

[18] Mohanty A (2023). Acquired resistance to KRAS G12C small-molecule inhibitors via genetic/nongenetic mechanisms in lung cancer. Sci Adv.

[19] Richardson PG (2006). Bortezomib: proteasome inhibition as an effective anticancer therapy. Annu Rev Med.

[20] Harshbarger W (2015). Crystal structure of the human 20S proteasome in complex with carfilzomib. Structure.

[21] Fricker LD (2020). Proteasome inhibitor drugs. Annu Rev Pharmacol Toxicol.

[22] Thomas TA, Smith DM (2022). Proteasome activator 28γ (PA28γ) allosterically activates trypsin-like proteolysis by binding to the α-ring of the 20S proteasome. J Biol Chem.

[23] Shen M (2020). Role of oncogenic REGγ in cancer. Biomed Pharmacother.

[24] Lei K (2020). PA28γ, an accomplice to malignant cancer. Front Oncol.

[25] Chen D (2013). The expression and clinical significance of PA28 γ in colorectal cancer. J Investig Med.

[26] He J (2012). REGγ is associated with multiple oncogenic pathways in human cancers. BMC Cancer.

[27] Li L (2015). REGγ is critical for skin carcinogenesis by modulating the Wnt/β-catenin pathway. Nat Commun.

[28] Wang X (2011). REG gamma: a potential marker in breast cancer and effect on cell cycle and proliferation of breast cancer cell. Med Oncol.

[29] Fan Chai YL (2014). High expression of REGγ is associated with metastasis and poor prognosis of patients with breast cancer. Int J Clin Exp Pathol.

[30] Okamura T (2003). Abnormally high expression of proteasome activator-gamma in thyroid neoplasm. J Clin Endocrinol Metab.

[31] Cascio P (2021). PA28γ: new insights on an ancient proteasome activator. Biomolecules.

[32] Tong L (2019). Proteasome-dependent degradation of Smad7 is critical for lung cancer metastasis. Cell Death Differ.

[33] DeNicola GM (2011). Oncogene-induced Nrf2 transcription promotes ROS detoxification and tumorigenesis. Nature.

[34] Ohta T (2008). Loss of Keap1 function activates Nrf2 and provides advantages for lung cancer cell growth. Cancer Res.

[35] Li X (2007). Ubiquitin- and ATP-independent proteolytic turnover of p21 by the REGgamma-proteasome pathway. Mol Cell.

[36] Kim EM (2017). The p53/p21 complex regulates cancer cell invasion and apoptosis by targeting Bcl-2 family proteins. Cancer Res.

[37] Skoulidis F (2021). Sotorasib for lung cancers with *KRAS* p.G12C mutation. N Engl J Med.

[38] Tanaka N (2021). Clinical acquired resistance to KRAS^G12C^ inhibition through a novel KRAS switch-II pocket mutation and polyclonal alterations converging on RAS-MAPK reactivation. Cancer Discov.

[39] Reck M (2021). Targeting KRAS in non-small-cell lung cancer: recent progress and new approaches. Ann Oncol.

[40] Ali A (2013). Differential regulation of the REGγ-proteasome pathway by p53/TGF-β signalling and mutant p53 in cancer cells. Nat Commun.

[41] Gao X (2020). The REGγ inhibitor NIP30 increases sensitivity to chemotherapy in p53-deficient tumor cells. Nat Commun.

[42] Wang H (2015). Mutant p53 (p53-R248Q) functions as an oncogene in promoting endometrial cancer by up-regulating REGγ. Cancer Lett.

[43] Zhang Y (2015). Oxidative challenge enhances REGγ-proteasome-dependent protein degradation. Free Radic Biol Med.

[44] Ma Q (2013). Role of nrf2 in oxidative stress and toxicity. Annu Rev Pharmacol Toxicol.

[45] Rojo de la Vega M (2018). NRF2 and the hallmarks of cancer. Cancer Cell.

[46] Gwinn DM (2018). Oncogenic KRAS regulates amino acid homeostasis and asparagine biosynthesis via ATF4 and alters sensitivity to L-asparaginase. Cancer Cell.

[47] Shirazi F (2020). Activating *KRAS*, *NRAS*, and *BRAF* mutants enhance proteasome capacity and reduce endoplasmic reticulum stress in multiple myeloma. Proc Natl Acad Sci U S A.

[48] Yang H (2021). Repurposing old drugs as new inhibitors of the ubiquitin-proteasome pathway for cancer treatment. Semin Cancer Biol.

[49] Richardson PG (2005). Bortezomib or high-dose dexamethasone for relapsed multiple myeloma. N Engl J Med.

[50] Chanan-Khan A (2008). Analysis of herpes zoster events among bortezomib-treated patients in the phase III APEX study. J Clin Oncol.

[51] Chou TC (2010). Drug combination studies and their synergy quantification using the Chou-Talalay method. Cancer Res.

[52] Zhao J (2022). Structural insights into the human PA28-20S proteasome enabled by efficient tagging and purification of endogenous proteins. Proc Natl Acad Sci U S A.

[53] Schmidtke P (2010). fpocket: online tools for protein ensemble pocket detection and tracking. Nucleic Acids Res.

[54] Harder E (2016). OPLS3: a force field providing broad coverage of drug-like small molecules and proteins. J Chem Theory Comput.

[55] Ma J (2019). iProX: an integrated proteome resource. Nucleic Acids Res.

[56] Chen T (2022). iProX in 2021: connecting proteomics data sharing with big data. Nucleic Acids Res.

