# Supplementary material for: Targeting ubiquitin-independent proteasome with small molecule increases susceptibility in pan-KRAS–mutant cancer models
Source: J Clin Invest. 2025 Mar 17;135(6):e185278. doi: 10.1172/JCI185278 (PMC11910216; doi:10.1172/JCI185278)
Supplement: Supplemental data [file jci-135-185278-s150.pdf]

Supplementary Materials for

Targeting ubiquitin-independent proteasome with small molecule increases susceptibility in pan-KRAS mutant cancer models

Shihui Shen[1,2,6], Qiansen Zhang[1,6,*], Yuhan Wang[1,6], Hui Chen[2,6], Shuangming Gong[1], Yun Liu[1], Conghao Gai[3], Hansen Chen[1], Enhao Zhu[1], Bo Yang[1], Lin Liu[1], Siyuan Cao[1], Mengting Zhao[1], Wenjie Ren[1], Mengjuan Li[1], Zhuoya Peng[1], Lu Zhang[1], Shaoying Zhang[1], Juwen Shen[1], Bianhong Zhang[1], ZPatrick K. H. Lee[4], Kun Li [5,*], Lei Li[2,*] and Huaiyu Yang[1,*]

[1] Shanghai Key Laboratory of Regulatory Biology, Institute of Biomedical Sciences, School of Life Sciences, East China Normal University, 500 Dongchuan Road, Shanghai 200241, China.

[2] Joint Center for Translational Medicine, Shanghai Fifth People's Hospital, Fudan University and School of Life Science, East China Normal University, Shanghai 200240, China.

[3] Organic Chemistry Group, College of Pharmacy, Naval Medical University, Shanghai, 200433, China.

[4] School of Energy and Environment, City University of Hong Kong, Hong Kong SAR, China.

[5] Health Science Center, East China Normal University, Shanghai 200241, China.

[6] These authors contributed equally to this work.

[*]Corresponding author. Email: qszhang@bio.ecnu.edu.cn (Q.Z.), kli@hsc.ecnu.edu.cn (K.L.), lli@bio.ecnu.edu.cn (L.L.), hyyang@bio.ecnu.edu.cn (H.Y.)

The authors have declared that no conflict of interest exists.

This supplementary material contains

1. Supplemental Figure 1. Elevated REGγ protein expression in different KRAS-mutant cancer cells and *REGγ* gene expression correlated with *KRAS* mRNA expression.

2. Supplemental Figure 2. Overexpression (OE) and knockdown efficiency of *REGγ* by immunoblot analysis in different cancer cells.

3. Supplemental Figure 3. NRF2 binds to the *REGγ* promoter and upregulates REGγ expression in KRAS-mutant cells.

4. Supplemental Figure 4. Blockade of key components downstream of KRAS decreases REGγ and NRF2 expression.

5. Supplemental Figure 5. The interaction of REGγ and α7 subunit of 20S core particle.

6. Supplemental Figure 6. Identification of REGγ-proteasome compounds.

7. Supplemental Figure 7. RLY01 blocks REGγ-20S proteasome degradation functions in a REGγ-dependent manner.

8. Supplemental Figure 8. RLY01 suppresses the clonogenic growth and induces cell apoptosis.

9. Supplemental Figure 9. The in vivo therapeutic effect of RLY01 in KRAS-mutant tumor suppression.

10. Supplemental Figure 10. The combined cytotoxicity of RLY01 and AMG510.

11. Graphic abstract.

12. Other Supplementary Material for this manuscript includes the following:

Supplemental Table 1-9

Supplemental Figure 1

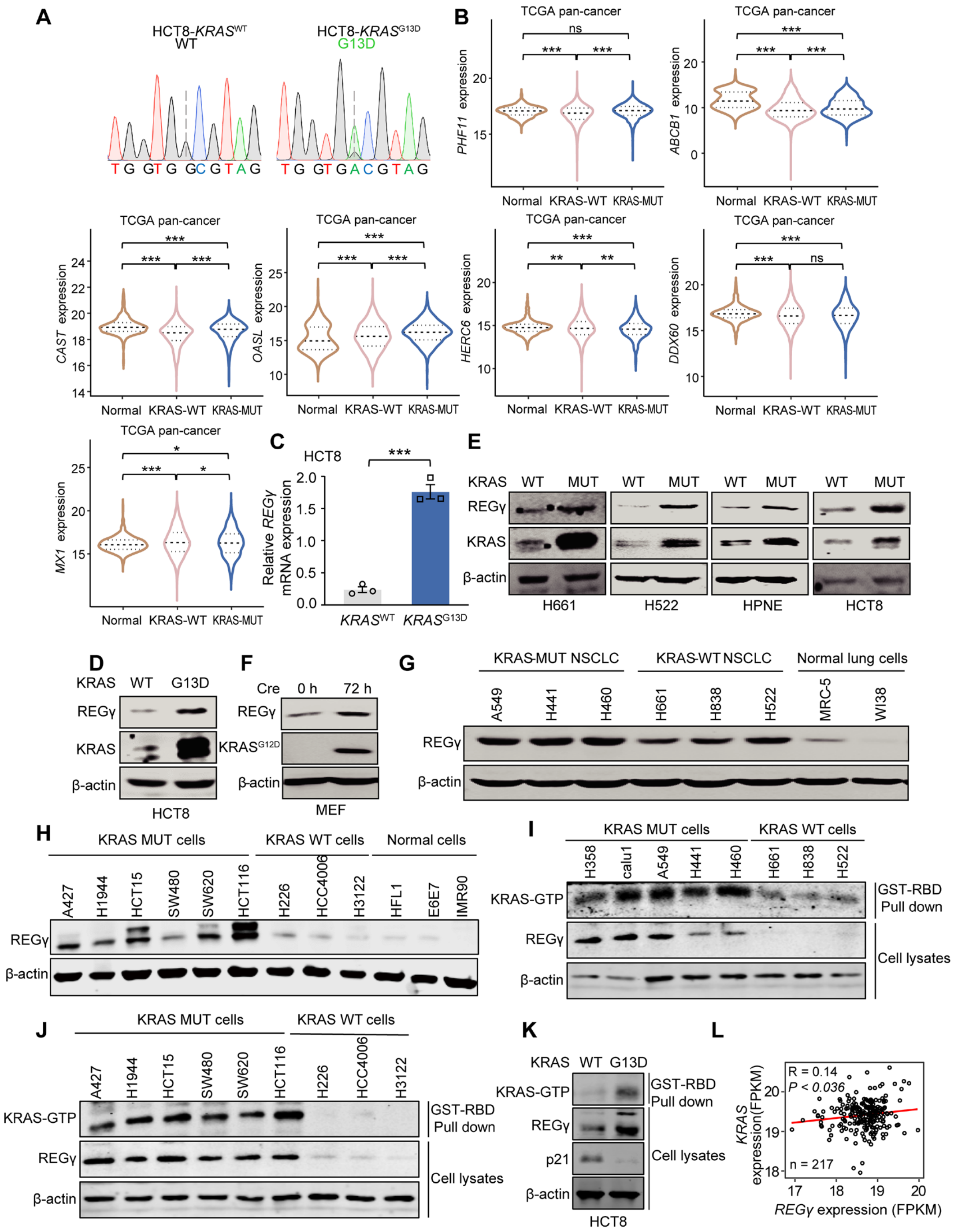

Supplemental Figure 1. Elevated REGγ protein expression in different KRAS-mutant cancer cells and *REGγ* gene expression correlated with *KRAS* mRNA expression, related to Figure 1. (**A**) DNA-Seq of exon 2 in the *KRAS* gene. (**B**) Violin plots depicting distribution of the top 10 identified proteins expression level in Normal tissues (741), KRAS-WT cancers (8661) tissues and KRAS-MUT (769) cancers tissues. Datasets of pan-cancer were derived from the TCGA. (**C** and **D**) KRAS mutant upregulated REGγ expression at the mRNA (**C**) and protein (**D**) levels. HCT8 cells were transfected with *KRAS*^{G13D} mutant variants. Each value represents mean ± SEM (n = 3).

***$p < 0.001$, p values were measured by unpaired, 2-tailed Student's t tests. Representative blots are shown from 3 independent experiments. (**E** and **F**) REGγ protein level is upregulated with KRAS mutation (*KRAS*^{G13D}) occur in H661, H522, HPNE, HCT8 cells (**E**) and MEF cells obtained from Ad-Cre activated *KRAS*^{G12D}, p53$^{flox/flox}$ (KP) mice (**F**). β-actin is a control for protein loading. (**G** and **H**) REGγ protein level is positive correlated with KRAS mutation in different cancer cells. β-actin is a control for protein loading. (**I-K**) REGγ and KRAS-GTP protein expression in various cell lines by immunoblot analysis. KRAS-GTP expression levels were determined using GST-RBD, the GST-fusion of the RAS binding domain of c-RAF, to pull down active GTP-bound KRAS from cellular lysates by glutathione beads. (**L**) The gene expression information from 217 cases of KRAS-mutant colorectal carcinoma patients in The Cancer Genome Atlas (TCGA), reflecting the positive correlation between REGγ and KRAS expression.

Supplemental Figure 2

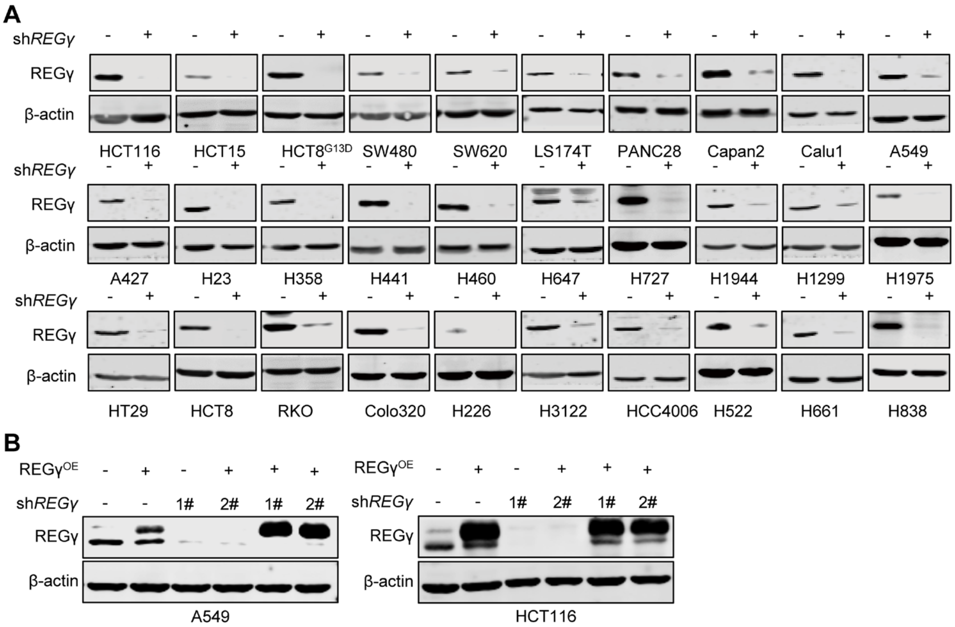

Supplemental Figure 2. Overexpression (OE) and knockdown efficiency of *REGγ* by immunoblot analysis in different cancer cells. (**A**) The silencing efficiency of *REGγ*. (**B**) Overexpression (OE) and knockdown efficiency of *REGγ* by immunoblot analysis in A549 and HCT116 cells.

Supplemental Figure 3

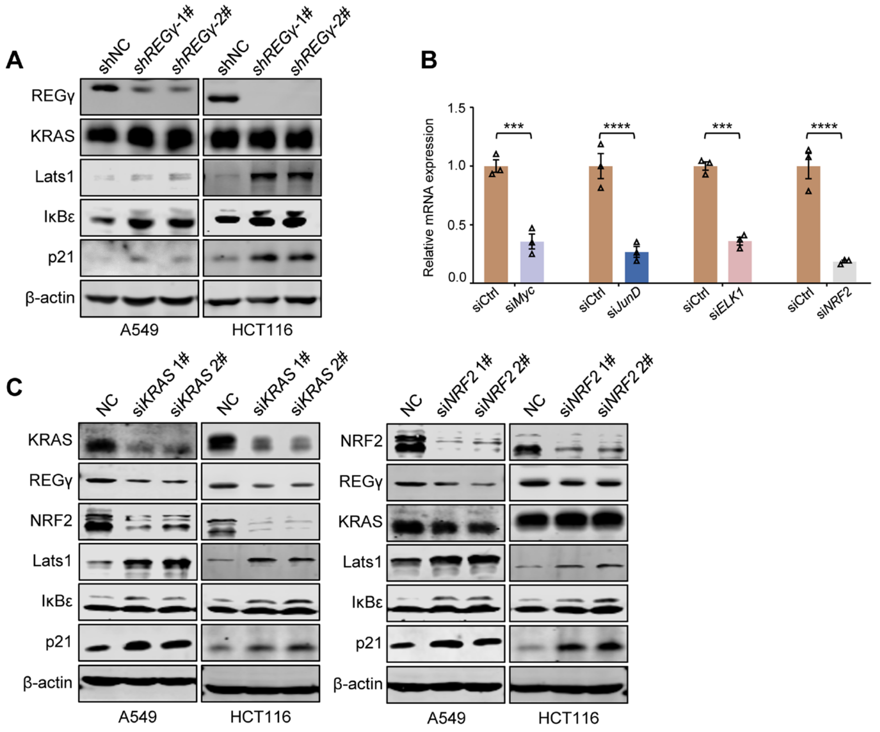

Supplemental Figure 3. NRF2 binds to the *REGγ* promoter and upregulates REGγ expression in KRAS-mutant cells, related to Figure 3. (**A**) The expression of KRAS and REGγ-proteasome substrates (Lats1、IκBε、p21) in A549 (*KRAS*^{G12S}) and HCT116 (*KRAS*^{G13D}) cells upon REGγ silencing using western blot. (**B**) Real-time PCR showing the silencing efficiency of transcription factors. Results are shown as mean ± SEM (n = 3). *p < 0.05, **p < 0.01, ***p < 0.001, ****p < 0.0001; p values were measured by one-way ANOVA with Tukey's multiple-comparisons test. (**C**) The expression of KRAS, NRF2 and REGγ-proteasome substrates (Lats1、IκBε、p21) in A549 (*KRAS*^{G12S}) and HCT116 (*KRAS*^{G13D}) cells upon KRAS and NRF2 silencing using western blot.

Supplemental Figure 4

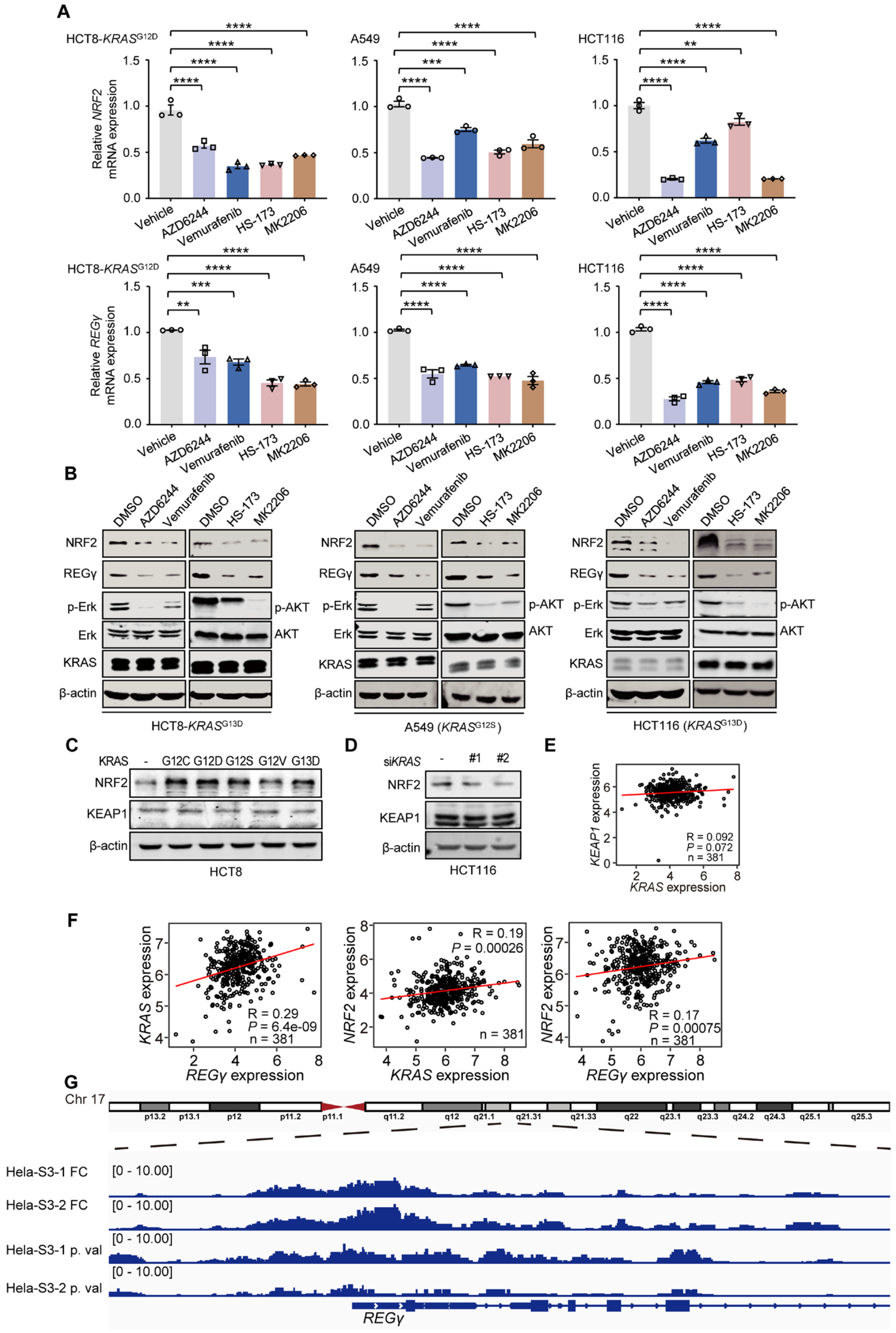

Supplemental Figure 4. Blockade of key components downstream of KRAS decreases REGγ and NRF2 expression, related to Figure 3. (**A** and **B**) REGγ and NRF2 expression upon blockade of KRAS signaling at the mRNA (**A**) and protein levels (**B**). AZD6244, MEK inhibitor; vemurafenib, B-RAF inhibitor; HS-173, PI3K inhibitor; MK2206, AKT inhibitor. HCT8-*KRAS*^{G13D}, A549 (*KRAS*^{G12S}), HCT116 (*KRAS*^{G13D}) cells were treated with AZD6244 (0.5 μM, MEK inhibitor), Vemurafenib (1 μM, Raf inhibitor), HS-173 (1 μM, PI3K inhibitor), and MK2206 (1 μM, AKT

inhibitor) for 24 hours, respectively. Results are shown as mean ± SEM (n = 3). *p < 0.05, **p < 0.01, ***p < 0.001, ****p < 0.0001; p values were measured by one-way ANOVA with Tukey's multiple-comparisons test. (**C** and **D**) Western blot was shown the expression of NRF2 and KEAP1 after overexpression KRAS mutants in HCT8 cell lines or silencing KRAS in HCT116 cell lines. (**E**) The gene expression information from 381 cases of colon, lung, pancreatic and kidney cancers in Depmap datasets, reflecting no substantial difference in correlation between KRAS and KEAP1 expression. (**F**) The gene expression information from 381 cases of colon, lung, pancreatic and kidney cancers in Depmap datasets, reflecting the positive correlation betwee *KRAS* and *REGγ*, *NRF2* and *KRAS* as well as *NRF2* and *REGγ* expression. (**G**) NRF2 ChIP-seq data on Hela-S3 cells from the GEO DataSets database (GSE91997). Genome Browser tracks showed NRF2 ChIP-Seq signals in the *REGγ* (*PSME3*) gene locus.

Supplemental Figure 5

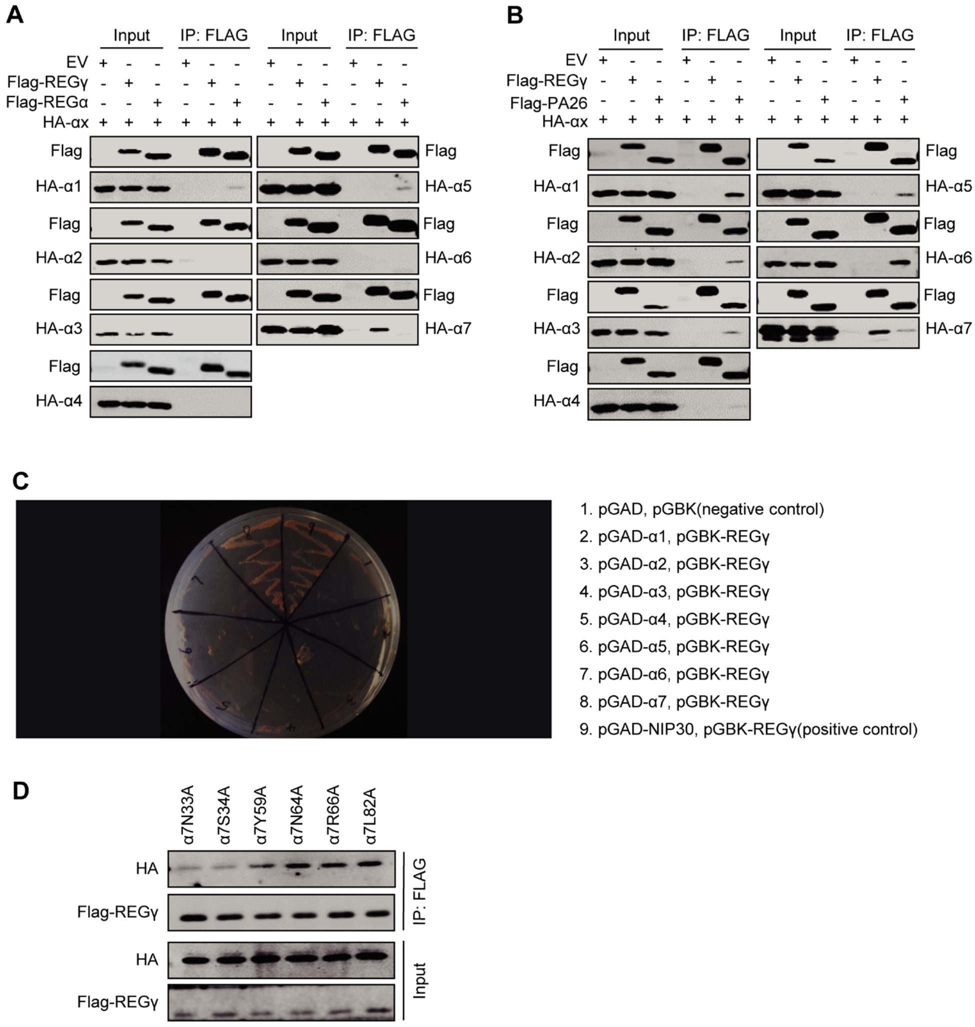

Supplemental Figure 5. The interaction of REGγ and α7 subunit of 20S core particle, related to Figure 4. (**A** and **B**) co-IP showing only REGγ can bind with α7 subunit rather than REGα or PA26. Reciprocal coimmunoprecipitation and western blot analysis following transient overexpression of HA-α1-7, Flag-REGγ or Flag-REGα or Flag-PA26 or control empty vectors. (**C**) Yeast two-hybrid assays. 293T cells co-transformed with pGAD-α1-7 and pGBK-REGγ, or pGAD-NIP30 and pGBK-REGγ, were grown for 3 days at 37°C. Colonies were found for cells co-expressing pGAD-α7 and pGBK-REGγ, or pGAD-NIP30 and pGBK-REGγ. (**D**) Co-IP for the interaction of 6 mutant variants of α7 with REGγ. Transient co-expression of Flag-REGγ and HA-α7N33A or HA-α7S34A or HA-α7Y59A or HA-α7N64A or HA-α7R66A or HA-α7L82A in HCT116 cells for 48 h, cells protein lysates incubated at 4°C for 12 h in vitro and precipitated by FLAG-beads, following detect by western blot analysis.

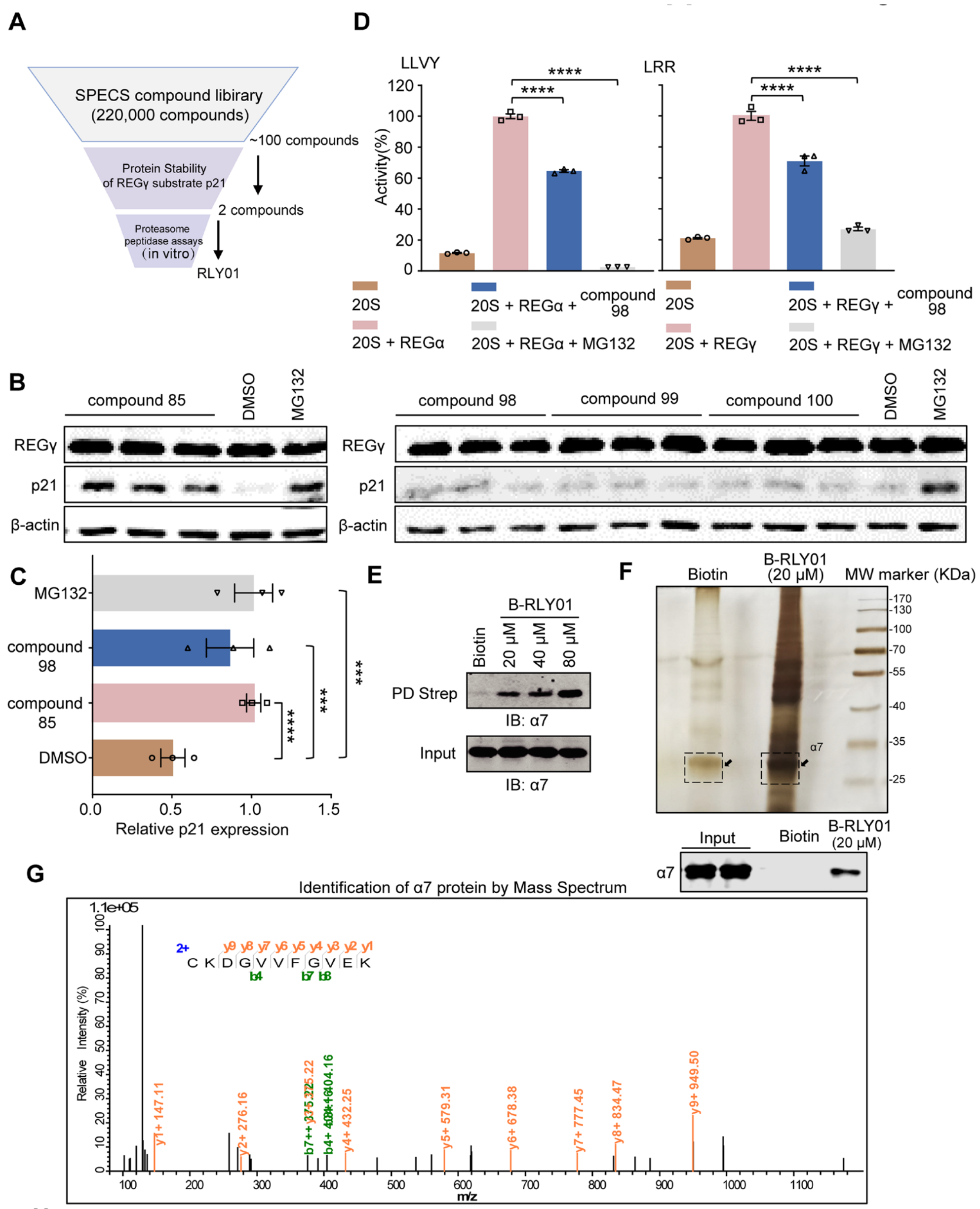

H

IP administration, 20 mg/kg						
Animal	t1/2(hr)	tmax(hr)	Cmax (ng/mL)	AUClast (hr*ng/mL)	AUCInf (hr*ng/mL)	MRTInf (hr)
mouse #1	2.814	0.500	4160	3667	3708	4.804
mouse #2	5.822	0.830	8960	6122	6139	6.151
mouse #3	3.796	0.760	6200	5642	5647	5.580
N	3	3	3	3	3	3
**Mean**	**4.144**	**0.697**	**6440**	**5144**	**5165**	**5.512**
SD	1.534	0.174	2409	1301	1285	0.676
CV%	37.0	25	37.4	25.3	24.9	12.3

Supplemental Figure 6. Identification of REGγ-proteasome compounds, related to Figure 4. (**A**) Workflow of the small molecule screening strategy. (**B**) Western blot images showing compounds 85, 98, 99 and 100 elevate the protein stability of p21 after treated for 24 h. (**C**) Compounds 85 and 98 elevate the protein stability of p21. Each value represents mean ± SEM (n = 3). *p < 0.05, **p < 0.01, ***p < 0.001, ****p < 0.0001; p values were measured by one-way ANOVA with Tukey's multiple-

comparisons test. (**D**) Inhibition by compound 98 of 11S-activated 20S proteasome. Each value represents mean ± SEM (n = 3). *p < 0.05, **p < 0.01, ***p < 0.001, ****p < 0.0001; p values were measured by one-way ANOVA with Tukey's multiple-comparisons test. (**E**) Western blot images showing RLY01 can bind with α7 subunit of 20S proteasome in a dose-dependent way. Immunoblot with anti-α7 antibody of HCT116 cells protein lysates incubated with Biotin or B-RLY01 (20 μM, 40 μM，80 μM) and precipitated by streptavidin beads. (**F**) Identification of proteins associated with RLY01. Up: silver staining of Biotin-RLY01 pull-down proteins. Whole cell lysates were incubated with in vitro biotin and biotin-RLY01. Biotin-RLY01 enriched bands were analyzed by MS (bands in dash lines), n = 3 independent experiment. Biotin was used as negative control. Bottom: Validation of Biotin-RLY01 and α7 interaction by immunoblotting. (**G**) Identification of α7 as the RLY01-binding protein by mass spectrum. Raw data are shown in Supplemental Table 2. (**H**) The pharmacokinetic (PK) property of RLY01 in mice.

Supplemental Figure 7

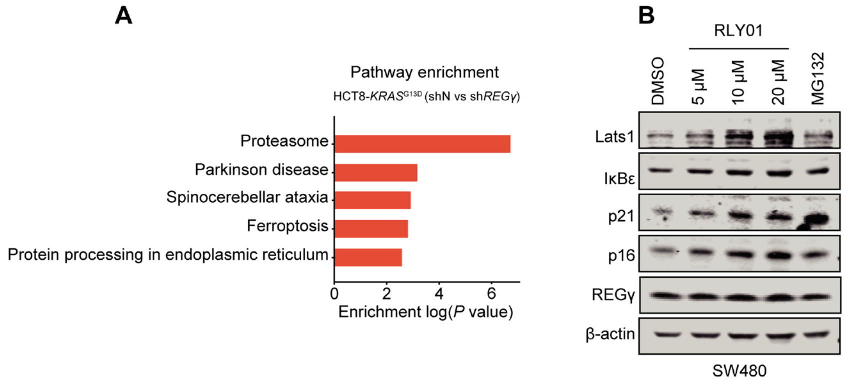

Supplemental Figure 7. RLY01 blocks REGγ-20S proteasome degradation functions in a REGγ-dependent manner, related to Figure 5. (**A**) Pathway enrichment in HCT116 cells after *REGγ*-knockdown in proteomic profiling. (**B**) RLY01 treatment for 12 h promotes REGγ-proteasome substrates Lats1, IκBε, p21, p16 accumulation in SW480 cells. β-actin is a control for protein loading. Representative blots are shown from 3 independent experiments.

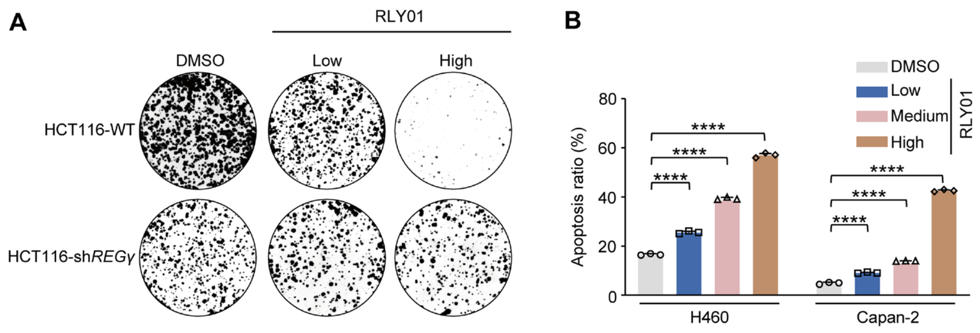

Supplemental Figure 8. RLY01 suppresses the clonogenic growth and induces cell apoptosis, related to Figure 6. (**A**) Representative colony formation images after treatment with RLY01 at their respective 1/4 IC_{50} (Low), IC_{50} (High) in HCT116-WT, HCT116-sh*REGγ* cells. (**B**) H460 and Capan-2 (KRAS-MUT) cells were treated with RLY01 for 48 h, and the percentage of apoptotic cells (Annexin positive) was determined by Annexin-V and propidium iodide staining. Each value represents mean ± SEM ($n = 3$). ****$p < 0.0001$; p values were measured by two-way ANOVA with Tukey's multiple-comparisons test.

Supplemental Figure 9

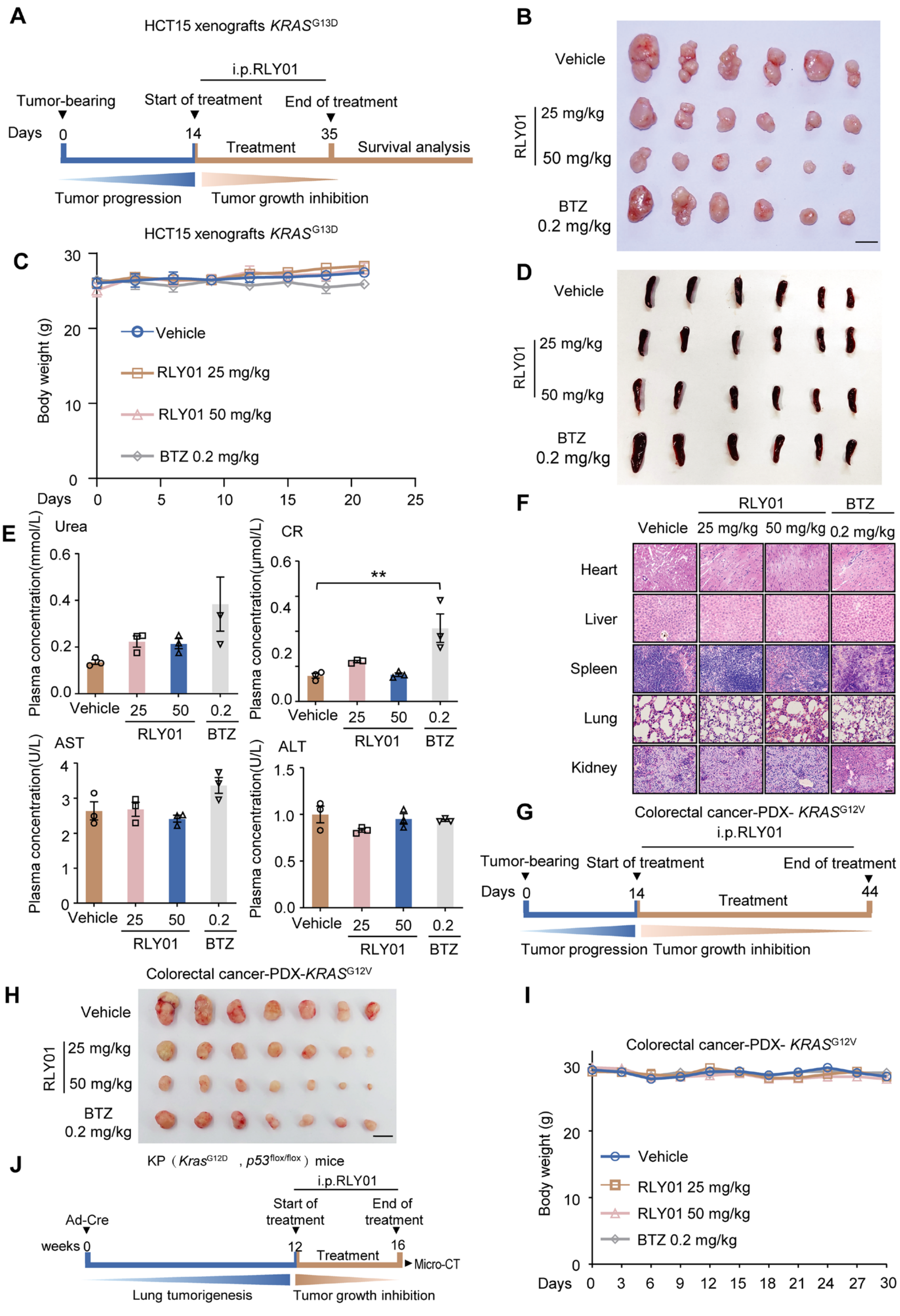

Supplemental Figure 9. The in vivo therapeutic effect of RLY01 in KRAS-mutant tumor suppression, related to Figure 7. (**A**) Schematic illustration of the colorectal carcinoma HCT15 (*KRAS*^{G13D}) CDX xenograft mice and drug treatment protocol. (**B**) Representative photos of colon cancer cell-derived xenograft (CDX) mouse model with or without RLY01 peritoneal injection therapy. (n=6) (**C**) The mouse body weight in the HCT15 xenograft mouse model (n = 6). Data are shown as mean ± SEM. (**D**) Representative photos of Spleen from colon cancer cell-derived xenograft (CDX)

mouse model with or without RLY01 or BTZ peritoneal injection therapy. (n=6) (**E**) Safety measurement by inspection of serum creatinine, suggesting much less toxicity than BTZ. Each value represents mean ± SEM (n = 3). *p < 0.05, **p < 0.01, ***p < 0.001; p values were measured by one-way ANOVA with Tukey's multiple-comparisons test. **(F**) The histological image of tissues from HCT15 xenograft mouse model with or without RLY01 or BTZ peritoneal injection therapy is shown. Scale bar, 50 μm (magnification, ×400). (**G**) Schematic illustration of established colon tumors *KRAS*^{G12V} PDX xenograft mice were treated with vehicle, RLY01. (**H**) Representative photos of patient-derived xenograft (PDX) mouse model with human colorectal carcinoma tissue with or without RLY01 peritoneal injection therapy. (**I**) The mouse body weight in human primary colorectal xenograft mouse model (n = 7). Data are shown as mean ± SEM. (**J**) Schematic illustration of the LSL-*Kras*^{G12D}; *Trp53*$^{flox/flox}$ (KP) allele and drug treatment protocol. KP mice were induced with adenovirus-Cre. After a 12-week induction, mice were treated with indicated compounds for an additional 4 weeks.

Supplemental Figure 10

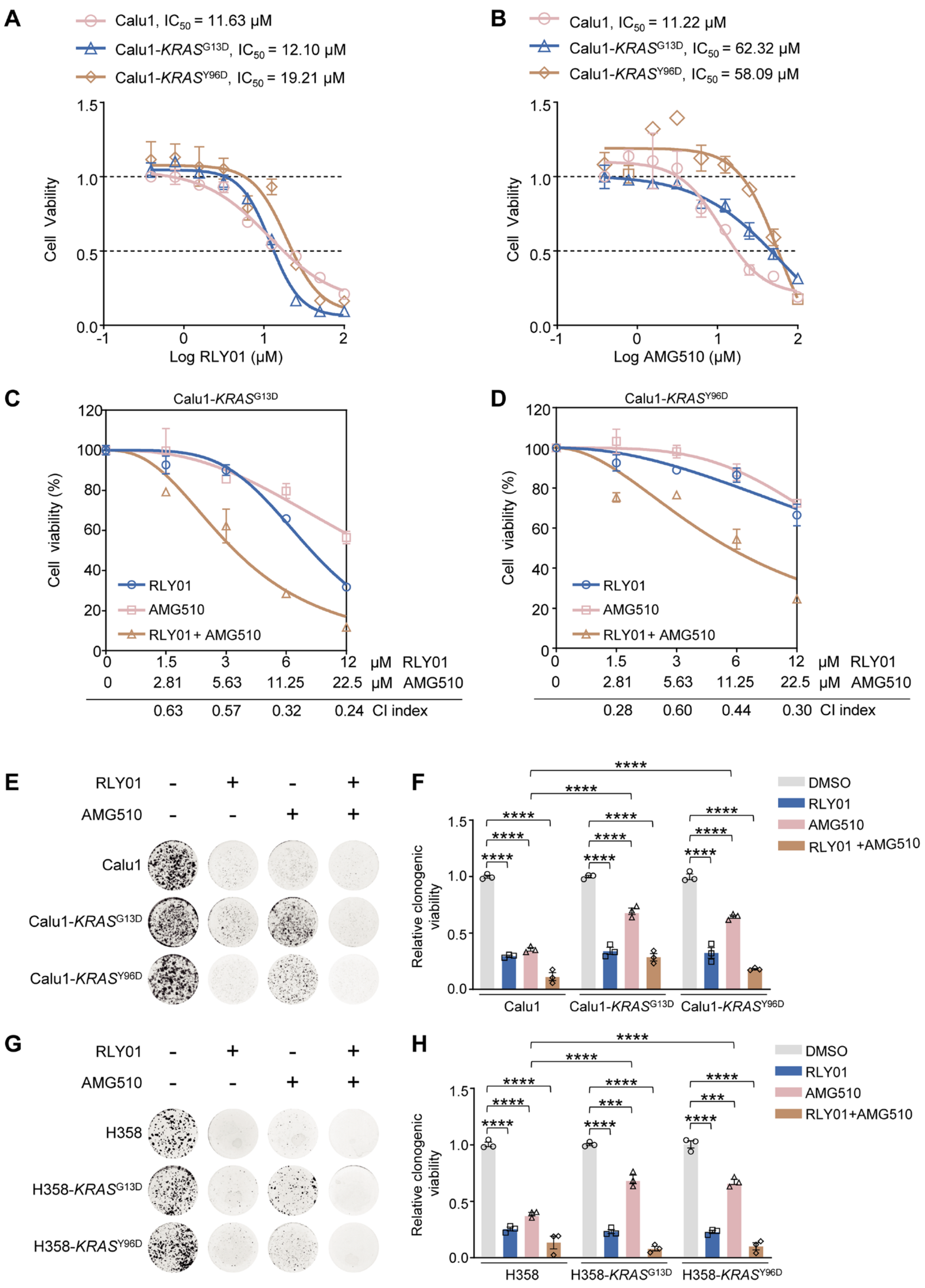

Supplemental Figure 10. The combined cytotoxicity of RLY01 and AMG510, related to Figure 7. (**A** and **B**) Cell viability assays performed with Calu1 cells infected with retrovirus packaging KRAS (G12C, G12C/G13D or G12C/Y96D). Cell lines were treated with indicated RLY01 (**A**) or AMG510 (**B**) for 72 hours and the viabilities were measured with CellTiter-Glo. (**C** and **D**) Calu1-*KRAS*^{G13D} (**C**) and Calu1-*KRAS*^{Y96D} cells (**D**) were incubated with increasing concentrations of RLY01 and AMG510 (*KRAS*^{G12C} inhibitor) alone or in combination for 72 hours, and the cell viability was determined. The CI values for the combination of RLY01 and AMG510 were calculated

by using CompuSyn software (Version 1). The averages and error bars represent the mean ± SEM from three independent experiments. (**E-H**) Representative colony formation images of Calu1, Calu1-*KRAS*^{G13D} and Calu1-*KRAS*^{Y96D} cells (**E**) and H358, H358-*KRAS*^{G13D} and H358-*KRAS*^{Y96D} cells (**G**) cell with or without RLY01 and AMG510 treatment. The relative viability of cultured colonies in these cells (mentioned in **E** and **G**) are shown in **F** and **H**. The percent cell viability is relative to the untreated controls. Each value represents mean ± SEM (n = 3). *$p < 0.05$, **$p < 0.01$, ***$p < 0.001$, ****$p < 0.0001$; p values were measured by two-way ANOVA with Tukey's multiple-comparisons test.

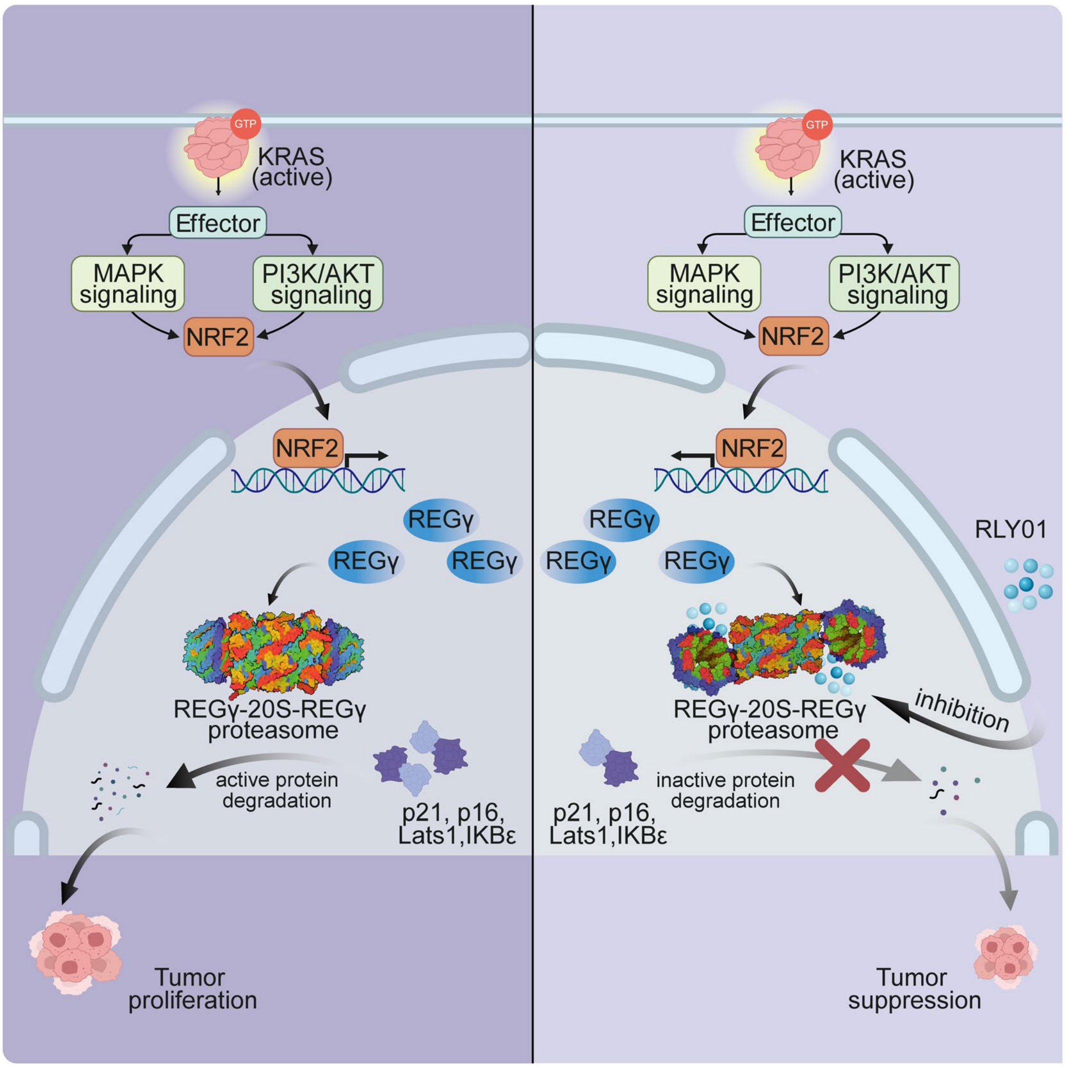

Supplemental Figure 11. Summary of the main findings. Graphic summary of the main findings presented in this manuscript.
