# Supplementary material for: Targeting ubiquitin-independent proteasome with small molecule increases susceptibility in pan-KRAS–mutant cancer models
Source: J Clin Invest. 2025 Mar 17;135(6):e185278. doi: 10.1172/JCI185278 (PMC11910216; doi:10.1172/JCI185278)

Full unedited blot/gel for Figure 4N-NEW

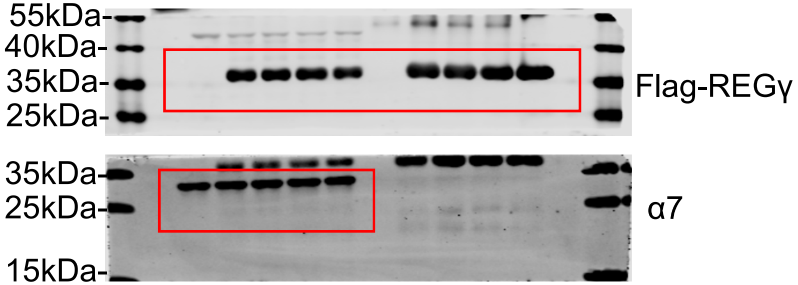

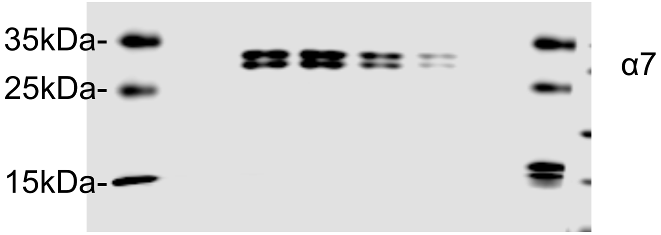

Full unedited blot/gel for Supplemental Figure 1 NEW

Supplemental Figure 1I

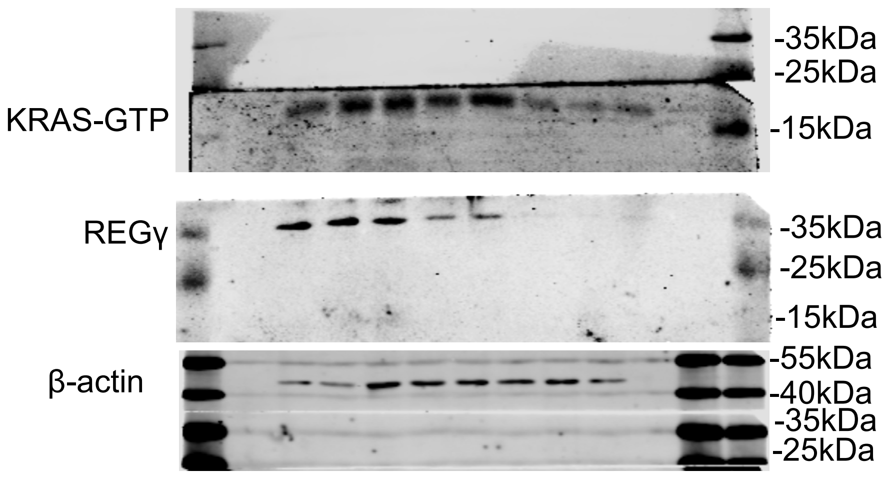

Supplemental Figure 1J

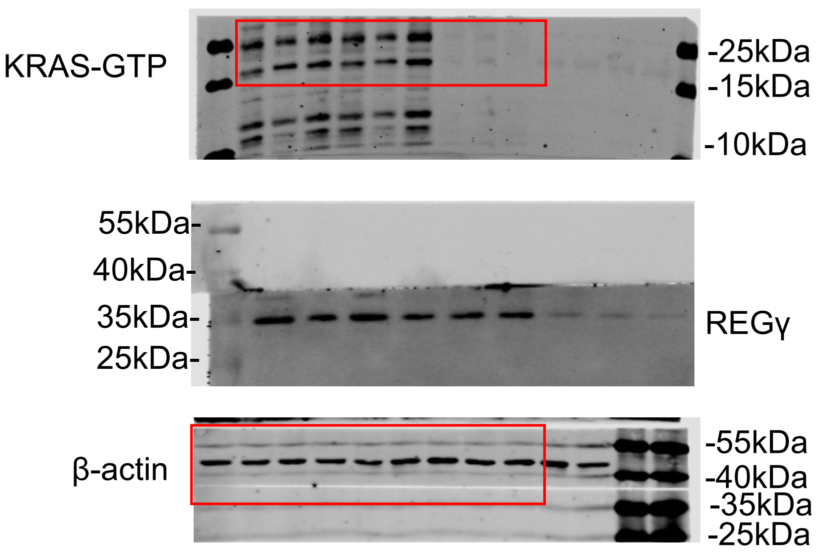

Supplemental Figure 1K

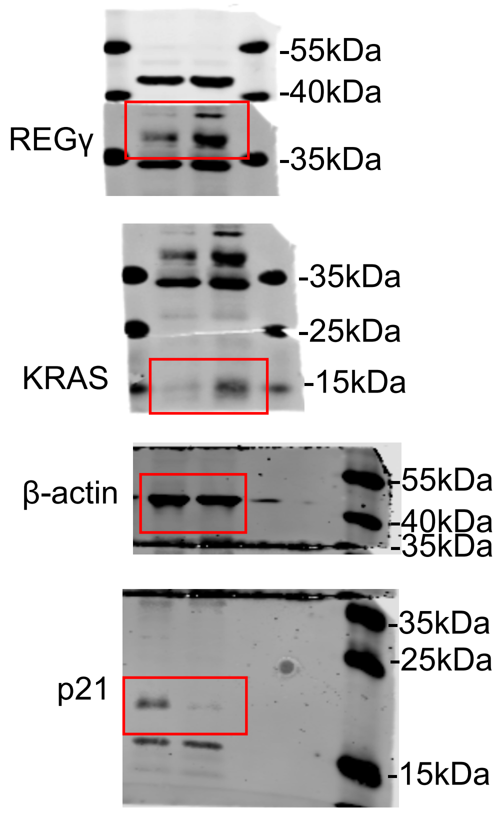

Full unedited blot/gel for Supplemental Figure 2

Supplemental Figure 2B NEW

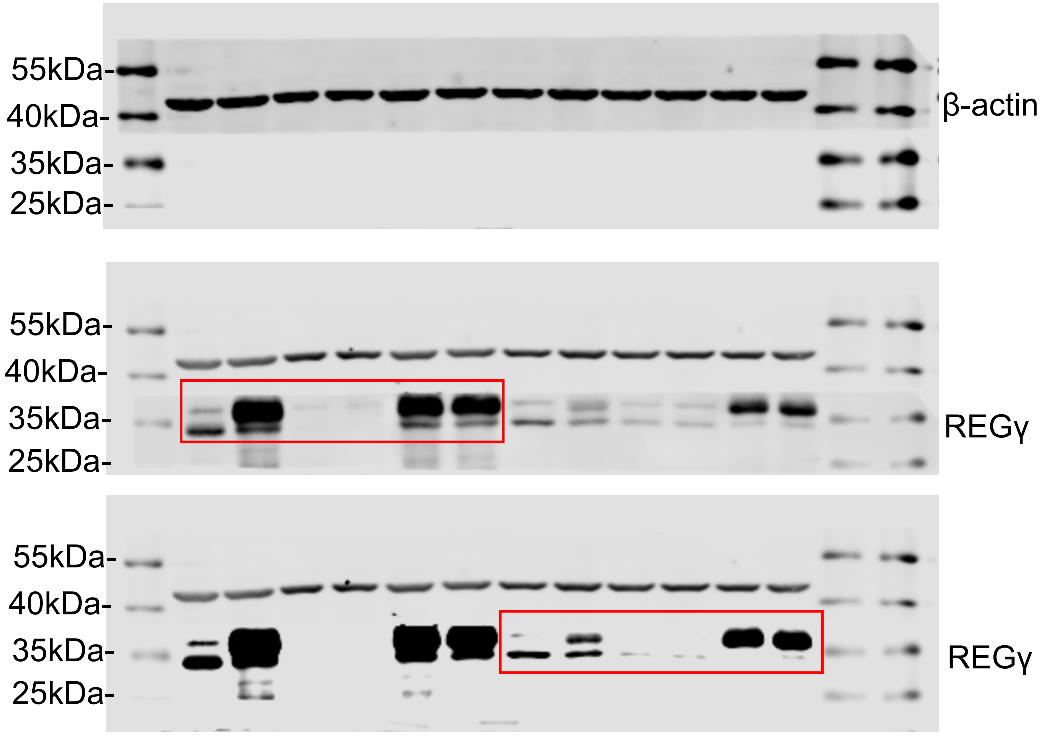

Full unedited blot/gel for Supplemental Figure 3A NEW

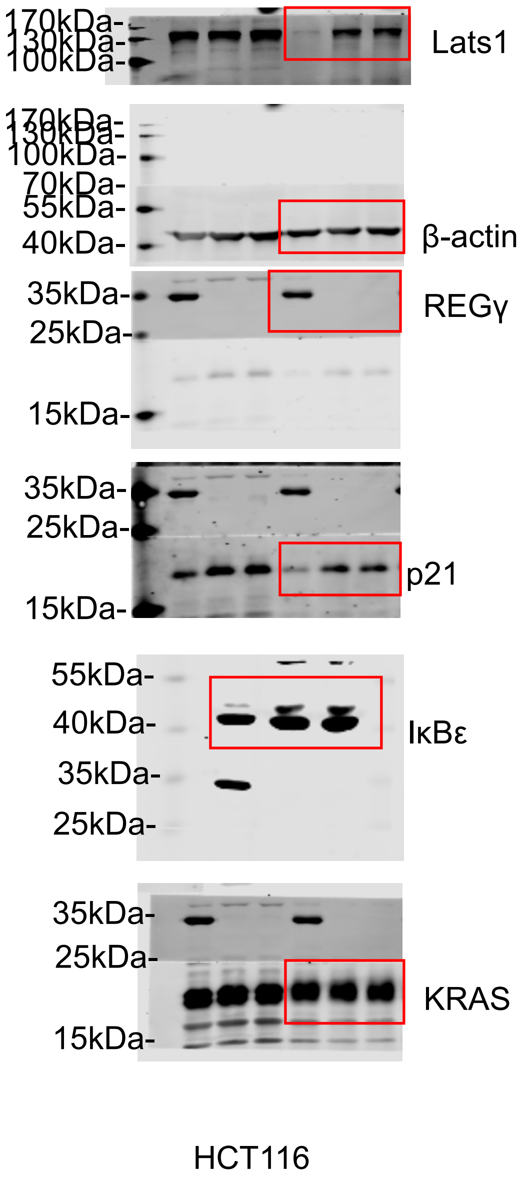

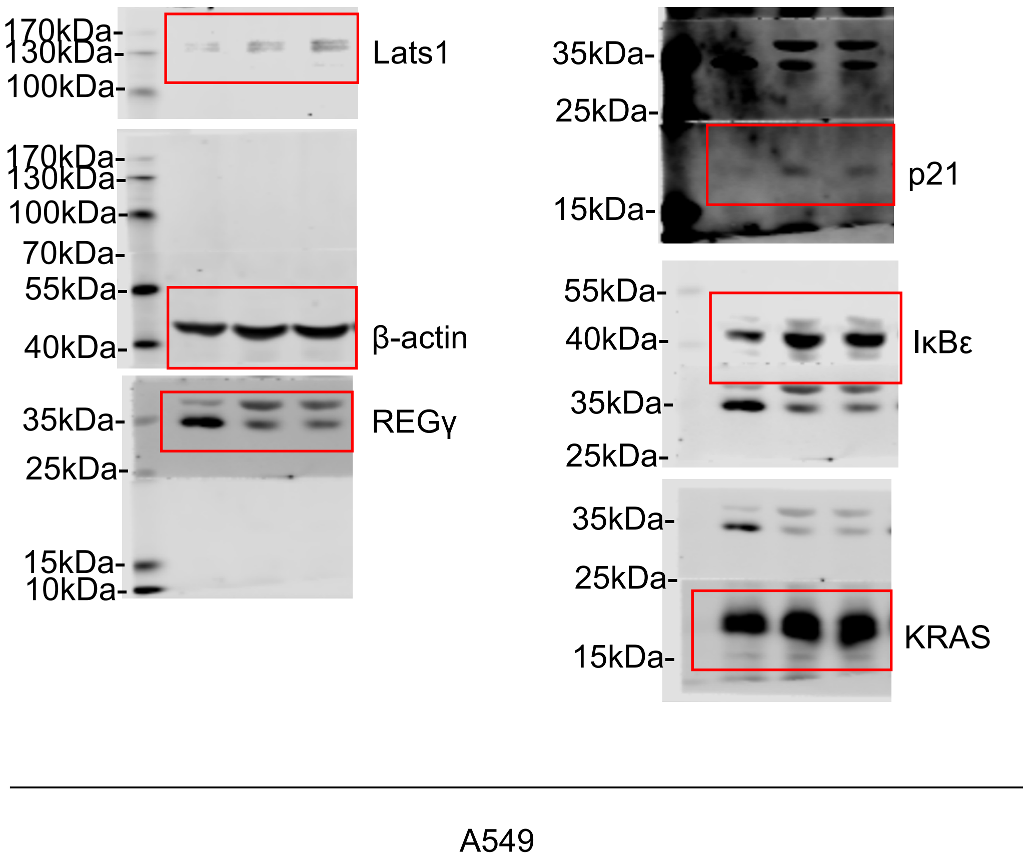

Full unedited blot/gel for Supplemental Figure 3C NEW

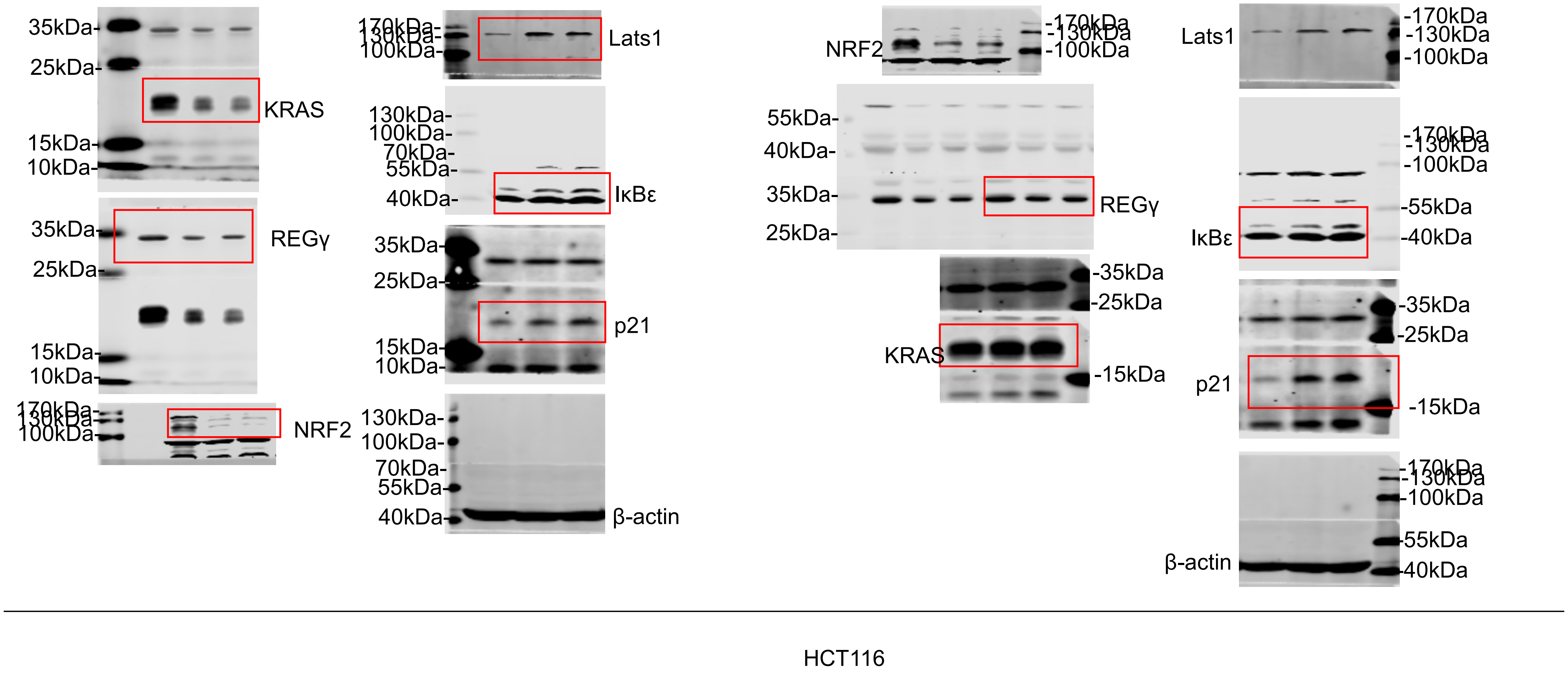

Full unedited blot/gel for Supplemental Figure 3C NEW

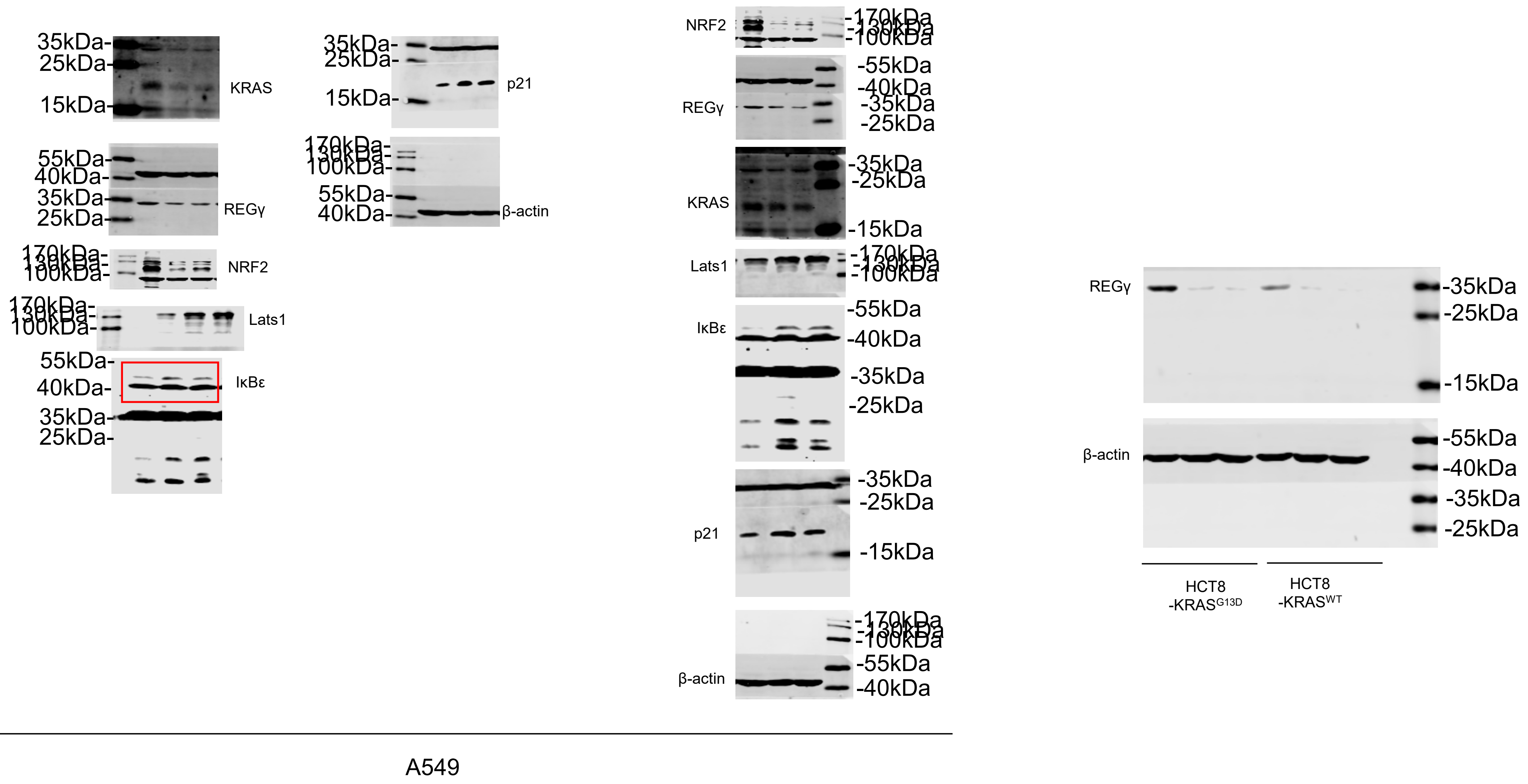

Full unedited blot/gel for Figure 1

Figure 1I

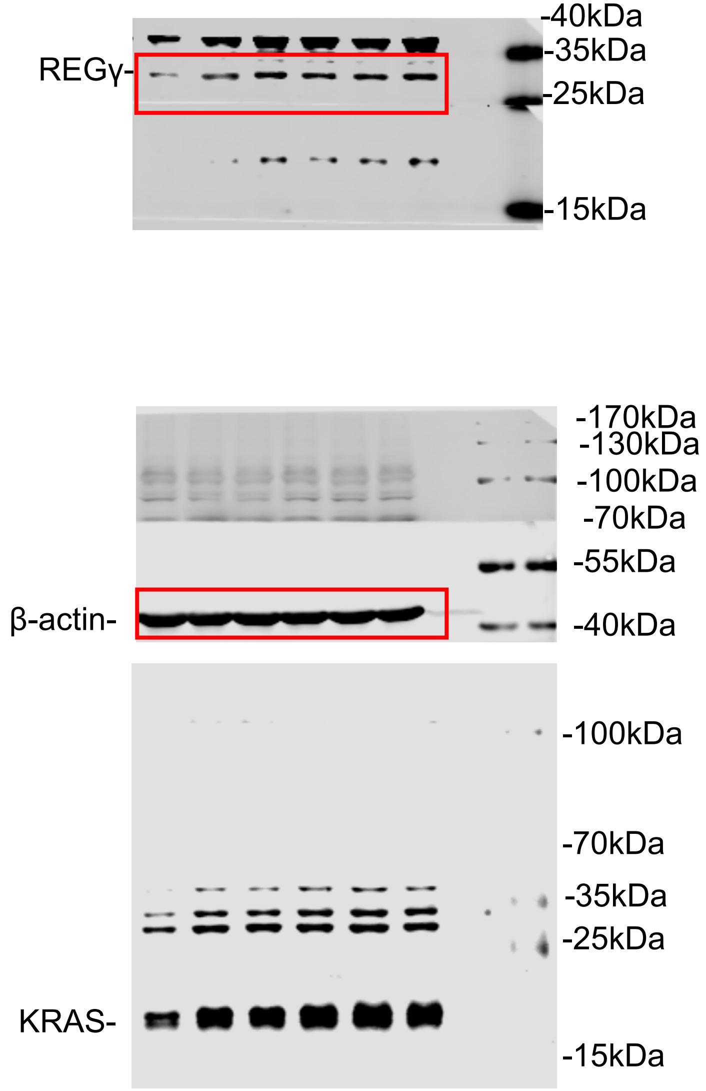

Figure 1G

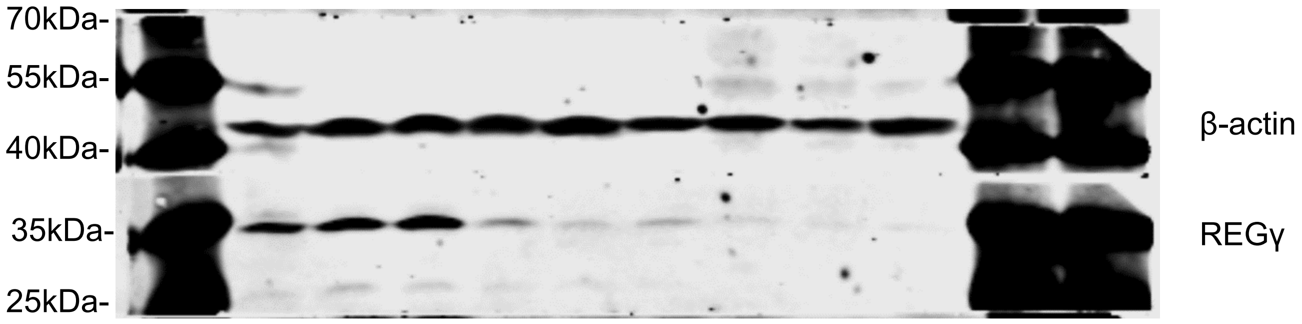

Full unedited blot/gel for Figure 3

Figure 3A

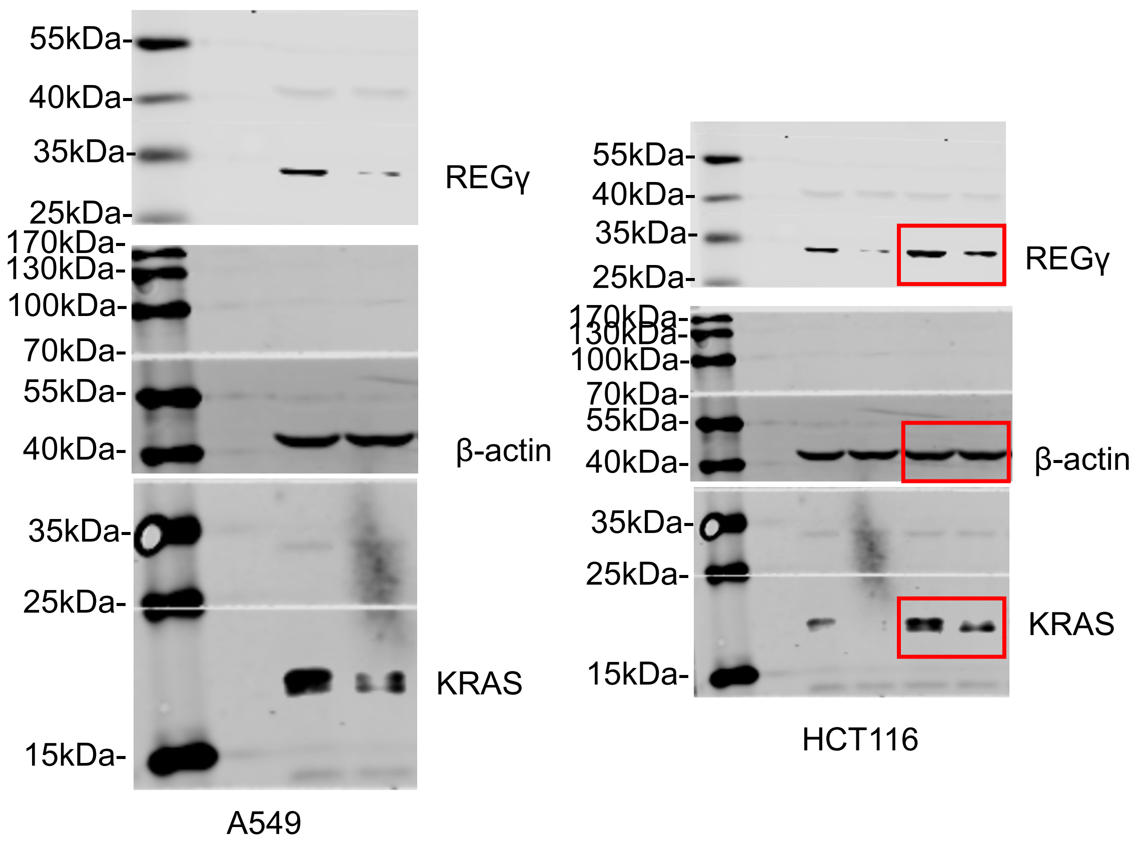

Full unedited blot/gel for Figure 3

Figure 3C

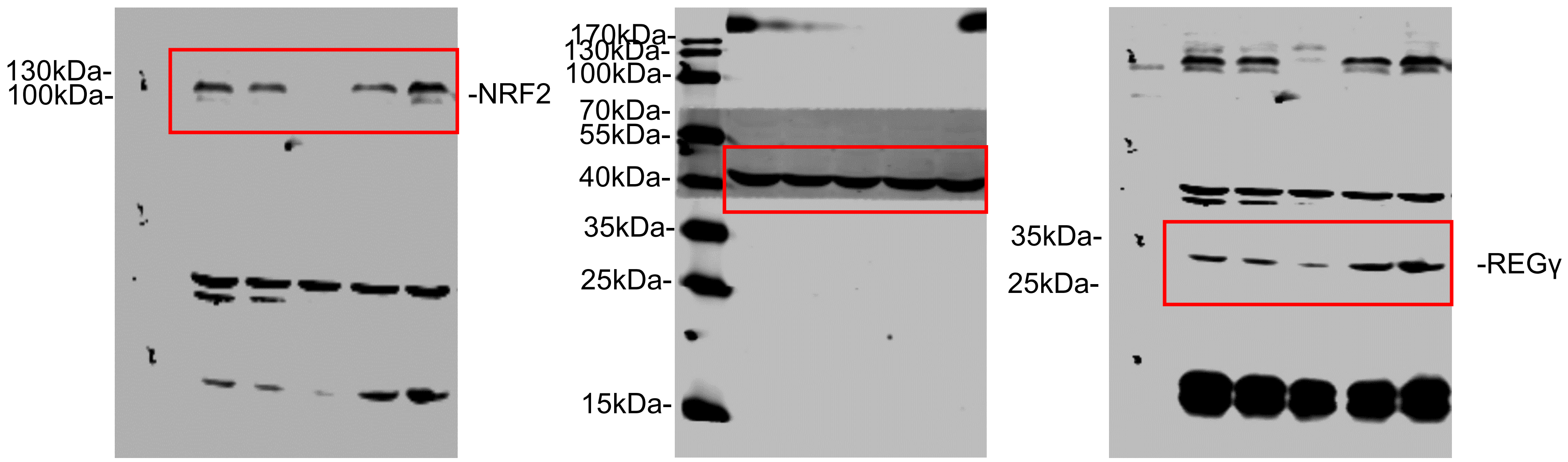

Figure 3D

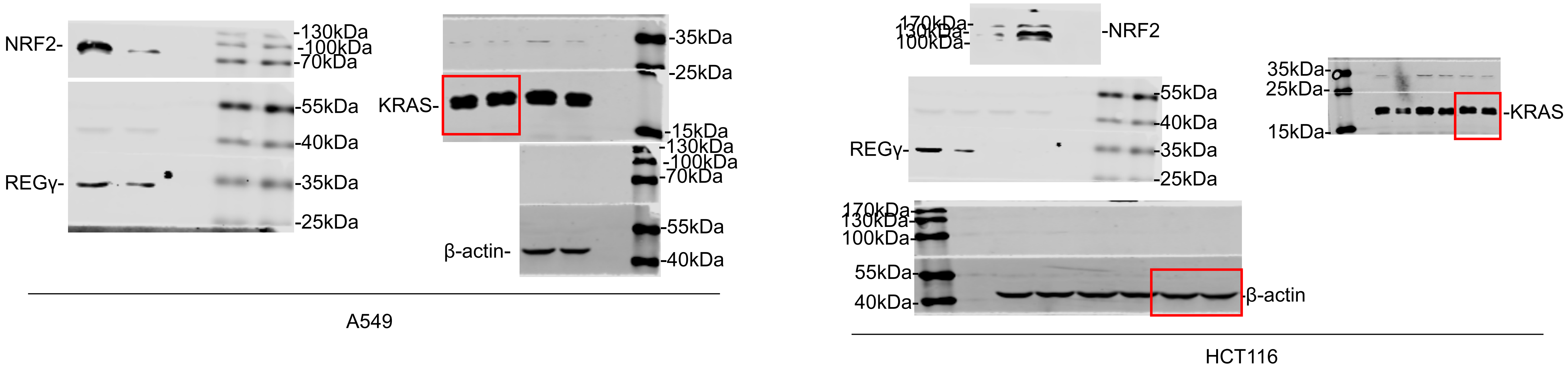

Full unedited blot/gel for Figure 4

Figure 4H

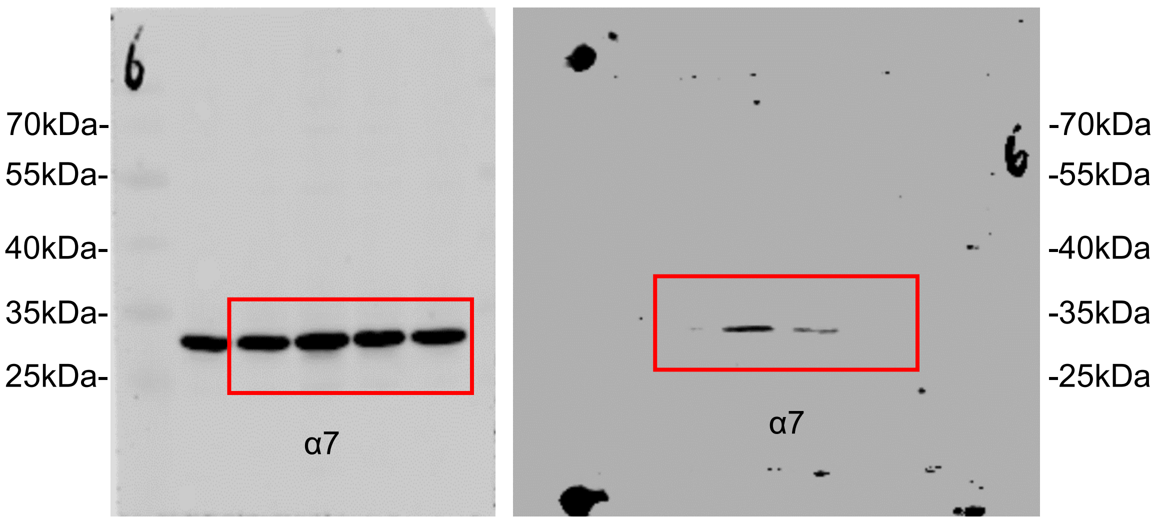

Figure 4I

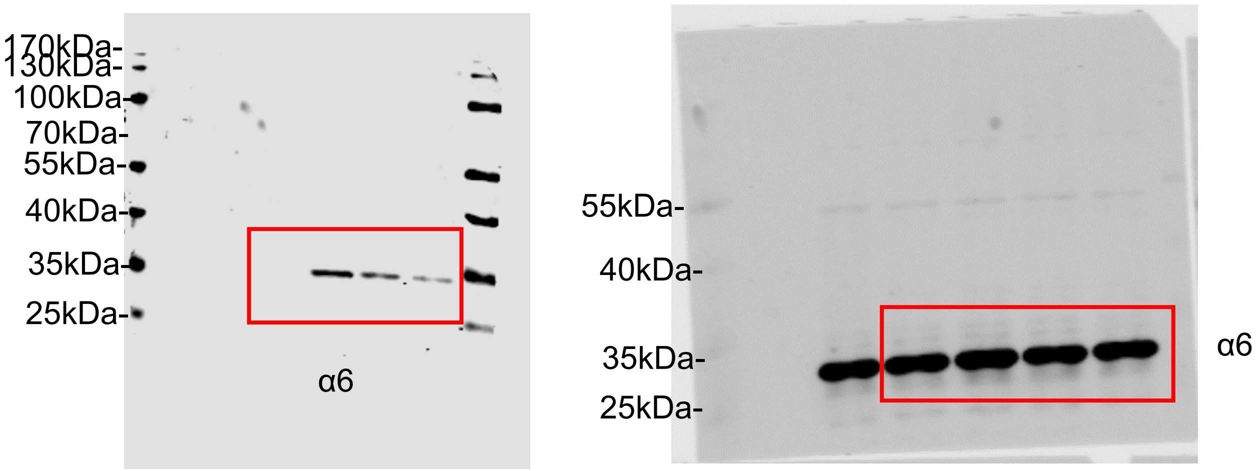

Full unedited blot/gel for Figure 4

Figure 4J

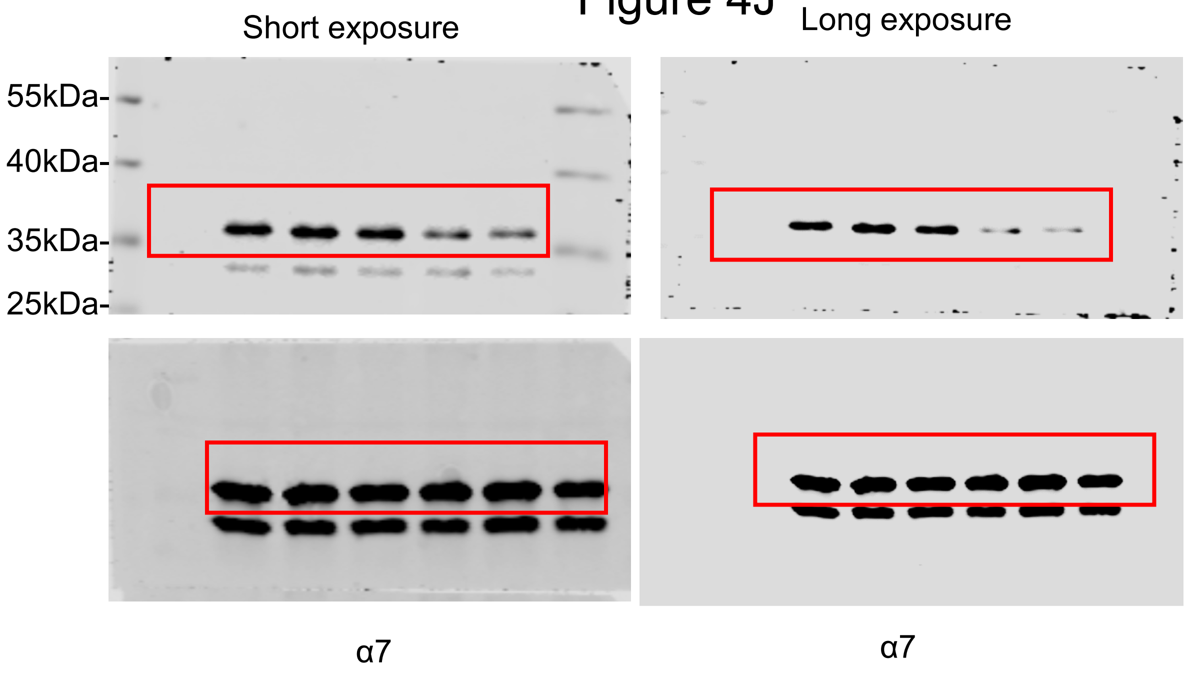

Figure 4K

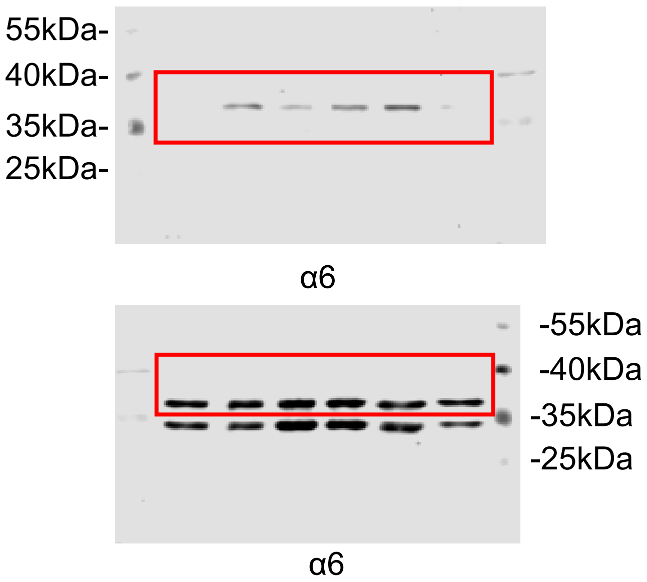

Figure 4M

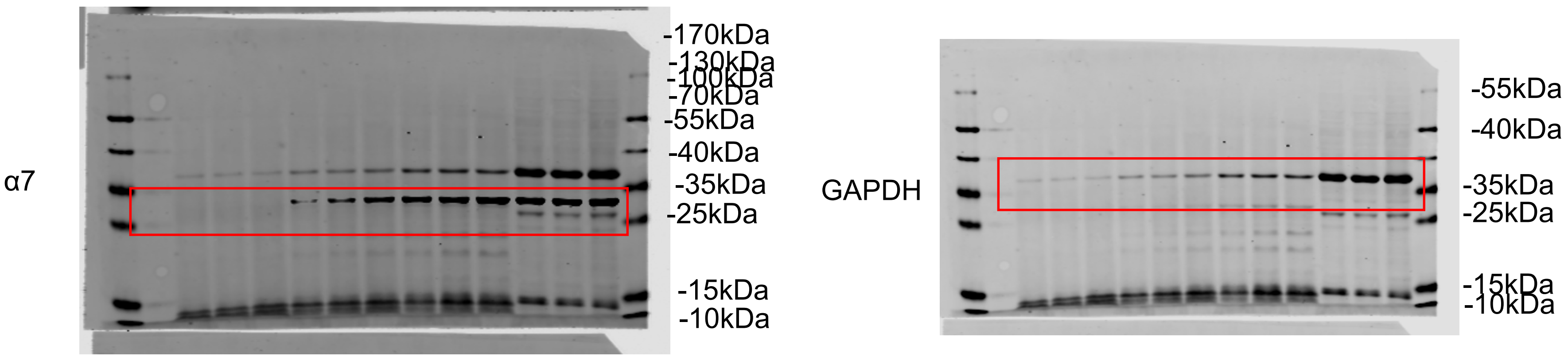

Full unedited blot/gel for Figure 4N

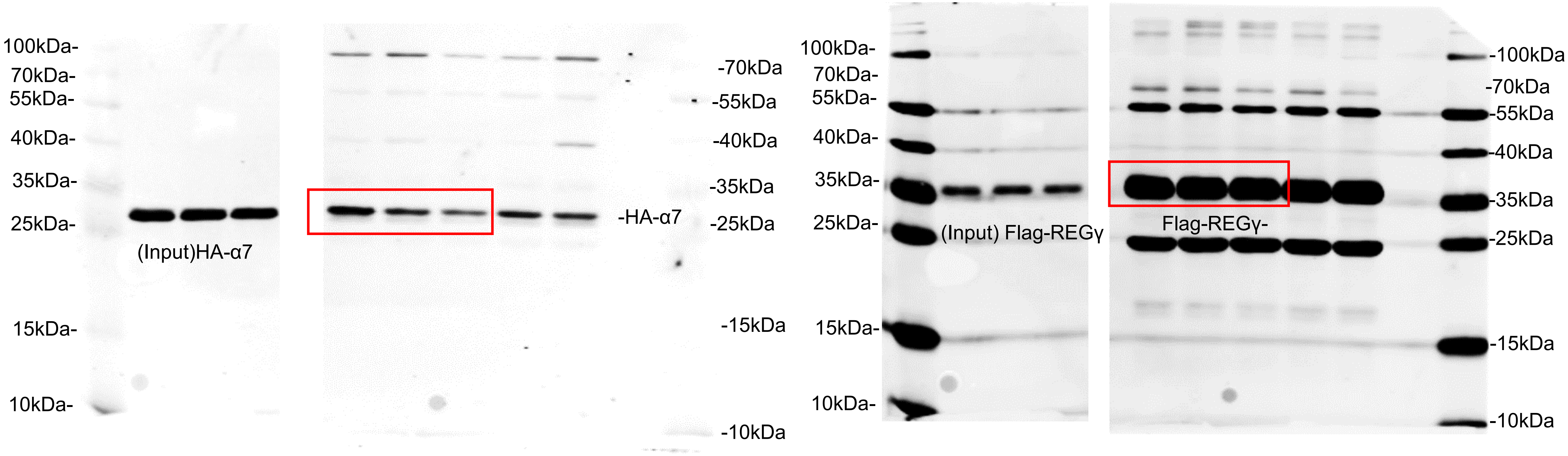

Full unedited blot/gel for Figure 5B

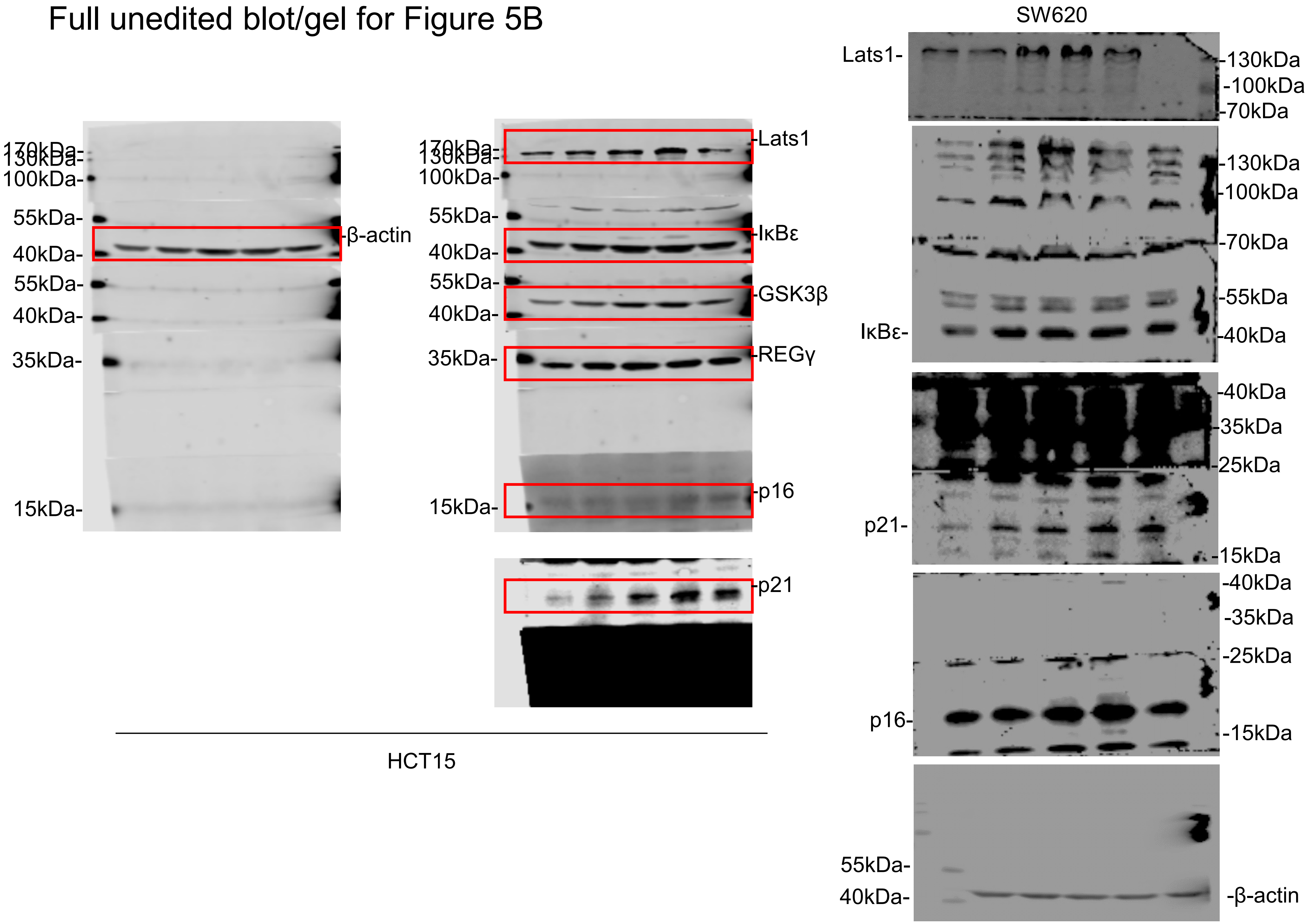

Full unedited blot/gel for Figure 5

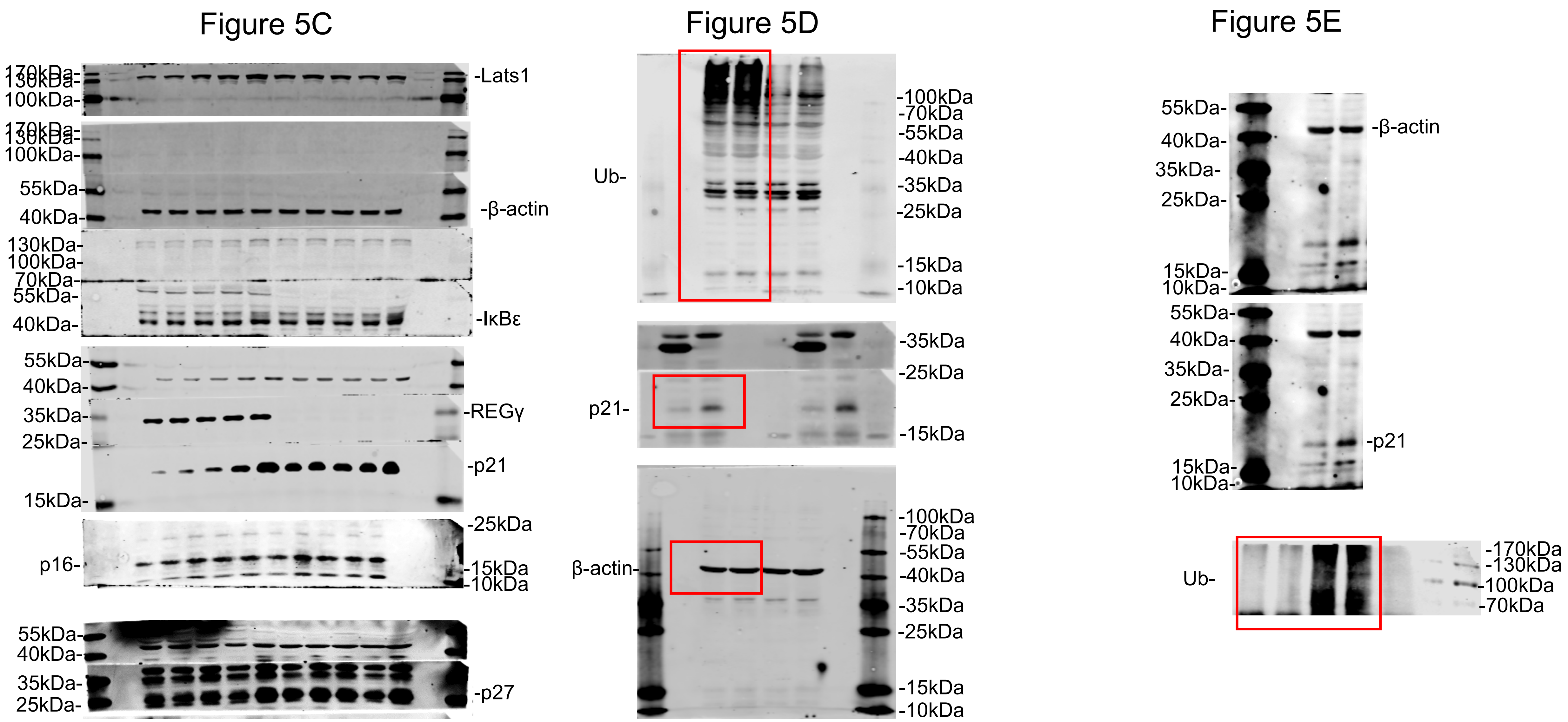

Full unedited blot/gel for Figure 6

Figure 6E

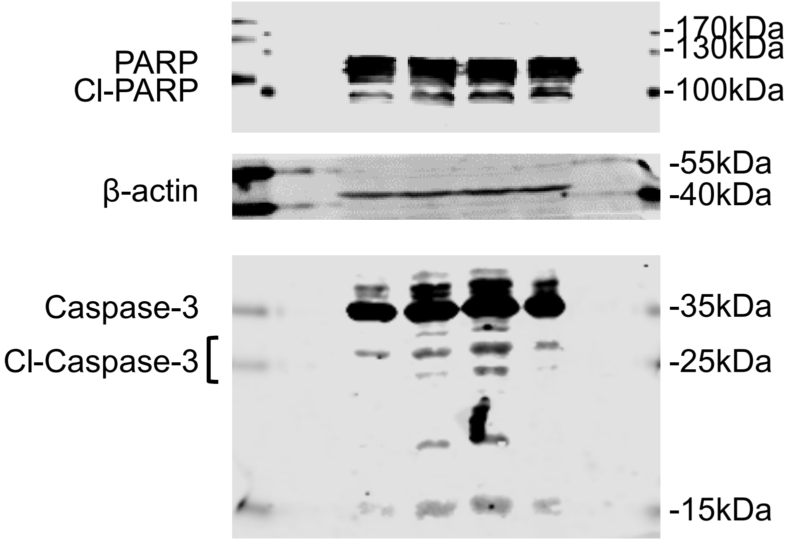

HCT15

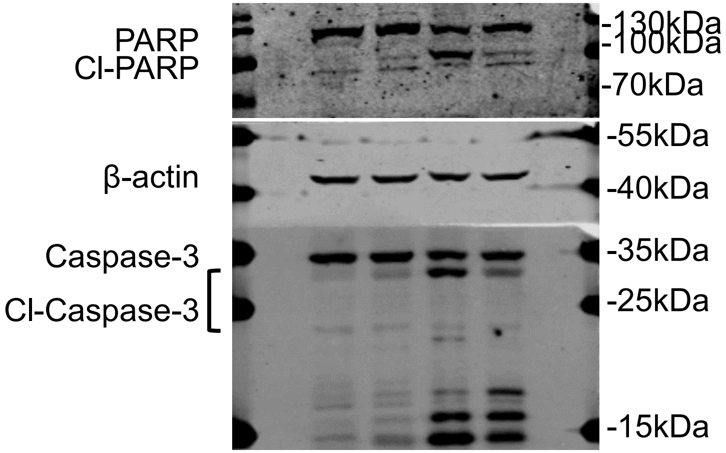

A549

Figure 6F

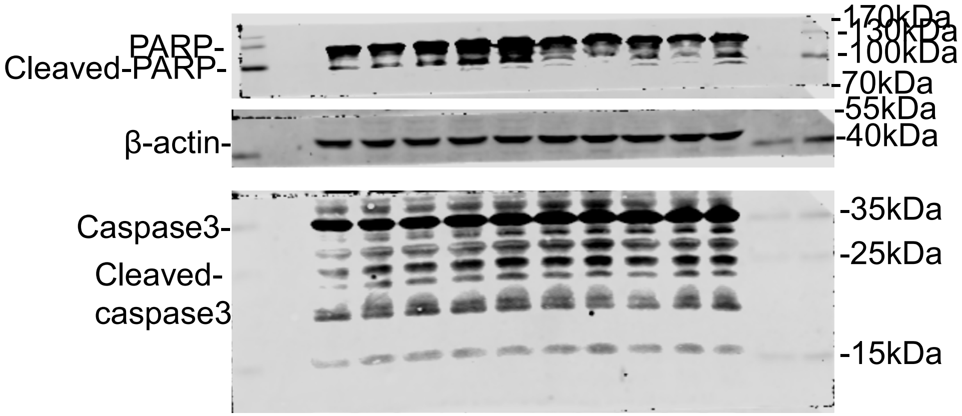

Full unedited blot/gel for Figure 7F

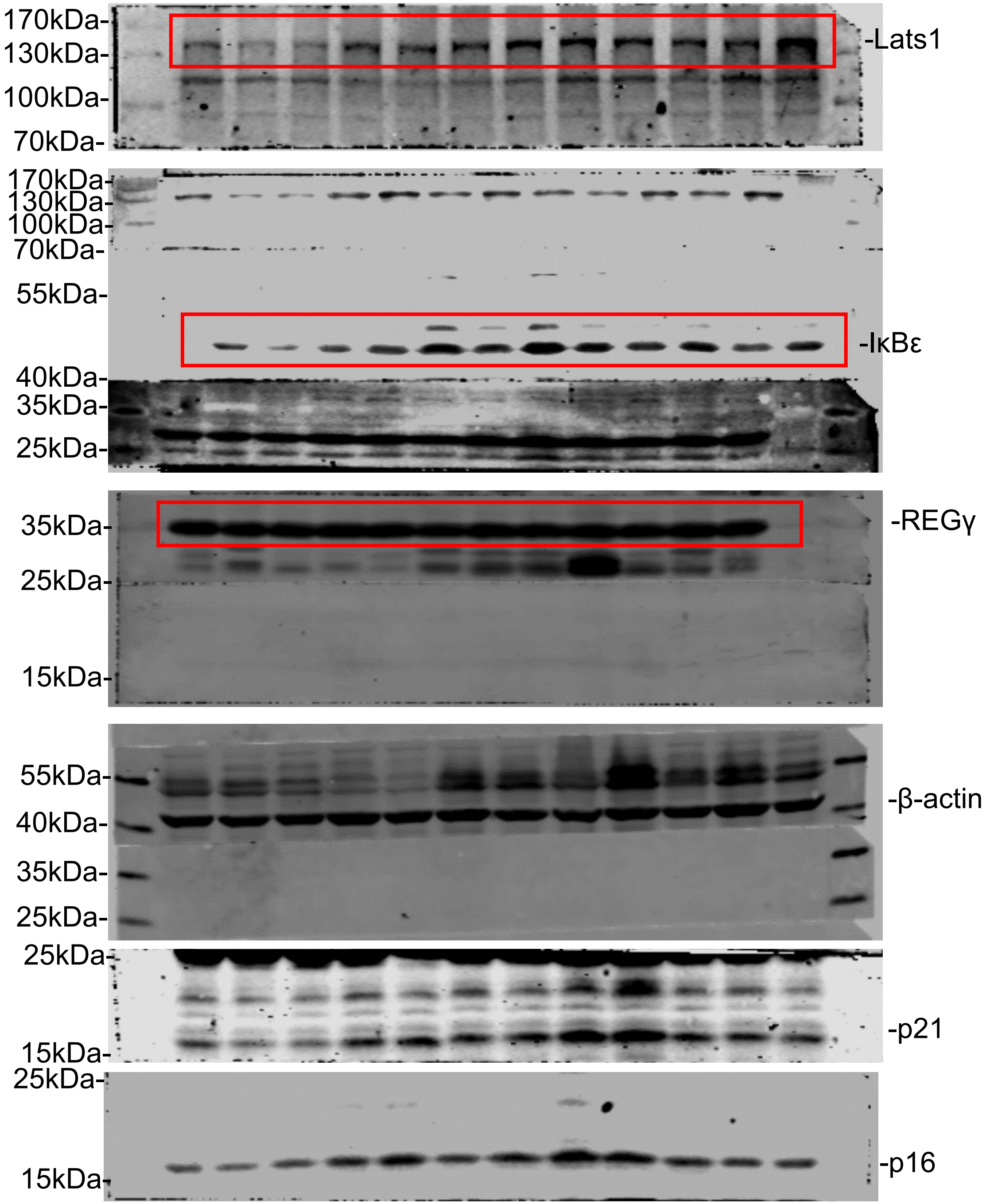

Full unedited blot/gel for Supplemental Figure 1 D

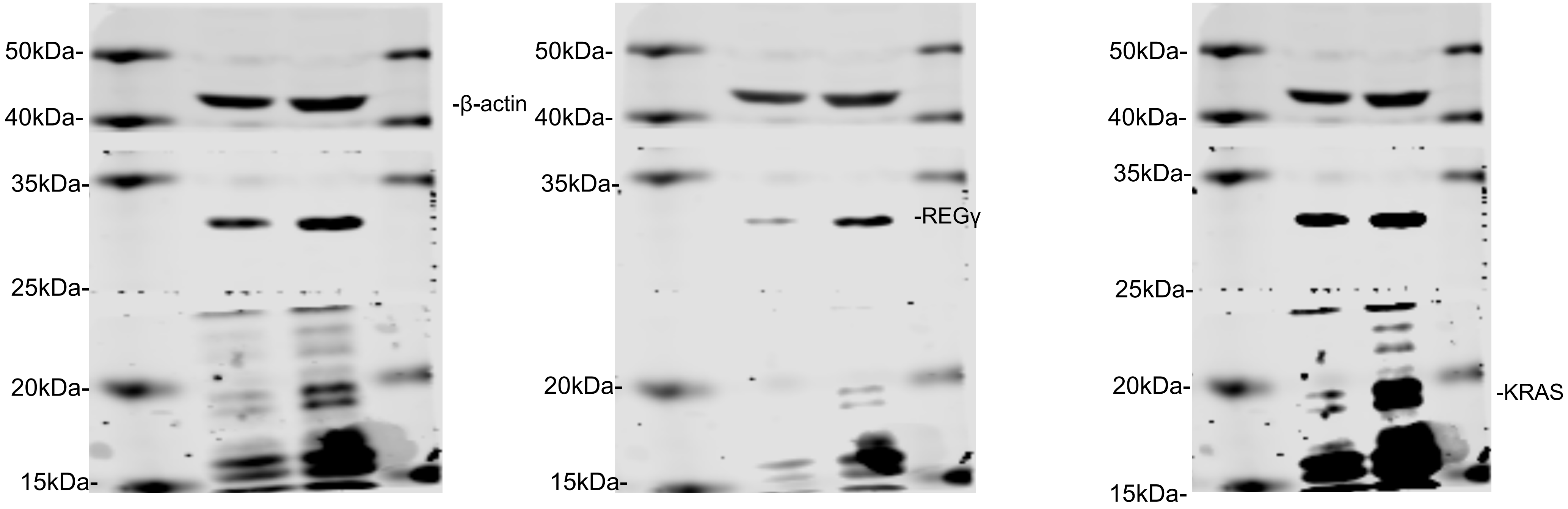

Full unedited blot/gel for Supplemental Figure 1 E and F

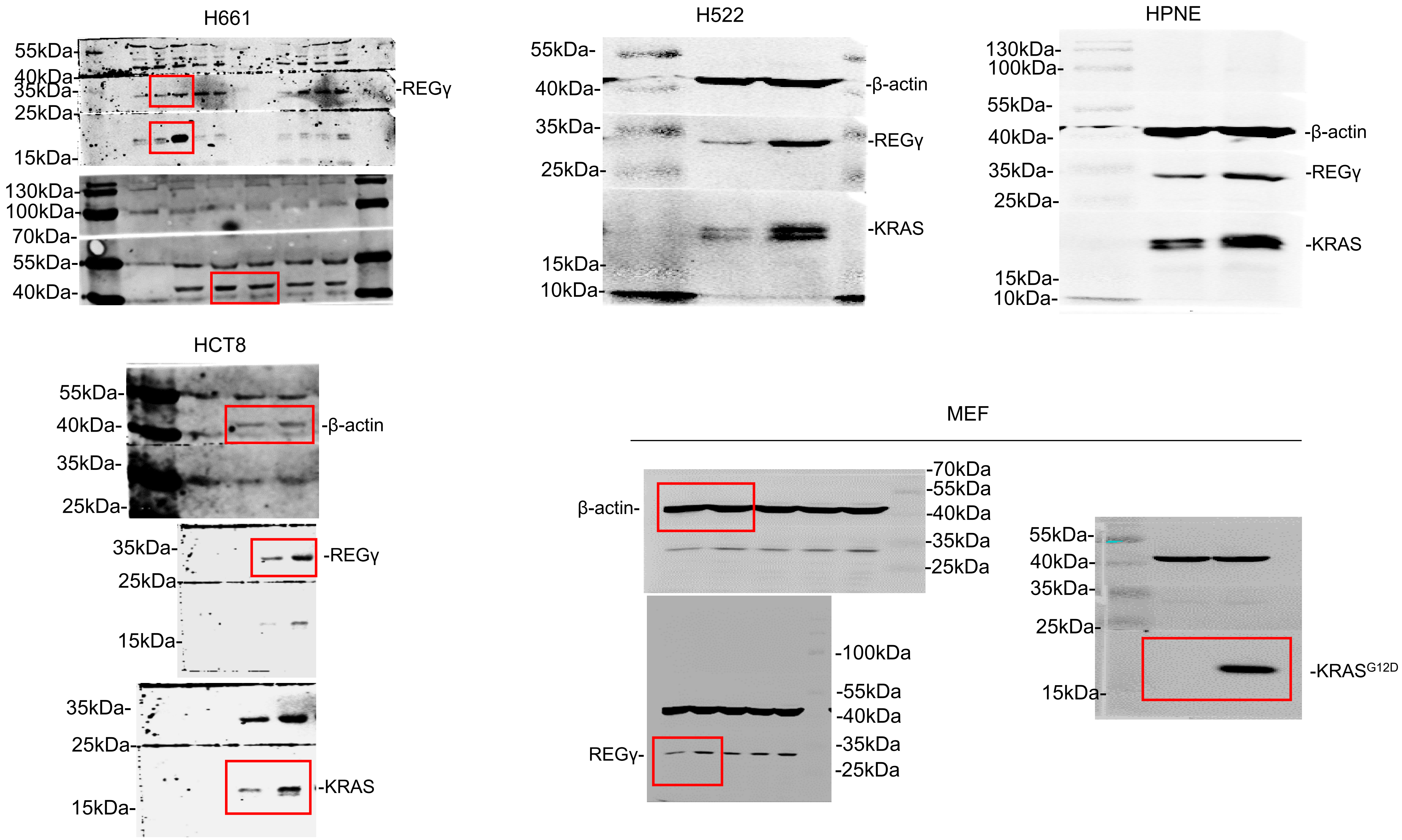

Full unedited blot/gel for Supplemental Figure 1G

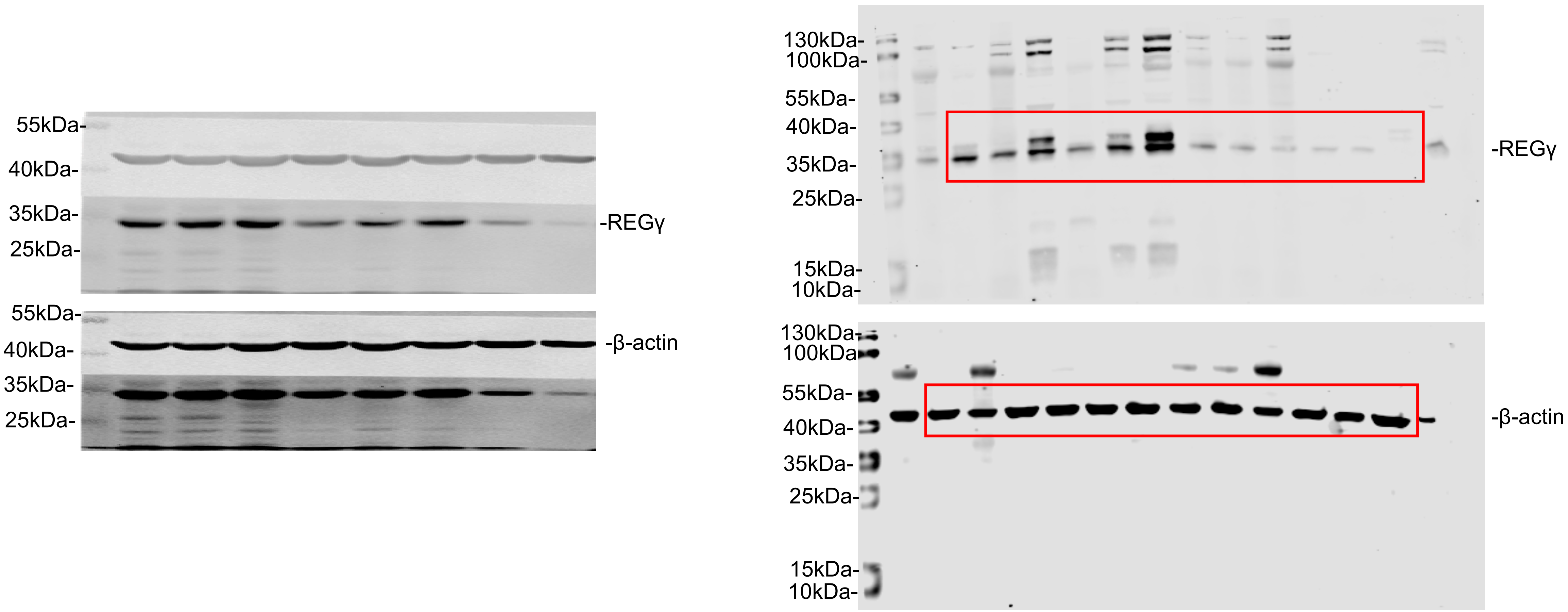

Full unedited blot/gel for Supplemental Figure 2

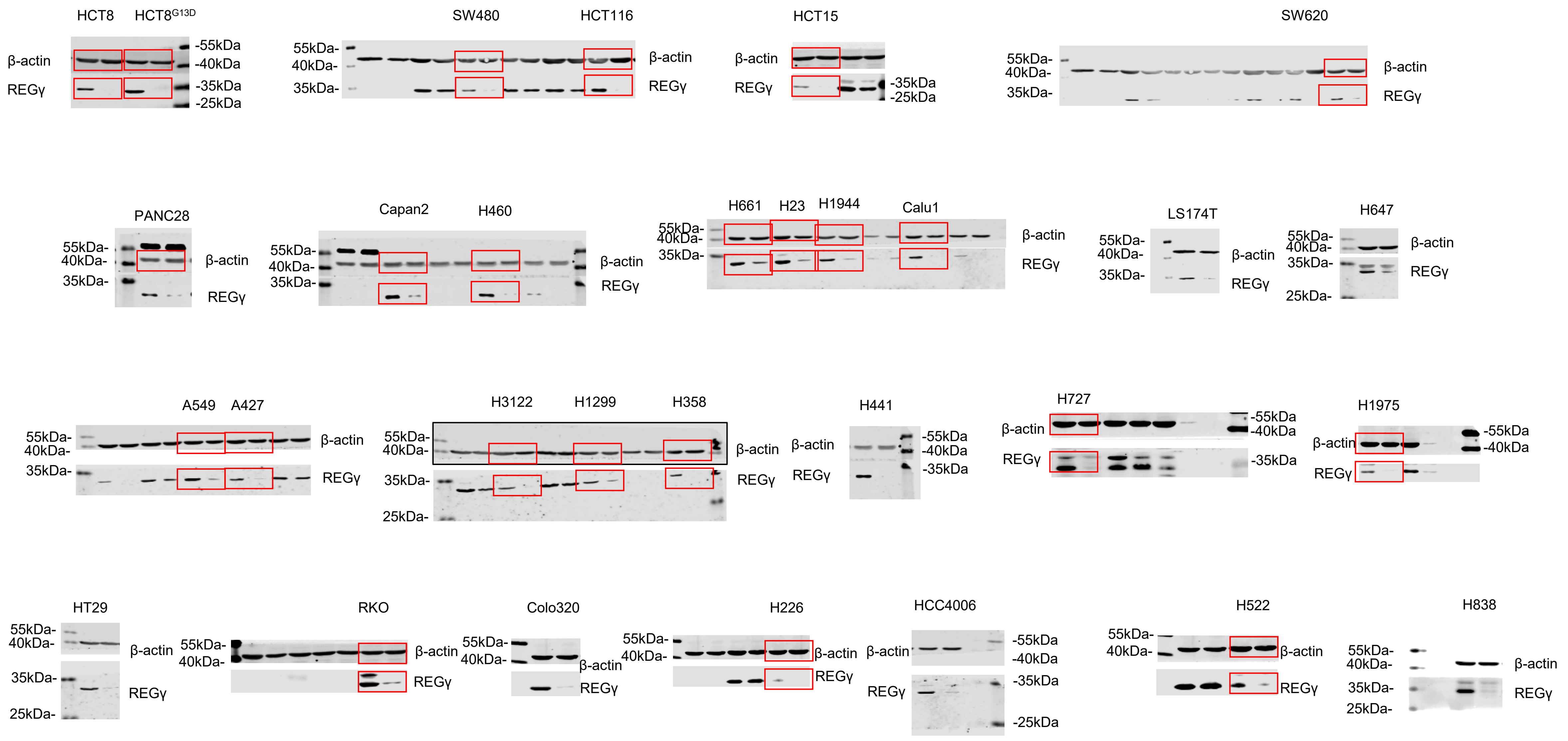

Full unedited blot/gel for Supplemental Figure 3A

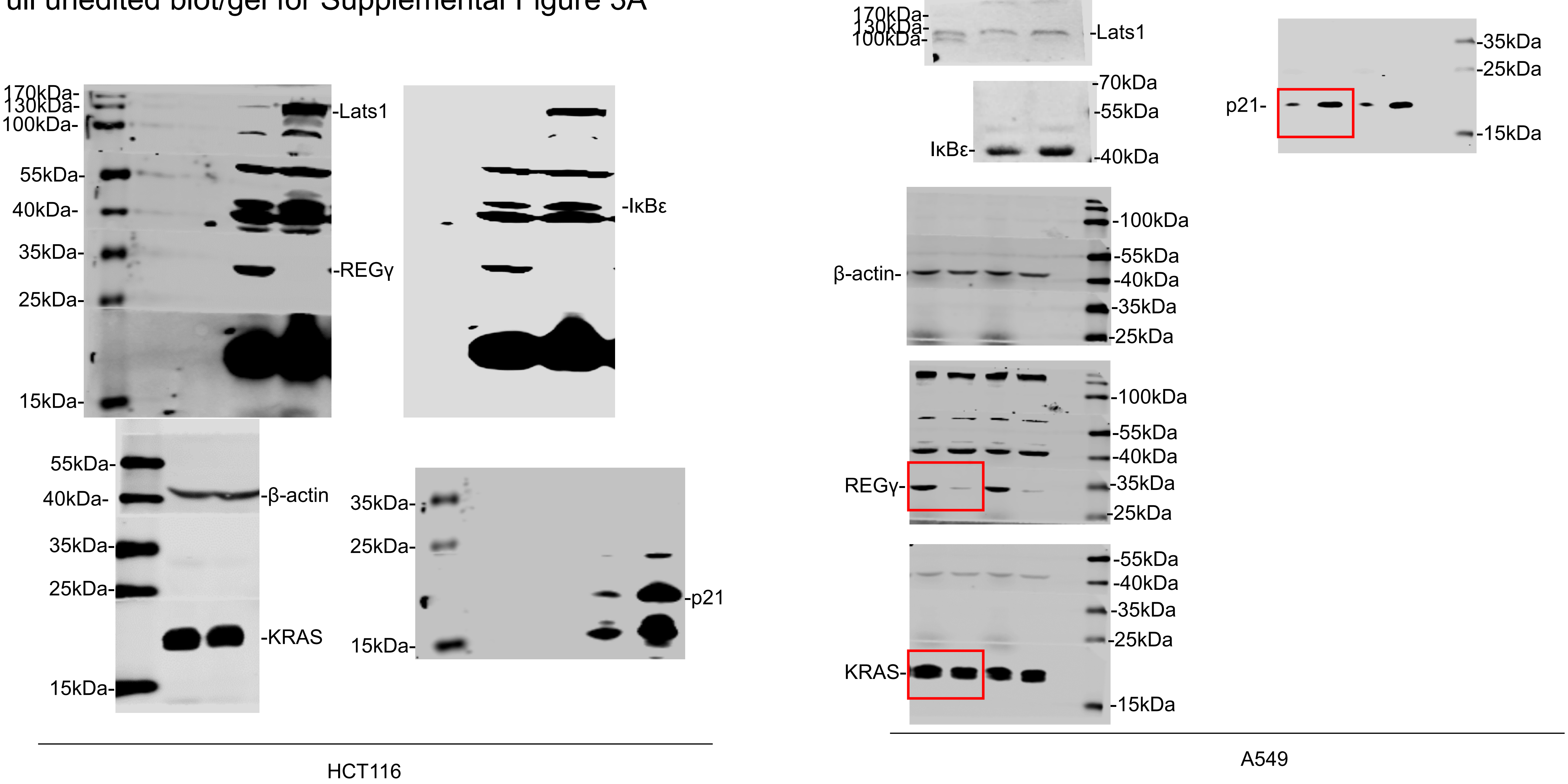

Full unedited blot/gel for Supplemental Figure 4B

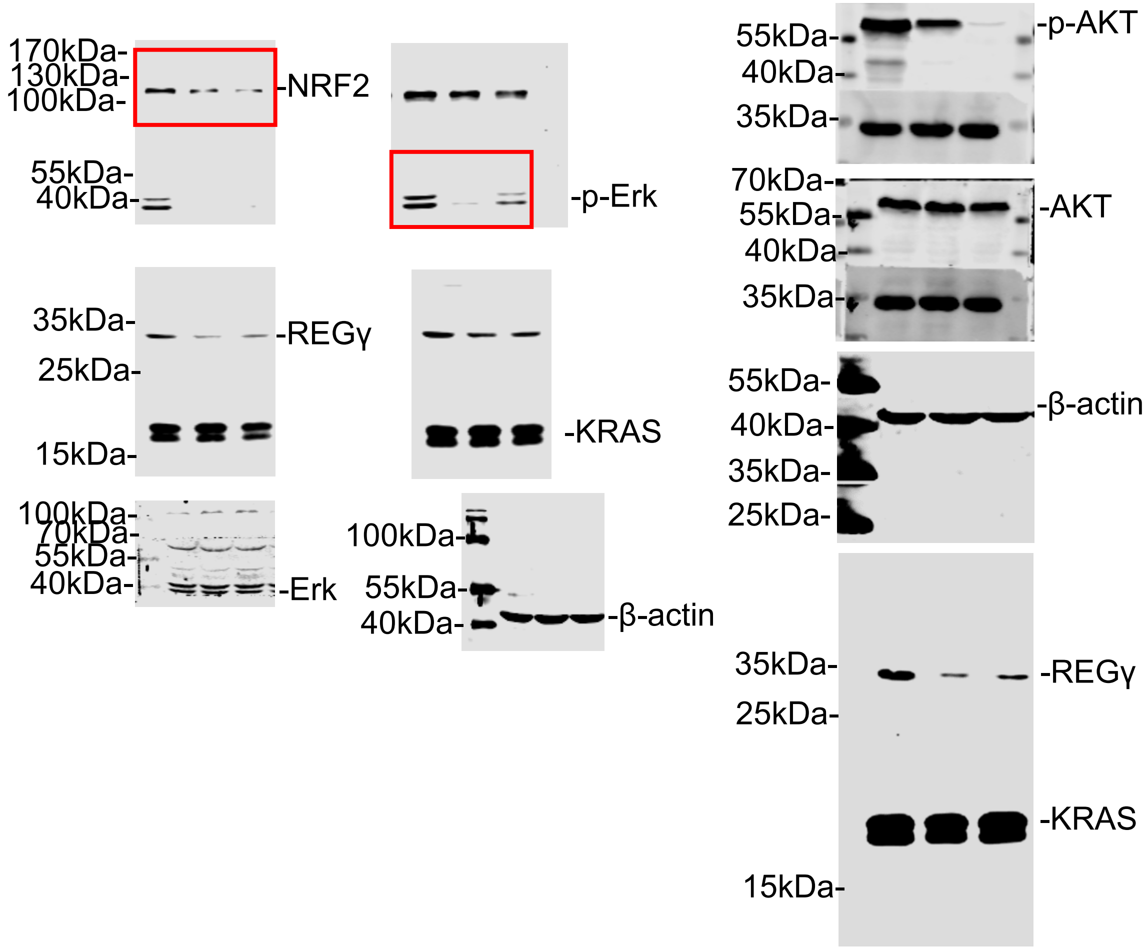

HCT8-KRAS^{G13D}

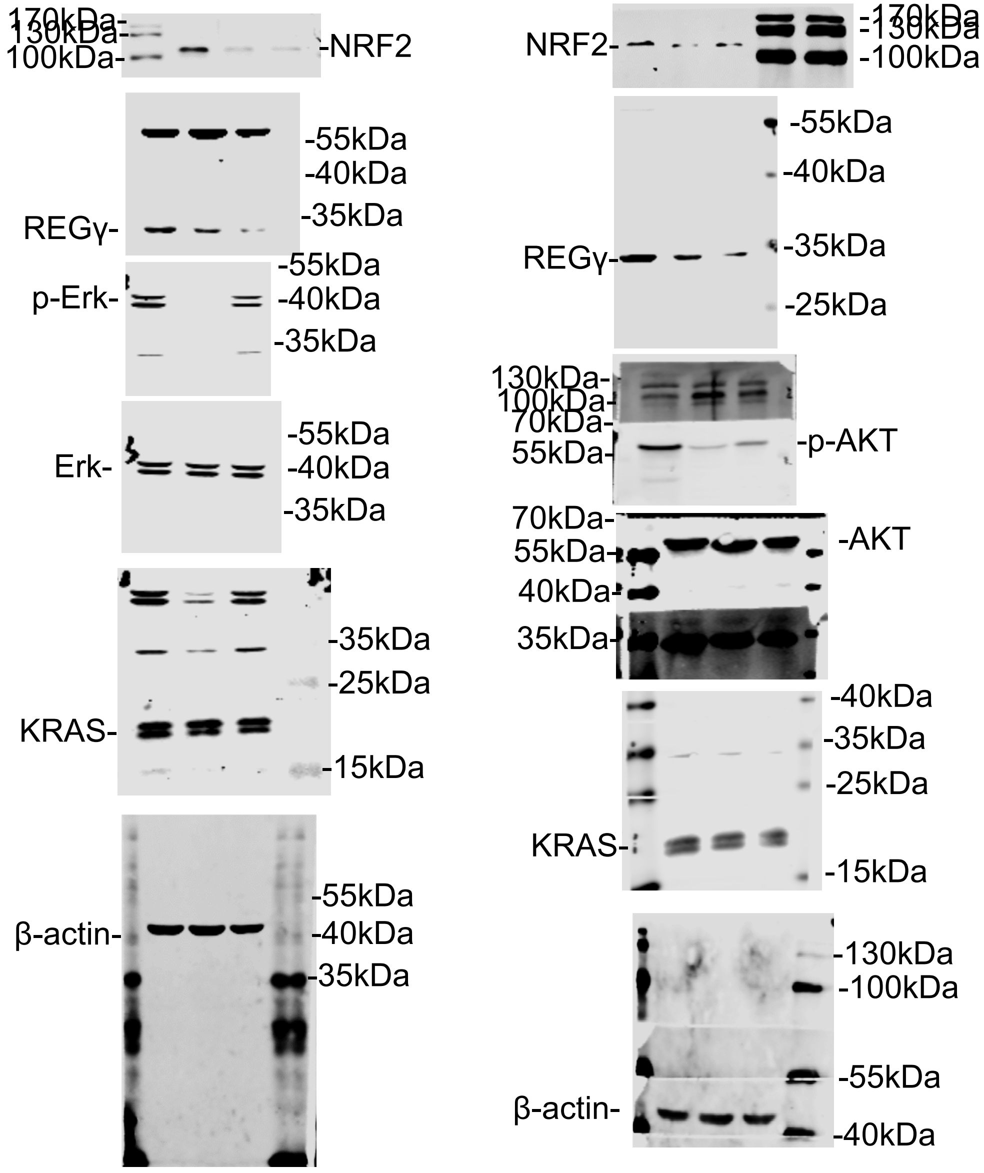

A549(KRAS^{G12S})

Full unedited blot/gel for Supplemental Figure 4B

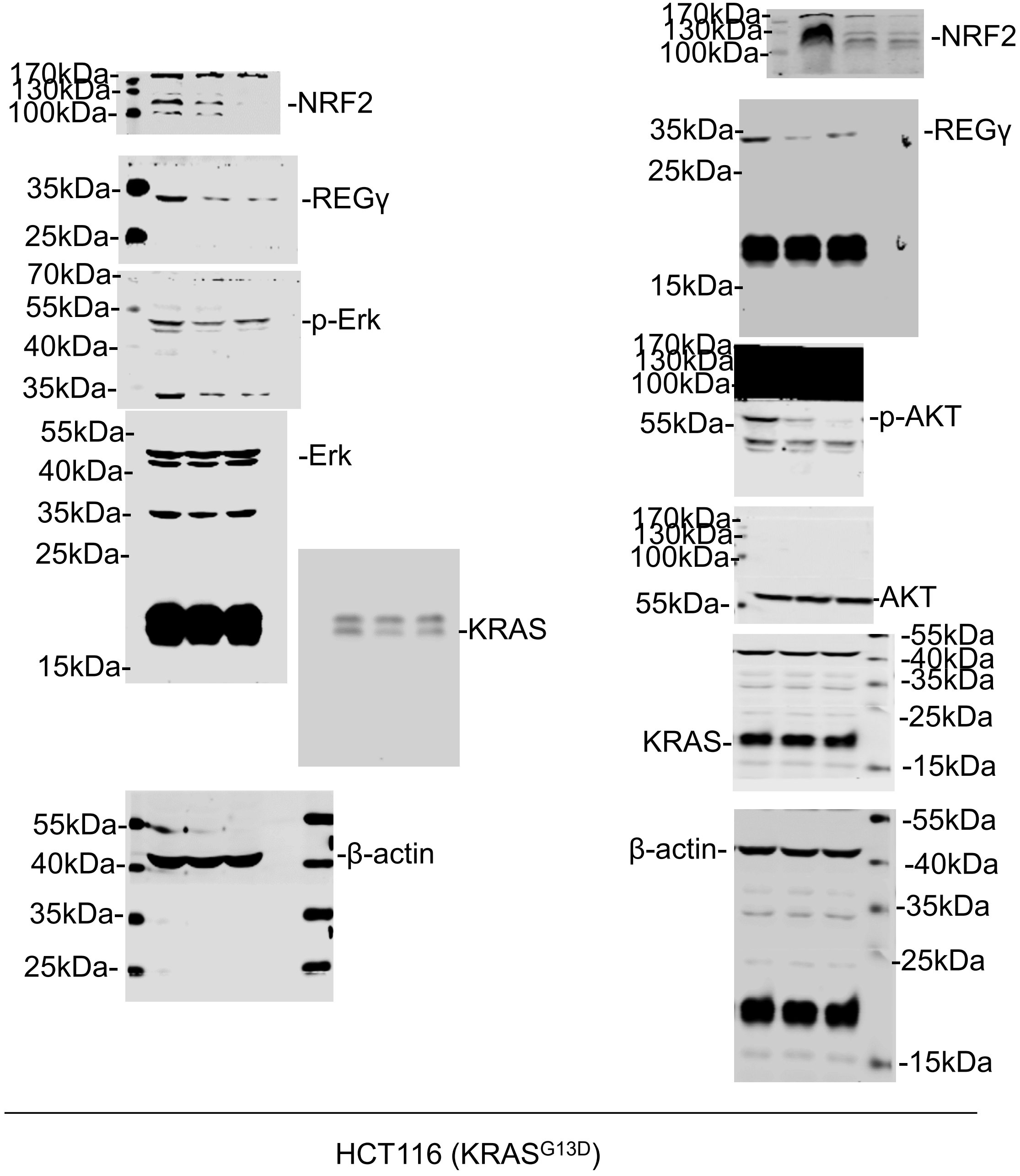

Full unedited blot/gel for
Supplemental Figure 4C and D

Supplemental Figure 4C

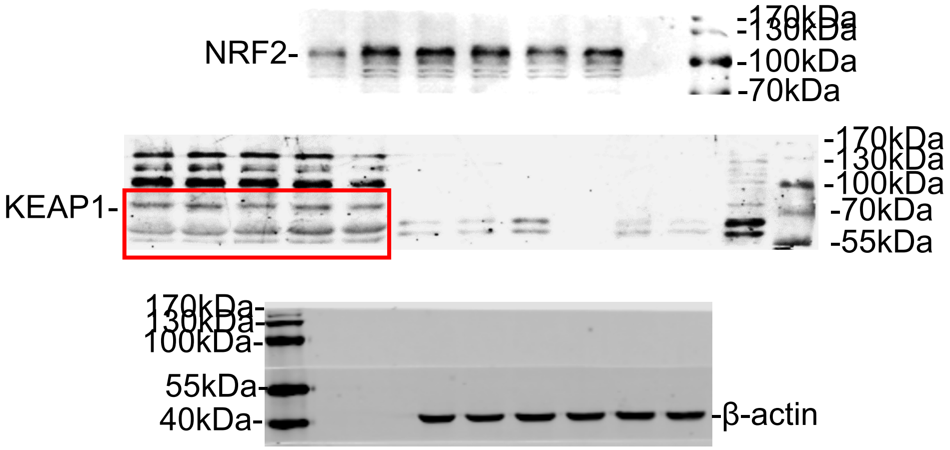

Supplemental Figure 4D

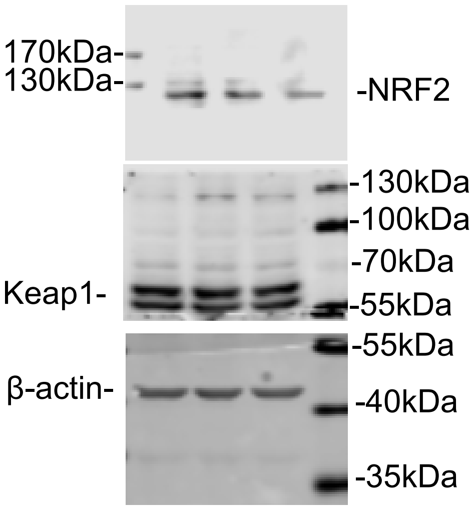

Full unedited blot/gel for Supplemental Figure 5A

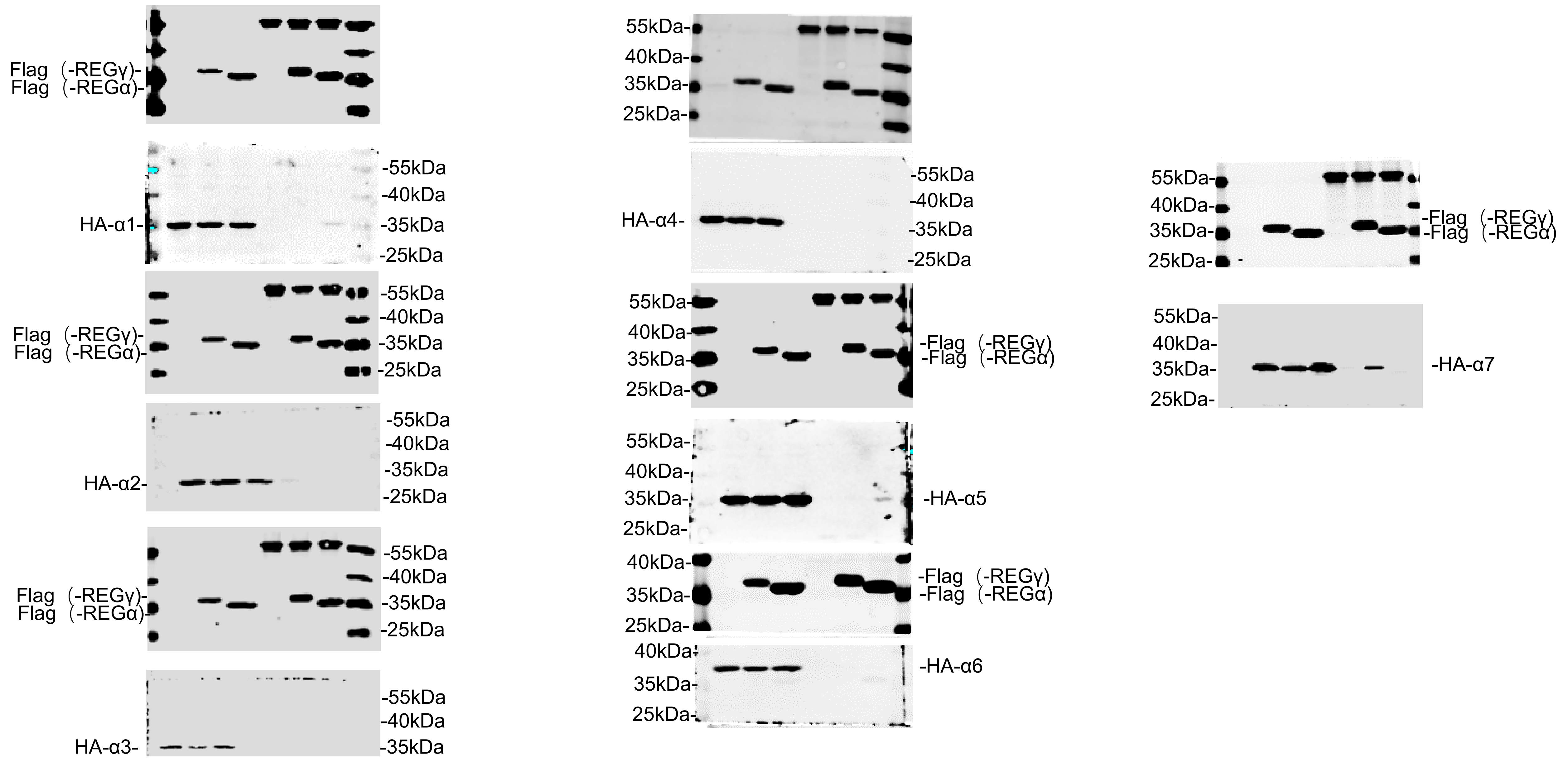

Full unedited blot/gel for Supplemental Figure 5B

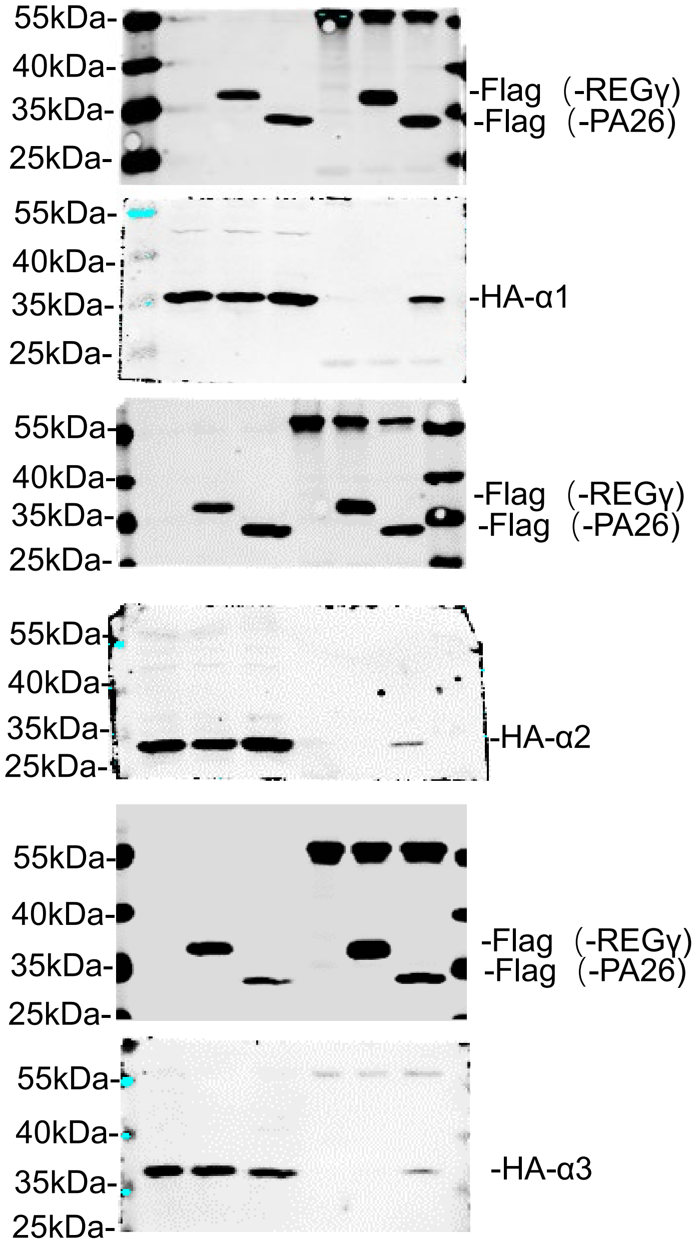

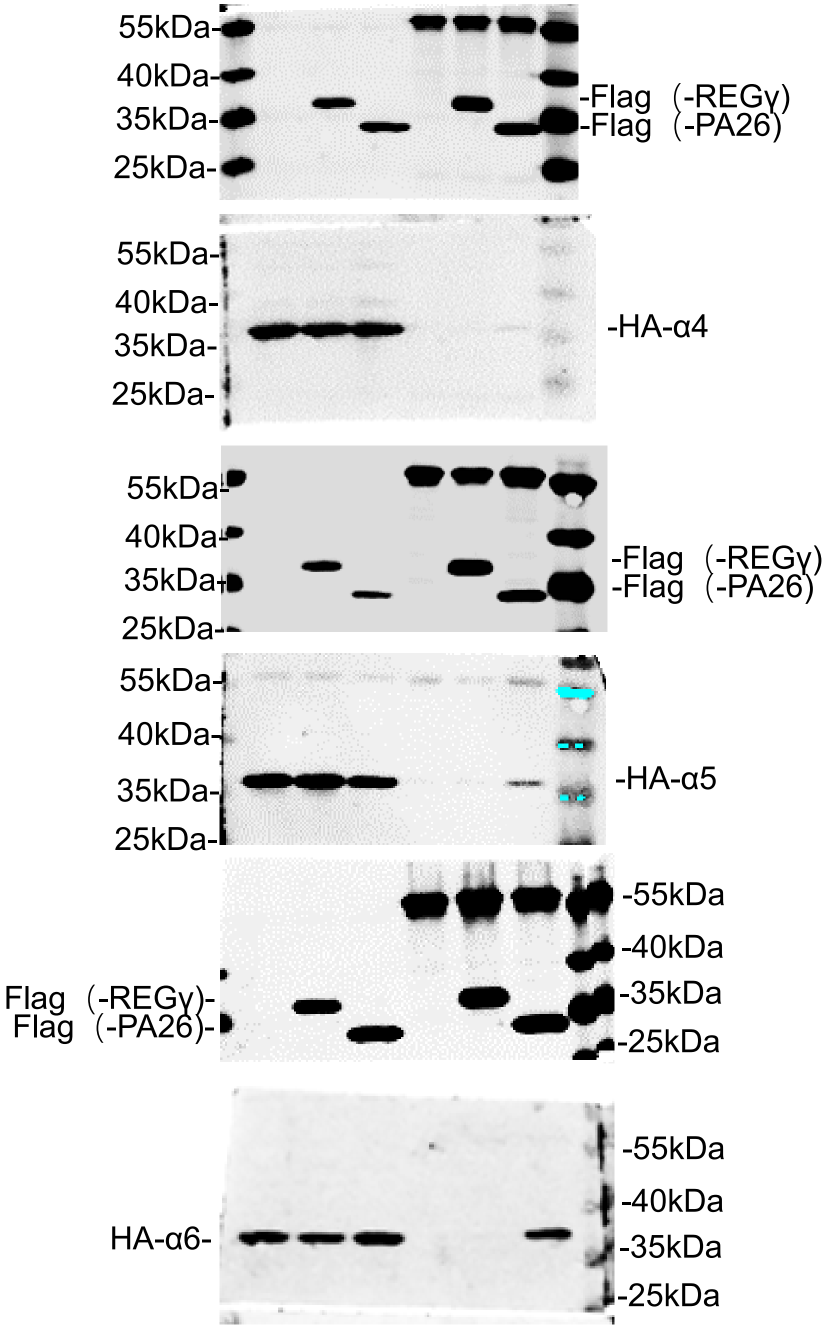

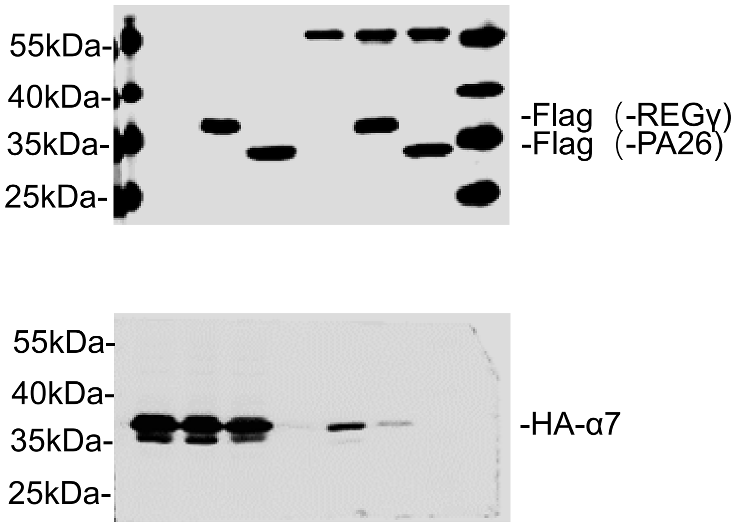

Full unedited blot/gel for Supplemental Figure 5D

HA-α7 Input

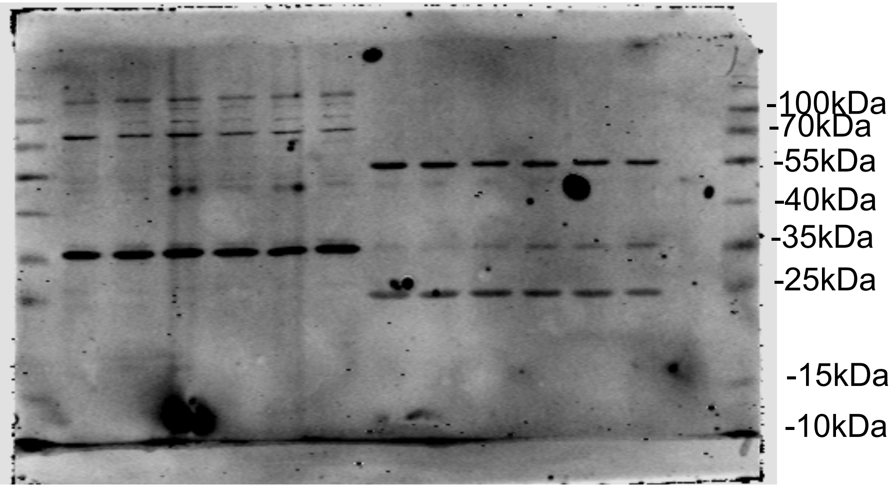

Flag-REGγ Input

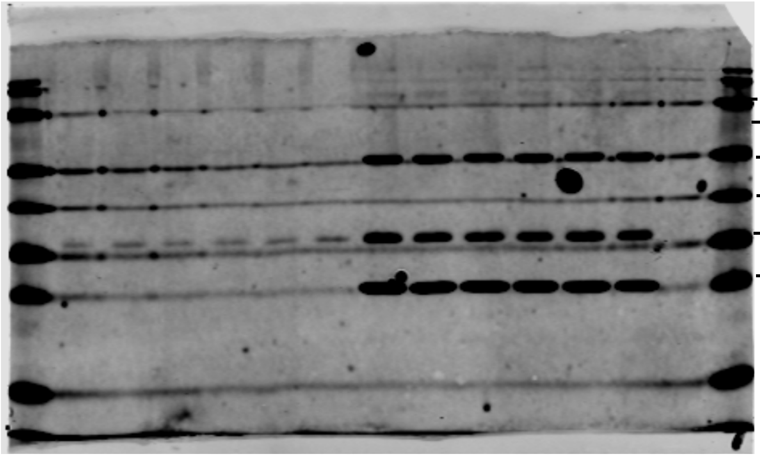

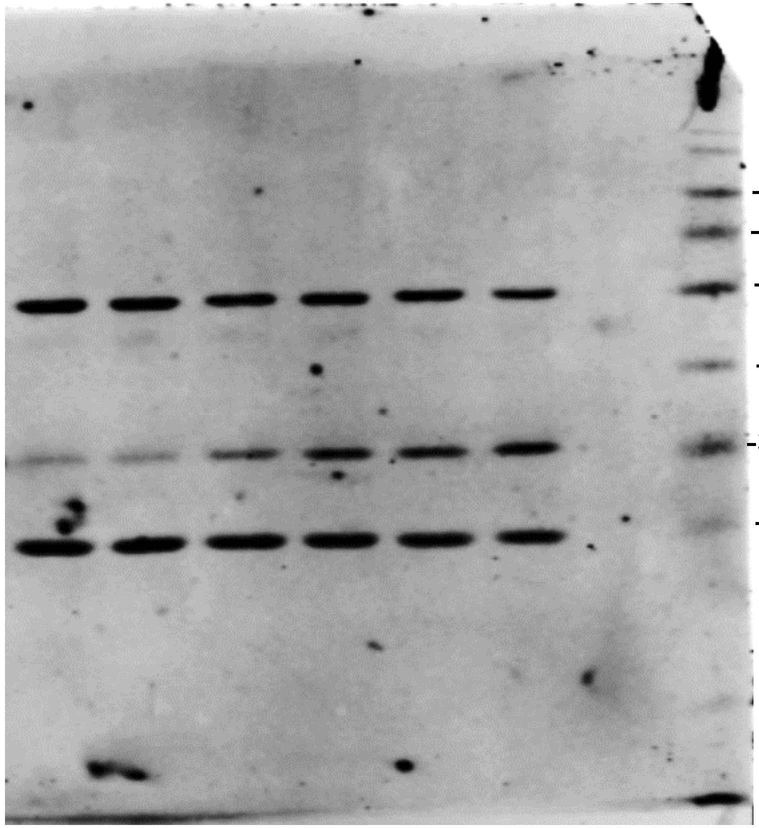

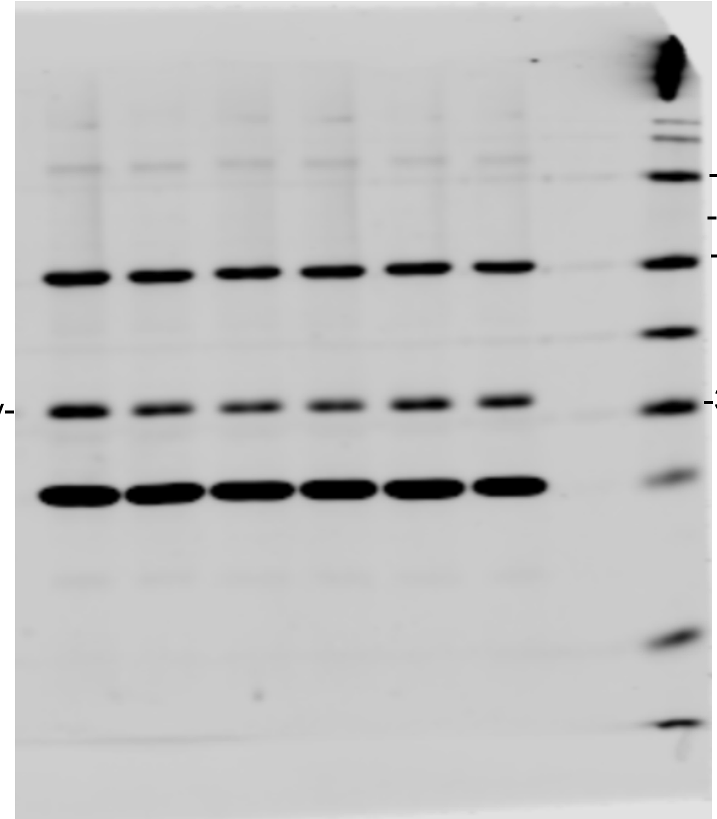

Full unedited blot/gel for Supplemental Figure 6B

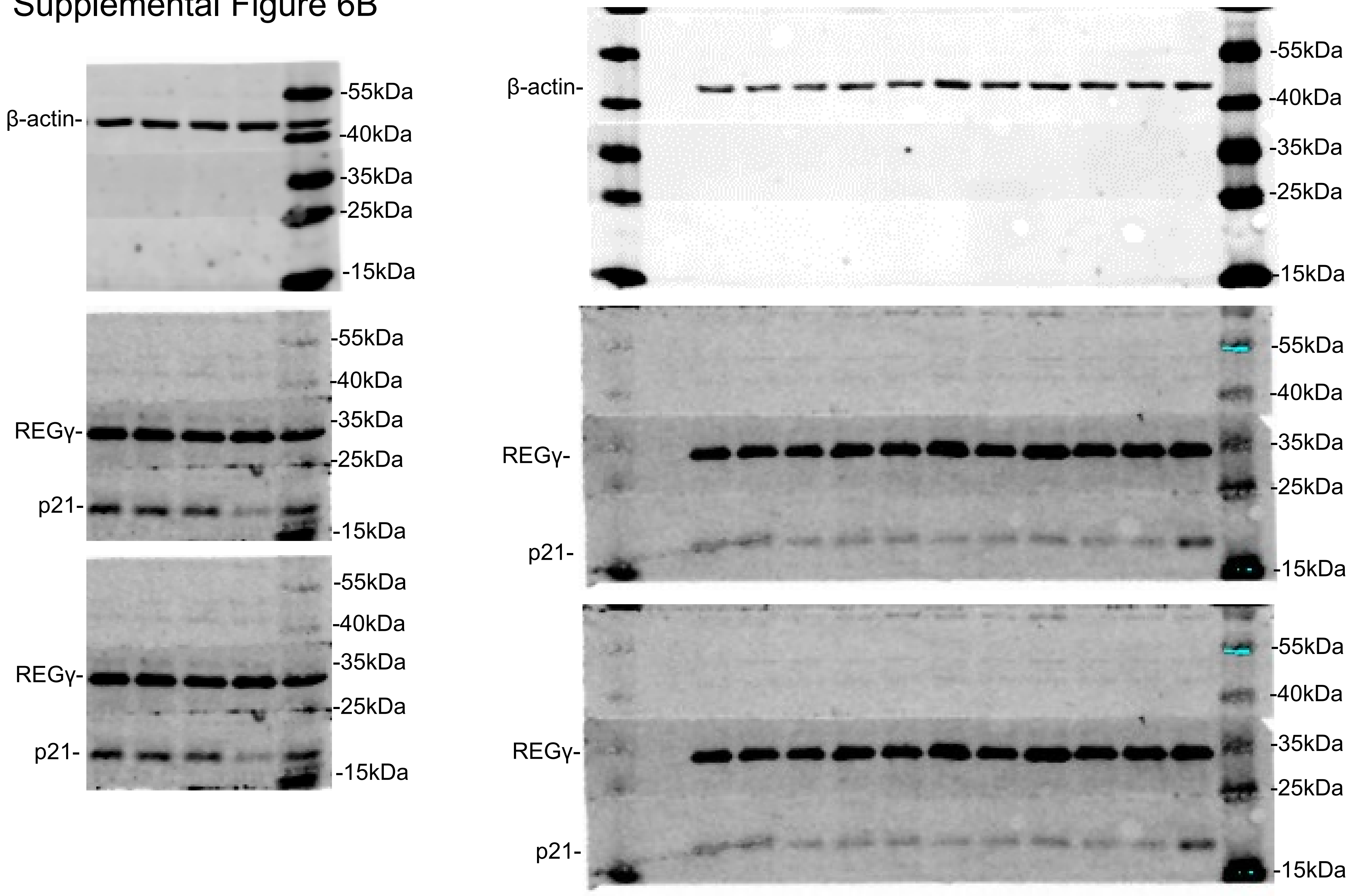

Full unedited blot/gel for

Supplemental Figure 6E

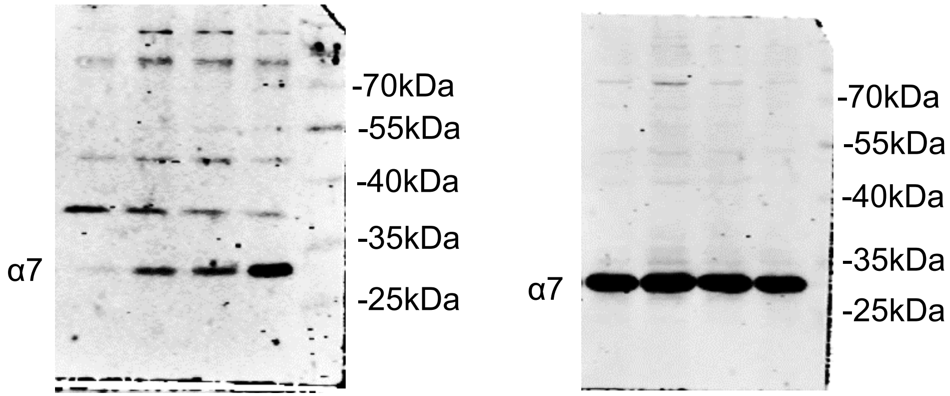

Supplemental Figure 6F

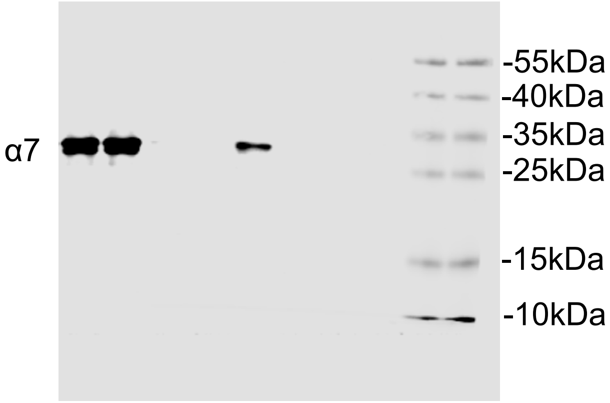

Supplemental Figure 7B

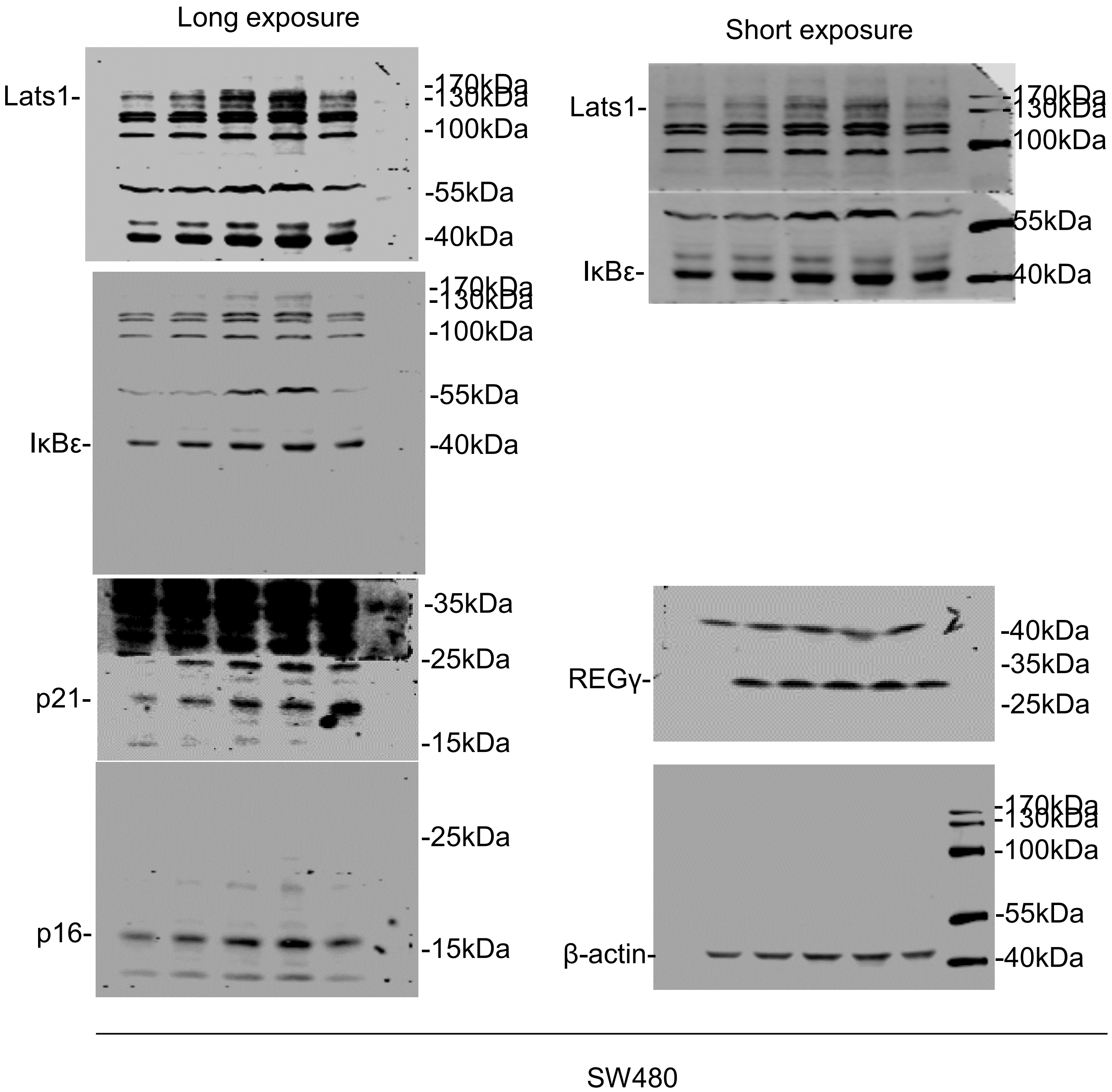

Supplement: Unedited blot and gel images [file jci-135-185278-s151.pdf]
